# An evolutionarily conserved mutual interdependence between Aire and microRNAs in promiscuous gene expression

**DOI:** 10.1002/eji.201343343

**Published:** 2013-04-16

**Authors:** Olga Ucar, Lars-Oliver Tykocinski, James Dooley, Adrian Liston, Bruno Kyewski

**Affiliations:** 1 Division of Developmental Immunology, German Cancer Research CenterHeidelberg, Germany; 2 Autoimmune Genetics Laboratory, VIBLeuven, Belgium; 3 Department of Microbiology and Immunology, University of LeuvenLeuven, Belgium

**Keywords:** Aire, gene expression, microRNA (miRNA), Thymic epithelium

## Abstract

The establishment and maintenance of central tolerance depends to a large extent on the ability of medullary thymic epithelial cells to express a variety of tissue-restricted antigens, the so-called promiscuous gene expression (pGE). Autoimmune regulator (Aire) is to date the best characterised transcriptional regulator known to at least partially coordinate pGE. There is accruing evidence that the expression of Aire-dependent and -independent genes is modulated by higher order chromatin configuration, epigenetic modifications and post-transcriptional control. Given the involvement of microRNAs (miRNAs) as potent post-transcriptional modulators of gene expression, we investigated their role in the regulation of pGE in purified mouse and human thymic epithelial cells (TECs). Microarray profiling of TEC subpopulations revealed evolutionarily conserved cell type and differentiation-specific miRNA signatures with a subset of miRNAs being significantly upregulated during terminal medullary thymic epithelial cell differentiation. The differential regulation of this subset of miRNAs was correlated with Aire expression and some of these miRNAs were misexpressed in the Aire knockout thymus. In turn, the specific absence of miRNAs in TECs resulted in a progressive reduction of Aire expression and pGE, affecting both Aire-dependent and -independent genes. In contrast, the absence of miR-29a only affected the Aire-dependent gene pool. These findings reveal a mutual interdependence of miRNA and Aire.

## Introduction

The scope of central tolerance in the thymus is largely dictated by the ectopic expression of a plethora of tissue-restricted antigens (TRAs) by thymic epithelial cells (TECs), in particular medullary thymic epithelial cells (mTECs), a phenomenon termed promiscuous gene expression (pGE). Within the mTEC lineage, the diversity and the level of pGE increase with terminal differentiation, which is characterised by the upregulation of CD80 and MHC class II (MHCII) and the induction of the autoimmune regulator (Aire). Thus, mature CD80^high^/MHCII^high^ mTECs express over 10% of all genes independently of tissue affiliation and tissue-specific transcription factors [1–3]. Aire is to date the only transcriptional regulator known to be involved in coordinating promiscuous expression of a set of TRAs. Its functional inactivation leads to severe autoimmune manifestations in men and to a lesser extent in mice [4–7]. Yet many self-antigens are expressed in TECs in an Aire-independent manner implying that additional factors also regulate pGE [8]. The exact molecular workings of Aire are still not well understood. Aire acts at the epigenetic level via binding to hypomethylated H3K4 residues, induces double-strand breaks in the DNA and enhances mRNA transcription and splicing [9–11]. More recently, it has been shown that *Aire*-binding sites are more widely distributed than predicted by selective binding to H3K4me0, i.e. *Aire*-binding sites in the genome largely overlap with Pol II promoter occupancy. In a fraction of these binding sites, *Aire* promoted the release of stalled Pol II thus enabling low-level transcription [12]. Our previous finding that pGE is characterised by a permissive chromatin configuration concurs with these observations [13]. Altogether, these studies present a strong case for epigenetic mechanisms and post-transcriptional modifications to regulate pGE.

MicroRNAs (miRNAs) are small RNA molecules involved in the post-transcriptional control of gene expression. Mature miRNAs bind Argonaute proteins and are incorporated into the RNA-induced silencing complex, which in turn binds to the target mRNA leading to translational block and/or degradation [14]. A primary miRNA transcript is first processed by Drosha in the nucleus and then by Dicer in the cytoplasm, and inactivation of either of these two enzymes blocks the biogenesis of mature miRNAs [15]. Various miRNAs have been shown to play important roles in development, cellular homeostasis and pathological conditions. Several autoimmune diseases are associated with aberrant miRNA expression, which presumably leads to hyperactivation of immune effector cells [16]. Misexpression of miRNAs in lymphocyte progenitors can affect lineage choice [17–19] and effector function [20]. The miRNA pathway is indispensable for T-cell development; thus, lack of Dicer compromises the survival of αβ-T cells and causes aberrant cytokine production [21, 22]. Dicer ablation in B cells leads to a partial block in the pro-B to pre-B transition [23]. Conditional deletion of Dicer in Treg cells results in a dramatic reduction in Treg-cell function and a lethal autoimmune disease [24].

T-cell development and function is also affected in a cell-extrinsic manner by miRNA loss in the thymic epithelium. Genetic deletion of Dicer in TECs results in premature thymic involution, progressive disorganisation of the thymic epithelium and formation of epithelial voids [25]. The epithelial defects manifest around weaning, and the ensuing decrease of the thymic T-cell export leads to an increased susceptibility to collagen-induced auto-immune arthritis [25]. Dicer-deficient animals exhibit no signs of spontaneous autoimmunity, but develop tissue infiltrates following restoration of the T-cell compartment after depletion of their neonatal T-cell pool [26]. The premature involution phenotype of Dicer mutants is recapitulated in mouse mutants lacking miR-29a; however, these animals do not display the same architectural disruption of the thymus [25], implying that other miRNAs regulate TEC maintenance.

Here, we analysed the role of miRNAs in mTEC biology and pGE. miRNA microarray analysis of TEC subpopulations revealed several epithelium-specific miRNAs that were upregulated upon mTEC maturation in mouse and human thymus. We show that, on the one hand, Aire regulates maturation-specific miRNA expression in mTECs and, on the other hand, miRNAs regulate Aire and TRA expression in mTECs. Collectively, our data document a mutual interdependence between Aire and miRNAs in directing mTEC maturation and pGE.

## Results

### TEC-specific miRNAs

**Figure 1:**
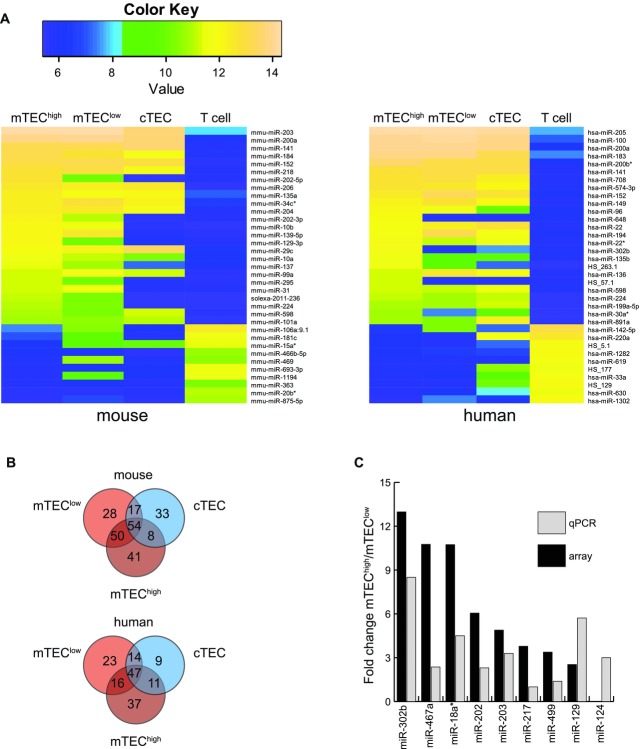
miRNAs are differentially expressed in thymic cell subsets. (A) Heat maps representing relative expression of selected miRNAs, ordered by their expression level in mTEC^high^. The top 25 shows the highest, the bottom 10, the lowest, ratio of expression value between mTEC^high^ and thymocytes in mouse (left) and human (right). For full lists of miRNA expression in mouse and human samples, see Supporting Information Tables 1 and 2. Data shown are generated from three (mouse) and two (human) independent experiments. (B) Venn diagrams showing overlap in miRNA expression between different thymic epithelial subsets in mouse and human. (C) Comparison of microarray and qPCR results for miRNAs expressed at higher levels in mTEC^high^ versus mTEC^low^ in both mouse and human (mouse data representative of three independent experiments are shown).

In order to investigate the miRNA signature in thymic epithelium, we performed miRNA microarray analysis of sorted thymic cells from mouse and human samples (for gating strategy, see Supporting Information Fig. 1A and B). We analysed thymocytes, cortical thymic epithelial cells (cTECs) and immature mTEC^low^ and mature mTEC^high^, the latter based on MHCII surface expression levels. Sets of 25 miRNAs with the highest and ten miRNAs with the lowest ratio in mTEC^high^ versus thymocytes of mouse and human are shown in Figure 1A.

A total of 231 (mouse) and 157 (human) miRNAs were upregulated (≥2-fold) in the TECs versus T cells. The analysis of TEC subtype specific miRNAs in both species showed a similar number of miRNAs expressed in all TECs (54 and 47). We found fewer miRNAs to be cortex specific (33 and 9) than medulla specific (119 and 76 in mouse and human, respectively). Of the medulla-specific miRNAs, the overlap between mTEC^high^ and mTEC^low^ was greater in the mouse (50) than in human (16). Conversely, the numbers of MHCII^low^- and MHCII^high^-specific miRNAs were comparable across species (Fig. 1B). TEC-specific miRNA expression was partially conserved between the two species. Thus, of 54 mouse and 47 human miRNAs expressed in all TECs, 22 were common to both species, whereas the overlap in cTEC-specific miRNAs was confined to miR-409–5p. Moreover, of 119 mouse and 76 human mTEC-specific miRNAs, eight were conserved in both species and in addition upregulated with mTEC maturation (Fig. 1C). The miRNAs that showed an upregulation with mTEC maturation were of particular interest in the context of this study, since mature CD80^high^/MHCII^high^ mTECs are characterised by the highest and most diverse promiscuous expression of TRAs [8]. We chose those miRNAs that were medulla-specific and upregulated in mTEC^high^ versus mTEC^low^ both in mouse and human samples and further validated their expression by qPCR in sorted mouse mTECs using 2- to 12-week old animals. miR-124, miR-129, miR-202, miR-203, miR-302b and miR-467a were stably expressed at two- to tenfold higher level in the mTEC^high^ subset independent of the maturation marker used for sorting the cells (Fig. 1C).

### miRNA expression fluctuates during TEC maturation and is deregulated in Aire-deficient thymus

**Figure 2:**
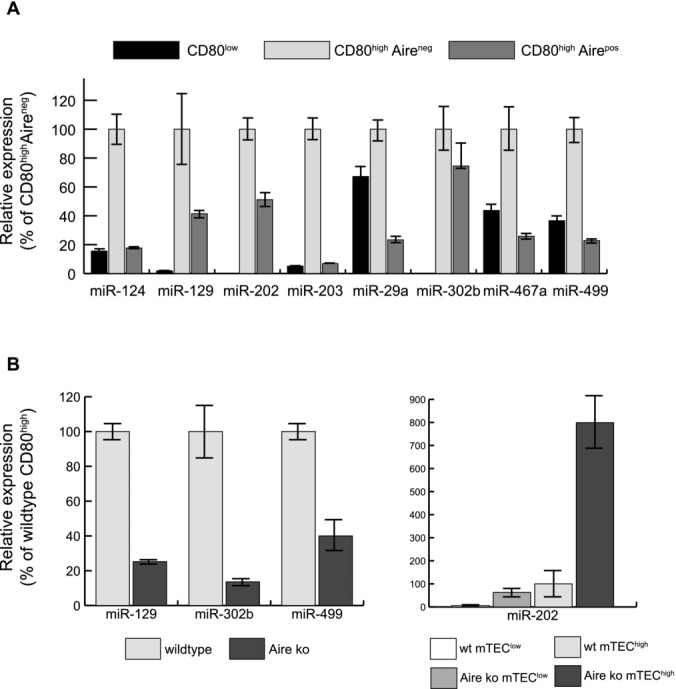
miRNA expression dynamics in mTEC subsets. (A) Candidate miRNA expression as measured by quantitative RT-PCR in sorted CD80^low^, CD80^high^Aire^neg^ and CD80^high^Aire^pos^ cells. Data are presented as the gene expression relative to the level in CD80^high^Aire^neg^ cells set to 100% and shown as mean ± SD of three technical replicates, representative of two independent experiments. (B) miR-129, miR-302b and miR-499 expression was downregulated (left), whereas miR-202 expression was upregulated (right) in Aire-deficient mutants as measured by quantitative RT-PCR in sorted CD80^low^ and CD80^high^ TECs of Aire-deficient mice and control littermates. Data are presented as the gene expression relative to the level in WT CD80^high^ and shown as mean ± SD of three technical replicates, representative of two independent experiments.

MHCII^high^/CD80^high^ mTECs can be further divided into Aire^neg^ and Aire^pos^ subsets (for gating strategy, see Supporting Information Fig. 1C). Aire-positive cells are characterised by upregulation of Aire-dependent TRAs and are considered the final maturation stage of mTECs. Hence, we asked whether miRNAs are differentially regulated according to Aire expression within the mTEC^high^ subset. Using the *Adig* transgenic mouse line [27], which harbours transgenic GFP expression under the control of the Aire promoter, we sorted GFP^neg^ (Aire^neg^) and GFP^pos^ (Aire^pos^) CD80^high^ mTECs and analysed the expression of TEC-specific miRNAs within these subsets. We found that all analysed miRNAs were downregulated in Aire^pos^ mTEC^high^ compared with Aire^neg^mTEC^high^ (Fig. 2A), with the exception of miR-302b, which showed no significant difference in expression levels. Next, we analysed TEC-specific miRNA expression in the mTECs of Aire null mutant mice [28]. Mir-129, miR-499 and miR-302b were expressed at similar levels in immature mTECs of mutant and control littermates, but were significantly downregulated in mature mTECs of Aire null mutants (Fig. 2B). miR-202 was affected in the opposite manner, being highly upregulated in both immature and mature mTECs of Aire null mutants (Fig. 2B). Together with the miRNA profiling, these data imply a mutual regulatory relationship between Aire and miRNAs expression during mTEC maturation.

### Lack of miRNAs in TECs leads to loss of pGE

Given the intricate regulation of different miRNAs in the mTEC compartment, we asked if and how miRNAs control the development of mTEC and pGE. To this end, we generated mice that lack mature miRNAs in TECs by crossing a conditional Dicer knockout line [21] with a FoxN1^Cre^ line [29] and analysed Dicer^flox/flox^×FoxN1^Cre^ (Dicer mutants) alongside with their Dicer^flox/flox^×FoxN1wt or Dicer^wt/wt^×FoxN1^Cre^ control littermates. We and others have recently shown that genetic deletion of Dicer in TECs leads to disruption of the thymic architecture in situ and premature involution [25, 26]. We analysed the thymi of Dicer mutant animals between 4 and 14 weeks of age for stromal cell integrity and Aire expression by immunohistology. We found that the phenotypic alterations set in around weaning (3–4 weeks of age), while the thymus was almost completely absent by 14 weeks of age. However, until 10 weeks of age the mutant thymi still possessed distinct cortical and medullary regions and Aire^pos^ cells in the medulla (Supporting Information Fig. 2).

**Figure 3:**
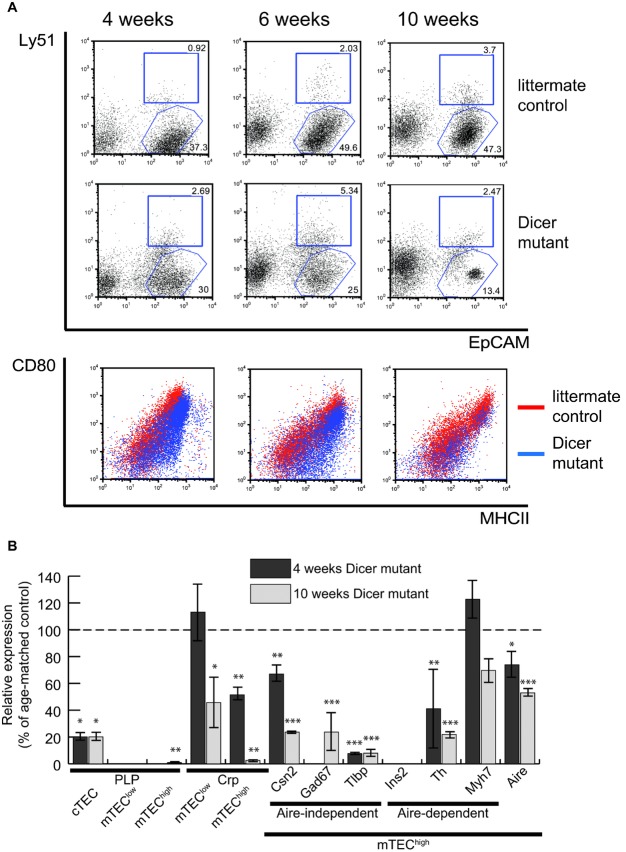
Lack of Dicer in the thymic epithelium leads to discordant maturation marker expression and a progressive loss of pGE. (A) Dicer mutants showed a decrease in the percentage of EpCAM-positive stromal cells, with mTECs (CD45^neg^ EpCAM^pos^ Ly51^neg^) being more affected than cTECs (CD45^neg^ EpCAM^pos^ Ly51^pos^). Dicer mutant mTECs showed a shift to lower CD80 and higher MHCII expression as compared with control littermates. Upper two rows show CD45^neg^ cells. Lower row shows CD45^neg^ EpCAM^pos^ Ly51^neg^ cells. Representative profiles of single animals of three samples are shown. (B) Dicer null mutants exhibited a progressive reduction of the levels of promiscuously expressed antigens, as demonstrated by quantitative RT-PCR on sorted TECs. Data are presented as percentage of the gene expression level obtained for the same subset of control littermates and shown as mean ± SD of three technical replicates, representative of two independent experiments. *p < 0.05, **p < 0.01, ***p < 0.001, Student's *t*-test.

Flow cytometric analysis of the composition of the thymic stromal cell compartment revealed a gradual decline in the numbers of EpCAM-positive epithelial cells in the mutants. The medulla was more severely affected (Fig. 3A). Moreover, the almost linear relationship in upregulation of CD80 and MHCII during mTEC differentiation as observed in WT mice was skewed in mutant mice. The analysis of single thymi from Dicer mutants and littermates of different ages (4, 6 and 10 weeks) showed that CD80 expression was shifted to lower, and MHCII to higher expression levels in Dicer-deficient mTECs (Fig. 3A). Progressive loss of TECs in Dicer null mutants documents that miRNAs are essential for thymic stroma integrity and maintenance.

Next, we asked whether premature involution and stroma disorganisation would affect the cell-intrinsic regulation of pGE in Dicer mutants, and whether this effect progressed with age. To this end, we isolated immature and mature mTECs from 4- and 10-week-old Dicer-deficient animals and control littermates. Because the CD80 and MHCII expression profiles were slightly shifted in the mutants, relative gates were chosen, i.e. the lower/upper 35% of cells based on the expression levels of MHCII was taken as immature/mature mTECs, respectively. Based on our previous work [8], we chose representative TRAs of the four pools of promiscuously expressed TRAs: (i) PLP (proteolipid protein, expressed in all TECs); (ii) Crp (C-reactive protein, expressed in all mTECs); (iii) Csn2 (casein beta), Gad67 (glutamate decarboxylase 1 isoform) and Tlbp (testis lipid-binding protein, all upregulated in mTEC^high^, Aire independent); (iv) Ins2 (insulin 2), Th (tyrosine hydroxylase) and Myh7 (myosin heavy chain 7, all upregulated in mTEC^high^, Aire dependent).

As shown in Fig. 3B, most genes tested were substantially downregulated in mutant TECs already by 4 weeks compared with those of control animals, and this was more pronounced by 10 weeks of age. The observed decline of pGE was independent of the maturation marker used for isolating the subsets, i.e. CD80 or MHCII. Notably, this downregulation affected all four pools of pGE. Since the expression analysis was normalised to purified mTEC subsets, the data show that miRNAs influence the cell-intrinsic control of pGE beyond the premature involution of the thymus, i.e. the selective loss of TEC subsets.

**Figure 4:**
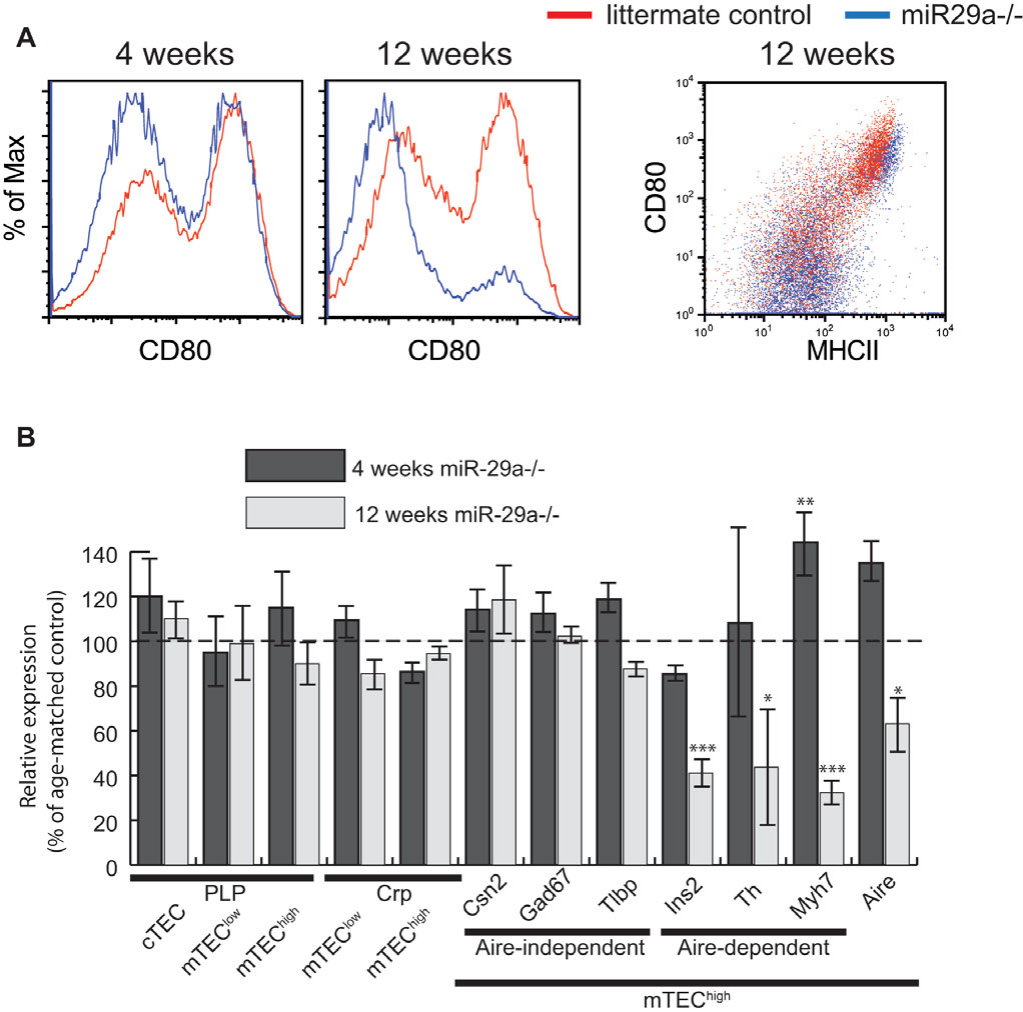
Reduction in Aire-dependent pGE in miR-29a-deficient mice. (A) miR-29a null mTECs showed normal CD80 maturation profile at 4 weeks of age and a shift to lower CD80 and higher MHCII expression as compared with control littermates at 12 weeks. Flow cytometry plots shown are representative of *n* > 3 mice analysed in two independent experiments. (B) Expression of Aire and Aire-dependent genes was reduced in miR-29a null mutant mice at 12 weeks (but not at 4 weeks) of age, whereas Aire-independent genes expression is comparable with that of control littermates. Data are presented as percentage of the gene expression level obtained for the same subset of control littermates and are shown as mean ± SD of three technical replicates, representative of two independent experiments. *p < 0.05, **p < 0.01, ***p < 0.001, Student's *t*-test.

Rapid premature thymic involution in Dicer null mutants can be in part attributed to the lack of mature miR-29a [25]. The epithelial cell architecture of miR-29a-deficient thymi, however, did not show the typical architectural changes that were observed in Dicer null mutants. In order to determine whether the lack of this particular miRNA had an effect on pGE, we isolated mTECs from miR-29a-deficient mutants and WT littermates at 4 and 12 weeks of age. At 4 weeks of age, miR-29a mutant TECs showed a normal maturation profile (based on CD80 surface marker staining), and the relative numbers of mature and immature mTECs were comparable with those of their WT littermates (Fig. 4A). Only at 12 weeks of age, we observed a reduction of mature CD80^high^ mTECs in the mutants and a shift towards lower CD80 and higher MHCII expression levels, similar to the Dicer null mutants (Fig. 4A).

When we analysed the expression of the four pools of TRAs mentioned above, we observed a reduction in the expression of Aire-dependent genes (Ins2, Th and Myh7) in the 12-week-old mutants (Fig. 4B). The expression of Aire-independent TRAs, such as Csn2, Gad67, Tlbp, Crp and PLP, however, was unaltered in miR-29a null mutants of both ages compared with that of age-matched WT littermates (Fig. 4B). The decrease in expression of Aire-dependent genes correlated with the decrease of Aire expression in 12-week-old animals. These data show that selective lack of miR-29a as opposed to a complete lack of mature miRNAs in TECs only affects a subset of promiscuously expressed genes and this phenotype emerges at a more advanced age.

## Discussion

pGE in TECs is regulated at multiple levels [7–13]. In addition to the transcriptional regulator Aire, epigenetic mechanisms and post-transcriptional expression control have been implicated [8, 13]. Here, we analysed the miRNome of cTEC and mTEC and the impact of miRNAs on the interdependence of TEC development and pGE. Our study shows (i) that miRNAs are dynamically regulated during mTEC differentiation, (ii) miRNA expression in TECs is under control of Aire, (iii) conversely lack of mature miRNAs in TECs globally affects pGE and Aire, (iv) lack of miR-29a selectively affects Aire-dependent pGE and (v) lack of mature miRNAs in TECs inversely affects expression levels of the ‘maturation markers’ CD80 and MHCII.

We identified miRNAs with TEC-specific expression in mouse and human TECs and demonstrated that there is a larger overlap in miRNA expression between immature and mature mTECs than between either of these and cTECs. Several of the mTEC-specific miRNAs showed a maturation-dependent upregulation in both mouse and human, compatible with a potential role in pGE regulation. Hence, we focused our analysis on these particular miRNAs. Interestingly, miR-124, miR-129, miR-202, miR-203, miR-302b and miR-467a were differentially regulated in immature and mature Aire^neg^ versus mature Aire^pos^ mTEC subsets. Thus, miR-129 and miR-499 expression was downregulated upon induction of Aire among mature mTECs, yet both miRNAs were also downregulated in mature mTECs in Aire null mice as compared with WT. These data argue against a direct control of miRNA expression by Aire. A role of Aire beyond the control of pGE has been previously described, e.g. in antigen processing [30], DC homing [31] and mTEC differentiation [32].

In the context of a putative role of miRNA in pGE, it is noteworthy that several mRNAs, upregulated upon mTEC maturation, showed tissue-specific expression patterns, i.e. being restricted to brain (miR-124 and miR-129), heart (miR-499), testis (miR-202), skin (miR-203) or embryo (miR-467 and miR-302). Moreover, miR-124 and miR-203 have been shown to be upregulated upon terminal differentiation in neurons and keratinocytes, respectively [33, 34]. In this context, it will be interesting to analyse whether miRNAs are also expressed in a mosaic pattern in mTECs as TRAs [2].

A possible functional relationship between pGE and miRNA has been previously addressed by analysing the impact of an Aire knockdown on the miRNome in an mTEC cell line [35]. The miRNAs reported by the authors as Aire-dependent in vitro, however, showed little overlap with our ex vivo microarray data. The majority of miRNAs that showed Aire dependency in cell culture were either not expressed in TECs or expressed at similar levels in TECs and T cells with two exceptions; miR-200b, which was TEC specific, but not maturation dependent, and miR-9, which was only expressed in mature mTECs in the mouse, but not in human. As Aire targets different sets of genes depending on the cellular context [17, 36, 37], the observed discrepancies between freshly isolated TECs and an established cell line were not unexpected.

Our results indicate that miRNAs regulate Aire expression, as Aire is downregulated in mature mTECs of Dicer null mutants. Together with Aire, the expression of various Aire-dependent and -independent TRAs was affected in the Dicer null mutants. Notably, the loss of Dicer affected all four pools of TRAs, which according to a recent study are differentially regulated in a cell-autonomous or non-autonomous manner [38]. Since our analysis was normalised to TEC subsets, this points to a cell-intrinsic reduction of pGE. A reduction in ectopic expression of certain TRAs in unseparated mTECs from 2-week-old Dicer null mice had been previously observed [26]; however, this analysis did not discriminate between cell-intrinsic effects versus altered TEC subset composition.

The age-dependent decline in pGE can also not be explained by a gradual disorganisation of the thymic architecture, since it sets in before the appearance of epithelial voids in the mutant thymus. Previously described mutants with disrupted thymic architecture did not display changes in pGE when normalized to purified TEC subsets [39–42]. Thus, only the Dicer and the Aire mutants dissociate pGE from mTEC differentiation. The discordant effects on CD80 and MHCII upregulation along mTEC differentiation in the absence of Dicer or miR-29a point to subtle differences in the role of miRNAs in the regulation of these two surface receptors.

A first step in identifying miRNAs that cause the observed Dicer knockout thymic phenotypes was a side-by-side comparison with miR-29a null mutant mice. These mice mimic the Dicer null-associated thymic involution without presenting the gross architectural abnormalities [25]. Interestingly, in miR-29a null mutant mice, the effects on pGE were confined to the Aire-dependent gene set. This implies that the different gene pools encompassing pGE [8] are subject to differential control by miRNAs.

In conclusion, we have analysed the expression of miRNAs in the thymic epithelium and at different stages of mTEC maturation and the role of miRNA biogenesis in pGE. Our data document that particular miRNA signatures are maturation dependent and species conserved, coincide with Aire upregulation and are affected by Aire loss. Aire expression and pGE, in turn, are affected by the loss of Dicer. The comparison of Dicer and miR-29a mutants suggests that the defects in thymic involution, epithelial architecture and pGE in the Dicer null mutants are caused by the absence of miRNAs other than miR-29a. The identification of these mRNA species poses a future challenge.

## Materials and methods

### Animals and human samples

When not indicated otherwise, 4- to 6-week-old WT C57BL6/N mice were used. Dicer^flox/flox^ mice have been provided by M. Merkenschlager [21], FoxN1^Cre^ transgenic mice by T. Boehm [29] and *Adig* (Aire-GFP) mice on the BALB/c background by L. Klein [27]. Dicer null mutant (Dicer^flox/flox^×FoxN1^Cre^) and miR-29a knockout mouse lines were kept on the C57BL6/N background. All animal breeding and experiments were performed according to the guidelines of the German Cancer Research Center (DKFZ).

Human thymi were obtained from two male children (5 and 12 months old) in the course of corrective cardiac surgery at the Department of Cardiac Surgery, Medical School of the University of Heidelberg, Germany. Human studies were approved by the Institutional Review Board of the University of Heidelberg.

### TEC isolation and sorting

TEC isolation was performed as previously described [13, 43, 44]. Thymi were minced and transferred to round-bottom tubes for initial thymocyte release in RPMI medium (3% FCS, 1 mM HEPES), after which the remaining tissue was digested for several rounds with collagenase and collagenase/dispase (for human samples, collagenase/dispase and trypsin, respectively) under continuous stirring. Cells collected from the collagenase/dispase (mouse) or trypsin (human) fractions were depleted of CD45^pos^ cells using CD45 MicroBeads (Miltenyi Biotec). Murine TEC-enriched single-cell suspensions were blocked with anti-FcR mAb 2.4G2 and stained for the following surface markers: PerCP-anti-CD45 (clone 30F11, BD Pharmingen), Alexa647-anti-EpCAM (clone G8.8), FITC-anti-Ly-51 (clone 6C3, BD Pharmingen), PE-anti-CD80 (clone 16–10A1, BD Pharmingen), biotin-anti-MHCII (clone M5/114.15.2, BD Pharmingen) and Sav-PE-Cy7 (BD Biosciences). For dead cell exclusion, PI or DAPI were used. Mouse TECs were defined as CD45^neg^ EpCAM^pos^, and were further subdivided into Ly-51^pos^ cTECs and Ly-51^neg^ mTECs. mTECs were separated into immature (CD80^low^ or MHCII^low^) and mature (CD80^high^ or MHCII^high^), representing the top and bottom 33–35% of the population. In Adig mice, mature CD80^high^ mTECs were further separated into Aire^neg^ and Aire^pos^ subsets based on Aire-driven GFP expression. Cell sorting was performed on a BD FACSAria cell sorter.

After CD45 depletion, human TEC-enriched cells were blocked with mouse serum and stained with anti-CD45-PerCP (clone 2D1, BD Biosciences), biotinylated anti-EpCAM (clone HEA125), anti-HLA-DR-Alexa647 (clone L243, both gifts of G. Moldenhauer, DKFZ, Heidelberg), CDR2-Alexa488 [45] and streptavidin-PE (BD Biosciences). cTECs (CD45^−/lo^CDR2^+^G8.8^+^), immature mTECs (CD45^−/lo^CDR2^−^HEA125^+^HLA-DR^low^) and mature mTECs (CD45^−/lo^CDR2^−^HEA125^+^HLA-DR^hi^) were sorted on a BD FACSAria cell sorter.

### Isolation of total RNA, Illumina microarray analysis and qPCR

Total RNA was isolated using the High Pure miRNA Isolation kit (Roche) and the concentration and integrity were analysed by Eukaryote Total RNA nano Series II chip on a 2100 Bioanalyzer (Agilent). A total of 200 ng of total RNA was polyadenylated and used for cDNA synthesis. miRNA expression profiling was performed using Illumina Version 2 MicroRNA Expression BeadChip Arrays according to the manufacturer's instructions. The human v2 and the mouse v2 panels contained 1146 and 656 probes, respectively. Arrays were scanned on a BeadArray Reader and the array intensity data were imported into BeadStudio v3.2 (Illumina).

Background adjustment and quantile normalisation was performed with the BeadStudio software. All sample expression values are mean values over beads and group expression values are mean values over the replicates expression.

For RT-qPCR analysis, RNA was prepared from sorted cells by modified Trizol extraction method. The modification included precipitation with 100% Ethanol at −20°C overnight. For messenger RNA analysis, 100–500 ng total RNA was used for the first-strand synthesis with SuperScriptII and oligo-dT primer. For miRNA analysis, first-strand synthesis was performed with commercially available Taqman miRNA Assay primers according to the manufacturer's protocol. qPCR was performed in triplicates using the Applied Biosystems 7300 light cycler with Taqman probes for miRNAs and intron-spanning primers for mRNA-coding genes. The results were analysed by the ddCt method. Expression levels were quantified from three technical replicates using Student's *t*-test, and for each quantification at least two independent experiments were performed.

## Abbreviations

Aire: autoimmune regulator
cTEC: cortical thymic epithelial cell
MHCII: MHC class II
miRNA: microRNA
mTEC: medullary thymic epithelial cell
mTEC^high^: CD80^high^/MHCII^high^ medullary thymic epithelial cell
mTEC^low^: CD80^low^/MHCII^low^ medullary thymic epithelial cell
pGE: promiscuous gene expression
TEC: thymic epithelial cell
TRA: tissue-restricted antigen

## Supplementary material

As a service to our authors and readers, this journal provides supporting information supplied by the authors. Such materials are peer reviewed and may be re-organized for online delivery, but are not copy-edited or typeset. Technical support issues arising from supporting information (other than missing files) should be addressed to the authors.

**Figure S1. Gating strategy used for TEC sorting**. **(A)** Mouse TEC-enriched cell suspensions were sorted as follows: PI-negative, CD45pos cells were sorted as thymocytes. PI-negative, CD45neg cells were separated into EpCAMposLy51high (cTECs) and EpCAMposLy51low (mTECs). mTECs were further separated into MHClow and MHChigh. **(B)** Human TEC-enriched cell suspensions were sorted as follows: DAPI-negative, CD45pos cells were sorted as thymocytes. DAPI-negative, CD45neg cells were separated into EpCAMposCDR2high (cTECs) and EpCAMposCDR2low (mTECs). mTECs were further separated into MHClow and MHChigh. **(C)** Mouse TEC-enriched cell suspensions derived from *Adig* mice were sorted as follows: PI-negative, CD45pos cells were sorted as thymocytes. PI-negative, CD45negEpCAMpos cells were separated into CD80lowGFPlow (CD80low), CD80highGFPlow (CD80highAireneg) and CD80highGFPhigh (CD80highAirepos).

**Figure S2. Progressive structural disorganisation of Dicerdeficient mutant thymi**. Thymic sections obtained from 4-, 10- and 14-week old Dicer null mutants and wildtype littermate controls were stained for cytokeratin-14, cytokeratin-8 and Aire. Epithelial voids are indicated with white asterisks. Note that the medulla disorganisation progressed with age, yet Airepos cells were still numerous in the Dicer mutants at 10 weeks. At 14 weeks of age, the Dicer null mutant thymus was very small and only a few cytokeratin-14-positive cells were detected. Aire staining is therefore not included for this age.
