# Supplementary material for: An evolutionarily conserved mutual interdependence between Aire and microRNAs in promiscuous gene expression
Source: Eur J Immunol. 2013 Apr 16;43(7):1769–78. doi: 10.1002/eji.201343343 (PMC3816332; doi:10.1002/eji.201343343)
Supplement: Supplementary file 2 [file eji0043-1769-SD2.pdf]

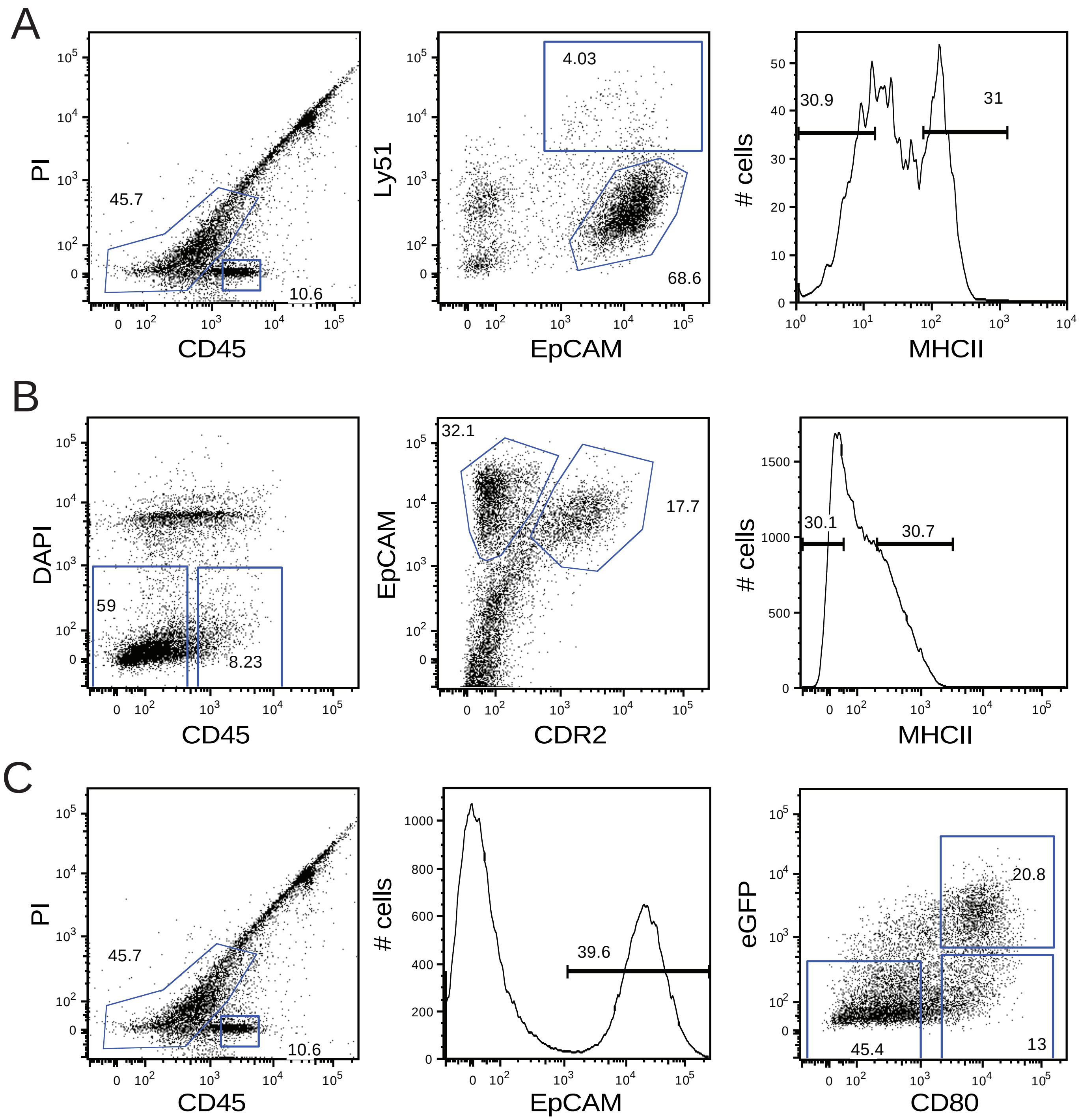

Supporting Information Figure 1. Gating strategy used for TEC sorting.

(A) Mouse TEC-enriched cell suspensions were sorted as follows: PI-negative, CD45^{pos} cells were sorted as thymocytes. PI-negative, CD45^{neg} cells were separated into EpCAM^{pos}Ly51^{high} (cTECs) and EpCAM^{pos}Ly51^{low} (mTECs). mTECs were further separated into MHC^{low} and MHC^{high}.

(B) Human TEC-enriched cell suspensions were sorted as follows: DAPI-negative, CD45^{pos} cells were sorted as thymocytes. DAPI-negative, CD45^{neg} cells were separated into EpCAM^{pos}CDR2^{high} (cTECs) and EpCAM^{pos}CDR2^{low} (mTECs). mTECs were further separated into MHC^{low} and MHC^{high}.

(C) Mouse TEC-enriched cell suspensions derived from *Adig* mice were sorted as follows: PI-negative, CD45^{pos} cells were sorted as thymocytes. PI-negative, CD45^{neg}EpCAM^{pos} cells were separated into CD80^{low}GFP^{low} (CD80^{low}), CD80^{high}GFP^{low} (CD80^{high}Aire^{neg}) and CD80^{high}GFP^{high} (CD80^{high}Aire^{pos}).

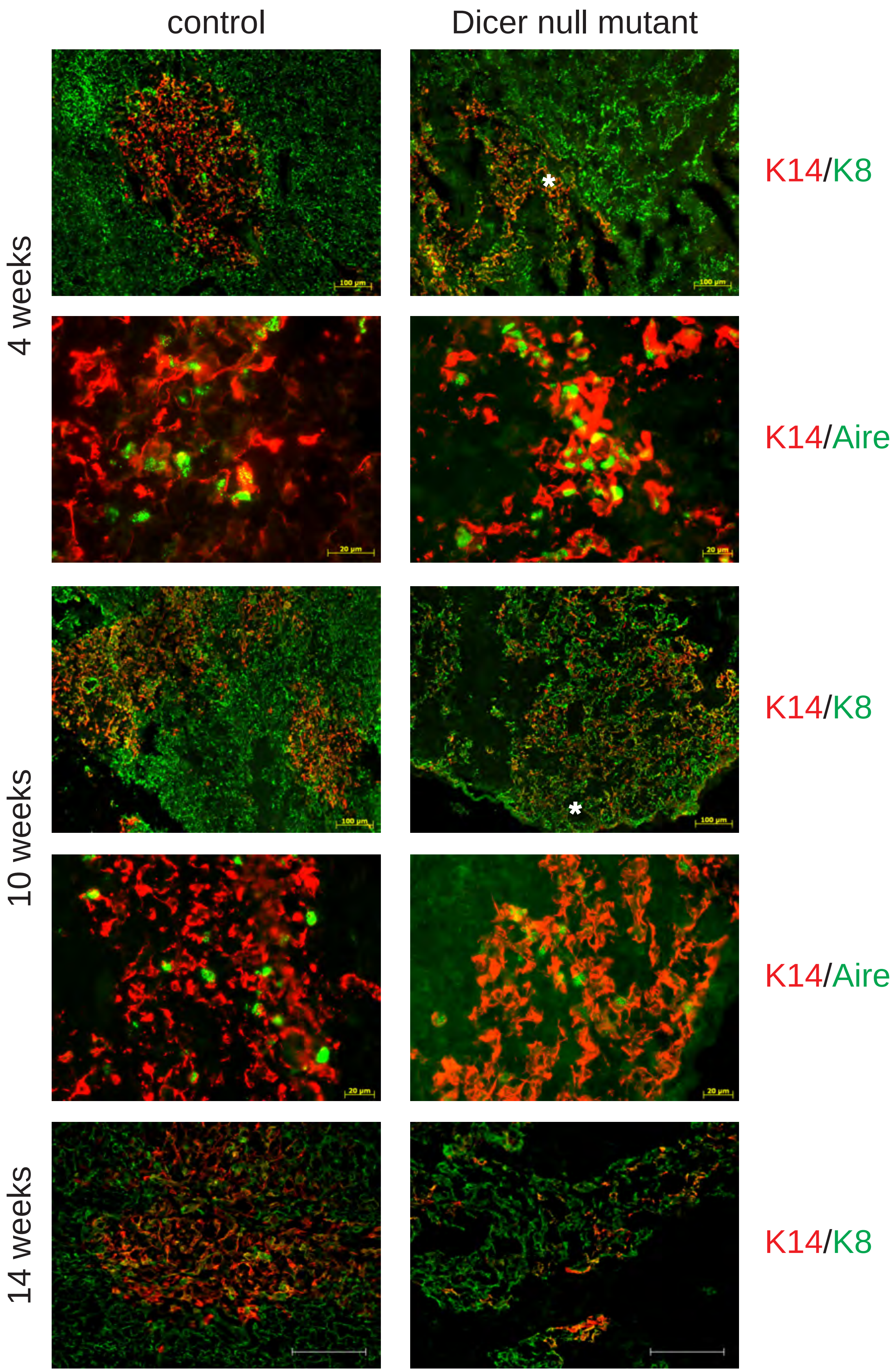

Supporting Information Figure 2. Progressive structural disorganisation of Dicer-deficient mutant thymi.

Thymic sections obtained from 4-, 10- and 14-week old Dicer null mutants and wildtype littermate controls were stained for cytokeratin-14, cytokeratin-8 and Aire. Epithelial voids are indicated with white asterisks. Note that the medulla disorganisation progressed with age, yet Aire^{pos} cells were still numerous in the Dicer mutants at 10 weeks. At 14 weeks of age, the Dicer null mutant thymus was very small and only a few cytokeratin-14-positive cells were detected. Aire staining is therefore not included for this age.

Supporting Information Table 1. miRNA expression in murine sorted thymic cell populations
(average of three biological replicates)

	Tc		mTEC_low		mTEC_high		cTEC	
TargetID	Avg_signal	p-val	Avg_signal	p-val	Avg_signal	p-val	Avg_signal	p-val
mmu-let-7a	17943.05	3.678E-38	17363.22	3.678E-38	16107.52	3.678E-38	17585.89	3.678E-38
mmu-let-7b	20531.83	3.678E-38	21261.96	3.678E-38	21184.63	3.678E-38	21511.33	3.678E-38
mmu-let-7b*	294.8889	3.678E-38	87.22222	2.31044E-12	126.3704	3.678E-38	298.7778	3.678E-38
mmu-let-7c	14461.11	3.678E-38	16423.7	3.678E-38	15772.74	3.678E-38	18275	3.678E-38
mmu-let-7c-1*	47.44444	0.7310847	48.07407	0.6184288	46.07407	0.7133024	240.7778	3.678E-38
mmu-let-7c-2*,mmu-let-7a*	718.1111	3.678E-38	1031.37	3.678E-38	2472.222	3.678E-38	82	4.01494E-05
mmu-let-7d	18460.61	3.678E-38	15540	3.678E-38	15980.56	3.678E-38	10758.78	3.678E-38
mmu-let-7d*	12957.61	3.678E-38	9739.111	3.678E-38	8060.333	3.678E-38	2743.111	3.678E-38
mmu-let-7e	3235.167	3.678E-38	12535.67	3.678E-38	11019.37	3.678E-38	10038.11	3.678E-38
mmu-let-7f	17093.78	3.678E-38	17907.59	3.678E-38	17331.33	3.678E-38	15162	3.678E-38
mmu-let-7f*	704.9445	3.678E-38	83.18519	4.64162E-10	64.2963	0.001034811	372.7778	3.678E-38
mmu-let-7g	16685.89	3.678E-38	15844.48	3.678E-38	15672	3.678E-38	16467.89	3.678E-38
mmu-let-7g*	647.0555	3.678E-38	622.5555	3.678E-38	75.88889	1.79745E-08	432.1111	3.678E-38
mmu-let-7i	14841.67	3.678E-38	12635.59	3.678E-38	12915.89	3.678E-38	10408.78	3.678E-38
mmu-let-7i*	2147.722	3.678E-38	202.1481	3.678E-38	200.2963	3.678E-38	59.44444	0.1945091
mmu-miR-1	3777.222	3.678E-38	2666.556	3.678E-38	895.7778	3.678E-38	5185.667	3.678E-38
mmu-miR-100	419.3889	3.678E-38	11906.52	3.678E-38	8423.667	3.678E-38	14031.89	3.678E-38
mmu-miR-101a	44.44444	0.8550847	1535.852	3.678E-38	1768	3.678E-38	2127.444	3.678E-38
mmu-miR-101a*	477.7222	3.678E-38	1007.074	3.678E-38	94.81481	2.42684E-27	1360.778	3.678E-38
mmu-miR-101a:9.1	281.8889	3.678E-38	1583.148	3.678E-38	1147.222	3.678E-38	3061.444	3.678E-38
mmu-miR-101b	1698.611	3.678E-38	442.963	3.678E-38	1670.593	3.678E-38	1776.667	3.678E-38
mmu-miR-101b:9.1	1048.333	3.678E-38	1136.481	3.678E-38	1779.63	3.678E-38	46.77778	0.8077124
mmu-miR-103	8478.167	3.678E-38	9524.63	3.678E-38	9742.593	3.678E-38	6653.444	3.678E-38
mmu-miR-105	54.66667	0.4187606	400.4815	3.678E-38	49.85185	0.4212417	50.11111	0.6605622
mmu-miR-106a	8732	3.678E-38	1269.444	3.678E-38	2498.037	3.678E-38	7044.889	3.678E-38
mmu-miR-106a:9.1	5641.889	3.678E-38	1447.556	3.678E-38	182.2222	3.678E-38	73.88889	0.00228971
mmu-miR-106b	11808.5	3.678E-38	7089.556	3.678E-38	8295.926	3.678E-38	9735.444	3.678E-38
mmu-miR-106b*	1312.611	3.678E-38	967.5926	3.678E-38	1945.407	3.678E-38	46.66667	0.8118355
mmu-miR-107	309.7222	3.678E-38	480.3704	3.678E-38	207	3.678E-38	95.44444	3.72896E-09
mmu-miR-10a	65.11111	0.0469448	2392.778	3.678E-38	3096.259	3.678E-38	1250.444	3.678E-38
mmu-miR-10a*	379	3.678E-38	306.5185	3.678E-38	119.8519	3.678E-38	443.6667	3.678E-38
mmu-miR-10b	41.94444	0.9188876	2846.556	3.678E-38	4541.074	3.678E-38	46.77778	0.8077124
mmu-miR-10b*	369.3889	3.678E-38	75.7037	6.1653E-07	94.40741	7.66403E-20	64.66666	0.05762657
mmu-miR-1186	2500.556	3.678E-38	568.6667	3.678E-38	921.3333	3.678E-38	670.5555	3.678E-38
mmu-miR-1187	4408.944	3.678E-38	2917.296	3.678E-38	3020.037	3.678E-38	3021.111	3.678E-38
mmu-miR-1188	957.6111	3.678E-38	57.66667	0.03866006	49.07407	0.4873777	51.22222	0.6034028
mmu-miR-1190	169.7222	3.678E-38	130.8519	3.678E-38	196.2593	3.678E-38	260.8889	3.678E-38
mmu-miR-1191	2946.111	3.678E-38	139.5556	3.678E-38	548.7778	3.678E-38	65	0.05255555
mmu-miR-1192	97.77778	7.45305E-09	67.59259	0.000506392	69.77778	1.7046E-05	180.5556	3.678E-38
mmu-miR-1193	108.3889	1.62995E-11	64.92593	0.001238427	63.92593	0.002278855	184.3333	3.678E-38
mmu-miR-1194	3217.5	3.678E-38	490.037	3.678E-38	51.14815	0.316971	59.66667	0.1862592
mmu-miR-1195	16166.22	3.678E-38	14905.85	3.678E-38	16741.52	3.678E-38	13626.44	3.678E-38
mmu-miR-1196	1235.778	3.678E-38	452.3704	3.678E-38	119.5185	3.678E-38	41.66667	0.9415511
mmu-miR-1197	971.6111	3.678E-38	326.4815	3.678E-38	107.3333	7.47892E-32	1495.556	3.678E-38
mmu-miR-1198	1284.944	3.678E-38	1745.963	3.678E-38	2008.889	3.678E-38	44.55556	0.8796347
mmu-miR-1199	77.88889	0.001043785	233.5185	3.678E-38	89.81481	4.57431E-16	1104.556	3.678E-38
mmu-miR-122	2676.611	3.678E-38	941.9259	3.678E-38	948.3704	3.678E-38	410.5555	3.678E-38
mmu-miR-1224	171.5556	3.678E-38	691.7037	3.678E-38	64.66666	0.000830975	108.8889	1.29974E-14
mmu-miR-124	53.05556	0.4590458	613.7407	3.678E-38	1002	3.678E-38	73	0.003326249
mmu-miR-124*	46	0.809223	43.59259	0.8815027	44.33333	0.8142536	45	0.8670232
mmu-miR-125a-3p	47.94444	0.7126541	381.4074	3.678E-38	70.7037	1.90156E-06	49.44444	0.6932549
mmu-miR-125a-5p	507.5	3.678E-38	16075.85	3.678E-38	12516.48	3.678E-38	15684.11	3.678E-38
mmu-miR-125b*	1013.611	3.678E-38	232.7778	3.678E-38	110.2222	4.55007E-33	1270.333	3.678E-38
mmu-miR-125b-3p	166.0556	4.8313E-35	722	3.678E-38	126.9259	3.678E-38	149.7778	3.678E-38
mmu-miR-125b-5p	1643.389	3.678E-38	19619.55	3.678E-38	16118.7	3.678E-38	15831.33	3.678E-38
mmu-miR-126-3p	1499.278	3.678E-38	5493.667	3.678E-38	6355.111	3.678E-38	1726.778	3.678E-38
mmu-miR-126-5p	904.8889	3.678E-38	8336.481	3.678E-38	7008.371	3.678E-38	11253.56	3.678E-38
mmu-miR-127	48.27778	0.6974257	46.03704	0.7692794	411.8889	3.678E-38	45.11111	0.8637331
mmu-miR-127*	48	0.7323169	46.07407	0.759453	46.18518	0.7009633	44.55556	0.8796347
mmu-miR-128	16017.94	3.678E-38	7845.259	3.678E-38	7797.259	3.678E-38	12363.44	3.678E-38
mmu-miR-128a:9.1	10298.44	3.678E-38	3460.667	3.678E-38	2795.204	3.678E-38	5115.278	3.678E-38
mmu-miR-129-3p	52.11111	0.5581416	1122.407	3.678E-38	4357.519	3.678E-38	69.22222	0.01399127
mmu-miR-129-5p	2487.778	3.678E-38	1168.704	3.678E-38	2961.815	3.678E-38	569.3333	3.678E-38
mmu-miR-1-2-as	52.72222	0.4958616	54.59259	0.1452083	48.40741	0.5305763	53.77778	0.4653155
mmu-miR-130a	880.4445	3.678E-38	5266.444	3.678E-38	3121.481	3.678E-38	7388.444	3.678E-38
mmu-miR-130b	4316.111	3.678E-38	401.5555	3.678E-38	487.3704	3.678E-38	237	3.678E-38
mmu-miR-130b*	15329.72	3.678E-38	3118.222	3.678E-38	4347	3.678E-38	6271.667	3.678E-38
mmu-miR-132	3264.167	3.678E-38	4134.667	3.678E-38	4130.444	3.678E-38	5017.333	3.678E-38
mmu-miR-133a	1375.722	3.678E-38	652.037	3.678E-38	528.2963	3.678E-38	1696.222	3.678E-38
mmu-miR-133a*	416.0555	3.678E-38	151.5556	3.678E-38	85.48148	5.37397E-13	789	3.678E-38
mmu-miR-133b	104.6667	5.31423E-13	114.2963	3.74391E-33	1130.037	3.678E-38	289.2222	3.678E-38
mmu-miR-134	92.55556	3.7981E-07	178.5556	3.678E-38	168.1852	3.678E-38	90.22222	2.02224E-07
mmu-miR-135a	141.4444	1.03172E-21	3896.704	3.678E-38	5357.222	3.678E-38	4575	3.678E-38
mmu-miR-135a*	47.22222	0.7520519	46.74074	0.7196814	44.92593	0.782505	5702.111	3.678E-38
mmu-miR-135b	746.3333	3.678E-38	226.7037	3.678E-38	2341.111	3.678E-38	380.3333	3.678E-38
mmu-miR-136	50.22222	0.6190516	399.9259	3.678E-38	206.5926	3.678E-38	52.33333	0.543927

mmu-miR-136*	47.11111	0.7744733	235.9259	3.678E-38	117.5185	3.678E-38	47.55556	0.7773321
mmu-miR-137	67.55556	0.02442143	1782.481	3.678E-38	2837.852	3.678E-38	145.4444	9.23057E-37
mmu-miR-138	1219.667	3.678E-38	74.44444	4.73482E-06	495.3333	3.678E-38	141.4444	8.16907E-34
mmu-miR-138*	52.61111	0.5030171	54.85185	0.1474167	393.7778	3.678E-38	148.4444	3.678E-38
mmu-miR-139-3p	99.11111	7.31051E-10	221.6667	3.678E-38	173.9259	3.678E-38	135.1111	2.06364E-29
mmu-miR-139-5p	48.16667	0.7201433	6135.222	3.678E-38	4455.778	3.678E-38	52.66667	0.5258263
mmu-miR-140	3933.222	3.678E-38	713.5555	3.678E-38	1190.481	3.678E-38	4161.889	3.678E-38
mmu-miR-140*	5435.556	3.678E-38	1581.963	3.678E-38	2490.741	3.678E-38	69	0.01511116
mmu-miR-141	53.72222	0.4892944	9311	3.678E-38	9787.223	3.678E-38	13254.56	3.678E-38
mmu-miR-141*	547.5	3.678E-38	1825.407	3.678E-38	1010.593	3.678E-38	1832.111	3.678E-38
mmu-miR-142-3p	11146.06	3.678E-38	4396.963	3.678E-38	6815.852	3.678E-38	10935.11	3.678E-38
mmu-miR-142-5p	6888.333	3.678E-38	1750.074	3.678E-38	2242.556	3.678E-38	6120.444	3.678E-38
mmu-miR-143	1605	3.678E-38	13043.81	3.678E-38	13694.85	3.678E-38	19939.55	3.678E-38
mmu-miR-144	56.94444	0.2475307	50.14815	0.4766946	48.74074	0.5176281	48.22222	0.7492445
mmu-miR-144:9.1	919.6667	3.678E-38	1085.333	3.678E-38	114.9259	3.678E-38	1060.222	3.678E-38
mmu-miR-145	2122.611	3.678E-38	12132.67	3.678E-38	12190.52	3.678E-38	13034.78	3.678E-38
mmu-miR-145*	535.6667	3.678E-38	569.1111	3.678E-38	48.37037	0.5626761	45.88889	0.8391483
mmu-miR-146a	7577.333	3.678E-38	13874.3	3.678E-38	14227.3	3.678E-38	9575.889	3.678E-38
mmu-miR-146b	2231.389	3.678E-38	4605.629	3.678E-38	7200.074	3.678E-38	60.66667	0.1518772
mmu-miR-146b*	316.6667	3.678E-38	210.4074	3.678E-38	80.25926	6.24333E-10	3297.889	3.678E-38
mmu-miR-147	61.38889	0.1398906	400.4074	3.678E-38	682.5185	3.678E-38	55.66667	0.3649845
mmu-miR-148a	4525.167	3.678E-38	10537.37	3.678E-38	10560.04	3.678E-38	9163.444	3.678E-38
mmu-miR-148a*	92.44444	3.84888E-06	63.55556	0.002991569	54.88889	0.1242147	93.33334	1.98846E-08
mmu-miR-148b	1881.111	3.678E-38	1803.222	3.678E-38	1303.889	3.678E-38	71.22222	0.00674002
mmu-miR-149	3903.556	3.678E-38	7001.815	3.678E-38	8115.667	3.678E-38	6891.556	3.678E-38
mmu-miR-150	14713.67	3.678E-38	5166.148	3.678E-38	5286.778	3.678E-38	7292.667	3.678E-38
mmu-miR-150*	66.77778	0.02228466	64.2963	0.00492296	54.7037	0.1042653	54	0.4532646
mmu-miR-151-3p	1556.611	3.678E-38	5800.815	3.678E-38	5331.296	3.678E-38	3228.556	3.678E-38
mmu-miR-151-5p	8051.278	3.678E-38	11761.63	3.678E-38	11688.93	3.678E-38	5642.778	3.678E-38
mmu-miR-152	47.44444	0.7519135	9303.926	3.678E-38	8480.259	3.678E-38	7952.556	3.678E-38
mmu-miR-153	3493.389	3.678E-38	3127.333	3.678E-38	4064.667	3.678E-38	6085.333	3.678E-38
mmu-miR-154	56.88889	0.3156023	68.22222	0.000253592	51.74074	0.2746088	119.3333	7.5812E-20
mmu-miR-154*	50.33333	0.603534	47.96296	0.6342736	202	3.678E-38	54.66667	0.4174125
mmu-miR-155	12192.61	3.678E-38	8765.223	3.678E-38	14399.63	3.678E-38	8381.556	3.678E-38
mmu-miR-15a	3113.444	3.678E-38	1324.667	3.678E-38	1860.444	3.678E-38	3721.444	3.678E-38
mmu-miR-15a*	2790.611	3.678E-38	334.8148	3.678E-38	78.85185	2.66906E-09	893.7778	3.678E-38
mmu-miR-15b	19939.55	3.678E-38	14309.7	3.678E-38	15278	3.678E-38	17983.33	3.678E-38
mmu-miR-15b*	13204.94	3.678E-38	3602.815	3.678E-38	6977.667	3.678E-38	4055	3.678E-38
mmu-miR-16	16635	3.678E-38	12306.56	3.678E-38	13093.59	3.678E-38	14186.67	3.678E-38
mmu-miR-16*	608.7778	3.678E-38	178.7407	3.678E-38	165.4815	3.678E-38	1046.222	3.678E-38
mmu-miR-17	17942.22	3.678E-38	9545.186	3.678E-38	10842.07	3.678E-38	2163.444	3.678E-38
mmu-miR-17*	3699.111	3.678E-38	2622.889	3.678E-38	2818.667	3.678E-38	706.3333	3.678E-38
mmu-miR-181a	19060.22	3.678E-38	11873.48	3.678E-38	9507.63	3.678E-38	11588.89	3.678E-38
mmu-miR-181a-1*	4674.5	3.678E-38	482.4074	3.678E-38	302.7778	3.678E-38	47.88889	0.7635178
mmu-miR-181a-2*	569.2778	3.678E-38	114.4444	4.62098E-33	61.81482	0.005654503	255.7778	3.678E-38
mmu-miR-181b	25354.78	3.678E-38	12285.81	3.678E-38	8871.963	3.678E-38	10130	3.678E-38
mmu-miR-181c	3198.611	3.678E-38	818.8148	3.678E-38	127.4074	3.678E-38	51.88889	0.5679138
mmu-miR-181d	876.1667	3.678E-38	91.74074	2.14827E-22	52.14815	0.2190671	45.88889	0.8391483
mmu-miR-182	315.4445	3.678E-38	10488.44	3.678E-38	8300.296	3.678E-38	5350.111	3.678E-38
mmu-miR-183	1692.611	3.678E-38	16894.96	3.678E-38	15920.26	3.678E-38	5890.444	3.678E-38
mmu-miR-183*	81	9.41335E-05	2188.444	3.678E-38	2450.259	3.678E-38	111.7778	5.66709E-16
mmu-miR-184	55.77778	0.3016286	6509.926	3.678E-38	9268.963	3.678E-38	3835.111	3.678E-38
mmu-miR-185	817.0555	3.678E-38	2063.296	3.678E-38	3279.481	3.678E-38	2990.111	3.678E-38
mmu-miR-186	1023.889	3.678E-38	632.4815	3.678E-38	652.7407	3.678E-38	75.11111	0.001340053
mmu-miR-186*	202	3.678E-38	71.48148	2.02203E-05	59.51852	0.0208136	120.8889	1.06045E-20
mmu-miR-187	73.72222	0.003354827	381.2222	3.678E-38	789.3333	3.678E-38	127.3333	1.9041E-24
mmu-miR-188-3p	533.1667	3.678E-38	49.77778	0.5172955	53.88889	0.1728449	51.44444	0.5916526
mmu-miR-188-5p	226	3.678E-38	783.7037	3.678E-38	225.8148	3.678E-38	1803.889	3.678E-38
mmu-miR-18a	5813.333	3.678E-38	4105.889	3.678E-38	5760.037	3.678E-38	1744	3.678E-38
mmu-miR-18a*	5125.611	3.678E-38	93.33334	4.02196E-15	1002.185	3.678E-38	100.4444	5.12188E-11
mmu-miR-18b	1374.222	3.678E-38	54.88889	0.1797159	107.4074	2.79392E-31	75.88889	0.000940178
mmu-miR-190	107.6667	4.41966E-11	324.3333	3.678E-38	793.5555	3.678E-38	396.5555	3.678E-38
mmu-miR-190b	346.1667	3.678E-38	63.77778	0.004282647	541.8519	3.678E-38	151.6667	3.678E-38
mmu-miR-191	17332.39	3.678E-38	18989.52	3.678E-38	19942.37	3.678E-38	16903.89	3.678E-38
mmu-miR-191*	653.0555	3.678E-38	568	3.678E-38	524.5555	3.678E-38	579.8889	3.678E-38
mmu-miR-192	5321.944	3.678E-38	3925.63	3.678E-38	6372.629	3.678E-38	6019.556	3.678E-38
mmu-miR-193	5176.833	3.678E-38	4248.889	3.678E-38	3153.296	3.678E-38	11059	3.678E-38
mmu-miR-193*	1365.333	3.678E-38	1067.593	3.678E-38	753.5926	3.678E-38	1201.444	3.678E-38
mmu-miR-193b	368.3333	3.678E-38	2761.63	3.678E-38	2354.296	3.678E-38	1581.444	3.678E-38
mmu-miR-194	5263.111	3.678E-38	2299.074	3.678E-38	2461.185	3.678E-38	7444.889	3.678E-38
mmu-miR-195	5990.667	3.678E-38	7830.111	3.678E-38	4526.259	3.678E-38	5530	3.678E-38
mmu-miR-196a	8628.389	3.678E-38	8210.111	3.678E-38	11233.07	3.678E-38	6411.444	3.678E-38
mmu-miR-196a*	1212.333	3.678E-38	700.2593	3.678E-38	229	3.678E-38	635.2222	3.678E-38
mmu-miR-196b	6543.389	3.678E-38	2362	3.678E-38	2564.259	3.678E-38	7545.111	3.678E-38
mmu-miR-197	102.8889	8.24903E-11	1099.037	3.678E-38	515.8519	3.678E-38	81.66666	4.85026E-05
mmu-miR-199a-3p,mmu-m	6102.667	3.678E-38	6341.926	3.678E-38	5323.629	3.678E-38	5749.778	3.678E-38
mmu-miR-199a-3p:9.1	5774.056	3.678E-38	3130.593	3.678E-38	3748.333	3.678E-38	4330.667	3.678E-38
mmu-miR-199a-5p	7749.556	3.678E-38	4499.852	3.678E-38	2110.481	3.678E-38	12521	3.678E-38
mmu-miR-199b*	83.44444	0.000439276	176.5185	3.678E-38	118.6667	3.678E-38	80.77778	7.95199E-05
mmu-miR-19a	1628.167	3.678E-38	683.3704	3.678E-38	1049.444	3.678E-38	5412.444	3.678E-38

mmu-miR-19a*	71	0.006435453	70.74074	6.3817E-05	72.40741	8.93397E-06	84.33334	1.01164E-05
mmu-miR-19b	4085.111	3.678E-38	794.9259	3.678E-38	1971.222	3.678E-38	6472	3.678E-38
mmu-miR-200a	60.77778	0.184484	16724.55	3.678E-38	15487.81	3.678E-38	13521.56	3.678E-38
mmu-miR-200a*	66.55556	0.01616389	1404.667	3.678E-38	556.5185	3.678E-38	50.55556	0.638055
mmu-miR-200b	2900.833	3.678E-38	25354.78	3.678E-38	22804.07	3.678E-38	25354.78	3.678E-38
mmu-miR-200b*	319.5	3.678E-38	3071.185	3.678E-38	1928.556	3.678E-38	3806.333	3.678E-38
mmu-miR-200c	5594.333	3.678E-38	19003.3	3.678E-38	19009.59	3.678E-38	17283.67	3.678E-38
mmu-miR-200c*	138.0556	4.90307E-21	523.8889	3.678E-38	283.4815	3.678E-38	234.7778	3.678E-38
mmu-miR-201	232.8333	3.678E-38	68.59259	0.000277873	421	3.678E-38	244	3.678E-38
mmu-miR-202-3p	49.61111	0.660755	2099.037	3.678E-38	4735.556	3.678E-38	49.33333	0.6985662
mmu-miR-202-5p	51.44444	0.5408093	1081.148	3.678E-38	6545.333	3.678E-38	45.44444	0.8535306
mmu-miR-203	240.5556	3.678E-38	19062.89	3.678E-38	20653.52	3.678E-38	13455.67	3.678E-38
mmu-miR-203*	102.6667	8.42707E-08	128.1111	3.678E-38	626.1852	3.678E-38	122.3333	1.64016E-21
mmu-miR-204	50.66667	0.6026601	5905.852	3.678E-38	5199.407	3.678E-38	3573.778	3.678E-38
mmu-miR-205	1152.222	3.678E-38	16194.11	3.678E-38	14342.81	3.678E-38	14363.44	3.678E-38
mmu-miR-206	44.88889	0.8389058	5230.371	3.678E-38	6251.296	3.678E-38	5963.333	3.678E-38
mmu-miR-207	943.7222	3.678E-38	358.4074	3.678E-38	177.8519	3.678E-38	49.33333	0.6985662
mmu-miR-208a	378.8333	3.678E-38	137.4444	3.678E-38	71.62963	6.36584E-06	323.4445	3.678E-38
mmu-miR-208b	237.7778	3.678E-38	100.6667	5.88632E-20	58.33333	0.02753858	125.4444	2.58302E-23
mmu-miR-20a	12465.83	3.678E-38	6368.852	3.678E-38	9571.889	3.678E-38	9276.667	3.678E-38
mmu-miR-20a*	15209.78	3.678E-38	10571.7	3.678E-38	8850.889	3.678E-38	14558.78	3.678E-38
mmu-miR-20b	13058.61	3.678E-38	2165.593	3.678E-38	3344.37	3.678E-38	6517.222	3.678E-38
mmu-miR-20b*	2172.222	3.678E-38	47.40741	0.6645898	48	0.5508792	50	0.6661043
mmu-miR-21	18819	3.678E-38	19522.89	3.678E-38	19261.93	3.678E-38	20722.45	3.678E-38
mmu-miR-21*	266.2778	3.678E-38	102.6296	3.30696E-32	339.4815	3.678E-38	101.3333	2.27847E-11
mmu-miR-210	4472.444	3.678E-38	2342.963	3.678E-38	231.2222	3.678E-38	4428.556	3.678E-38
mmu-miR-211	548.4445	3.678E-38	259.6296	3.678E-38	497.3704	3.678E-38	1030.556	3.678E-38
mmu-miR-212	7115.056	3.678E-38	5894.852	3.678E-38	4418.926	3.678E-38	5453.889	3.678E-38
mmu-miR-214	1484.778	3.678E-38	4596.852	3.678E-38	3971.741	3.678E-38	6208.667	3.678E-38
mmu-miR-214*	50.66667	0.5687774	50.77778	0.3913274	78.55556	1.21918E-13	51.44444	0.5916526
mmu-miR-215	567.4445	3.678E-38	10318.41	3.678E-38	16556.19	3.678E-38	9051.667	3.678E-38
mmu-miR-216a	176	3.678E-38	80.59259	4.58752E-09	57.81482	0.03969681	128.5556	3.40147E-25
mmu-miR-216b	216.7222	3.678E-38	231.7037	3.678E-38	266.3704	3.678E-38	842.3333	3.678E-38
mmu-miR-217	152.9444	1.22603E-33	813.2963	3.678E-38	3077.963	3.678E-38	77.44444	0.000448384
mmu-miR-218	51.11111	0.5709645	8287.704	3.678E-38	6635.296	3.678E-38	3595.222	3.678E-38
mmu-miR-218-1*	92.61111	4.34926E-07	52.85185	0.2610765	117.5185	1.77173E-33	74.22222	0.001983539
mmu-miR-218-2*	227.7222	3.678E-38	98.14815	6.74158E-20	304.1111	3.678E-38	268.2222	3.678E-38
mmu-miR-219	374.3333	3.678E-38	132.6667	3.678E-38	242	3.678E-38	508.1111	3.678E-38
mmu-miR-22	4996.333	3.678E-38	12570.85	3.678E-38	13976.3	3.678E-38	18409.45	3.678E-38
mmu-miR-22*	333.7778	3.678E-38	1716.593	3.678E-38	2576.963	3.678E-38	3284.333	3.678E-38
mmu-miR-220	47.44444	0.7416079	46.18518	0.748144	75.11111	4.23463E-06	42.88889	0.9193798
mmu-miR-221	4189.389	3.678E-38	3695.444	3.678E-38	2626	3.678E-38	4901.111	3.678E-38
mmu-miR-222	2617.889	3.678E-38	3981.852	3.678E-38	4586.259	3.678E-38	1978.667	3.678E-38
mmu-miR-223	5036.222	3.678E-38	3528.593	3.678E-38	2111.963	3.678E-38	1918.444	3.678E-38
mmu-miR-224	43.33333	0.886642	1347.556	3.678E-38	2118	3.678E-38	45.44444	0.8535306
mmu-miR-23a	4856.5	3.678E-38	10342.37	3.678E-38	8587.186	3.678E-38	4705.222	3.678E-38
mmu-miR-23b	17825.22	3.678E-38	19866.63	3.678E-38	18875.15	3.678E-38	19552.33	3.678E-38
mmu-miR-24	13067.61	3.678E-38	17867.55	3.678E-38	17231.41	3.678E-38	22335	3.678E-38
mmu-miR-24-1*	49.5	0.6555128	1177.037	3.678E-38	451.6667	3.678E-38	189.7778	3.678E-38
mmu-miR-24-2*	967.7222	3.678E-38	1970.037	3.678E-38	970.5555	3.678E-38	2712.333	3.678E-38
mmu-miR-25	18671.33	3.678E-38	11898.7	3.678E-38	11391.7	3.678E-38	13342.78	3.678E-38
mmu-miR-26a	17285.5	3.678E-38	17549.48	3.678E-38	16043.52	3.678E-38	18937.89	3.678E-38
mmu-miR-26b	12887.78	3.678E-38	12410.04	3.678E-38	11806.59	3.678E-38	12833.67	3.678E-38
mmu-miR-26b*	3179.944	3.678E-38	1499.741	3.678E-38	243.0741	3.678E-38	44.11111	0.8913791
mmu-miR-27a	5187.944	3.678E-38	10417.59	3.678E-38	9895.814	3.678E-38	14855.78	3.678E-38
mmu-miR-27a*	103.2778	5.82088E-11	111.8889	2.28051E-30	189.6296	3.678E-38	58.22222	0.243721
mmu-miR-27b	5790.333	3.678E-38	12552	3.678E-38	10976.89	3.678E-38	11883.33	3.678E-38
mmu-miR-27b*	1123.889	3.678E-38	3015.407	3.678E-38	2139.519	3.678E-38	54.77778	0.4114952
mmu-miR-28	4855.111	3.678E-38	8855.926	3.678E-38	8470	3.678E-38	2004.778	3.678E-38
mmu-miR-28*	5009.611	3.678E-38	2323.926	3.678E-38	2710.037	3.678E-38	54.77778	0.4114952
mmu-miR-290-3p	51.77778	0.5386307	215.2963	3.678E-38	213.4444	3.678E-38	48.22222	0.7492445
mmu-miR-290-5p	152.7222	3.678E-38	100.3333	3.70751E-21	854	3.678E-38	407.8889	3.678E-38
mmu-miR-291a-3p	268.2778	3.678E-38	108.4444	4.94149E-27	189.5926	3.678E-38	258.3333	3.678E-38
mmu-miR-291a-5p	52.05556	0.5272072	366.4074	3.678E-38	1574.296	3.678E-38	54.55556	0.4233487
mmu-miR-291b-3p	138.0556	5.35432E-23	68.07407	0.000248035	282.6667	3.678E-38	54.11111	0.4472545
mmu-miR-291b-5p	63.88889	0.1167815	755.6296	3.678E-38	572.1481	3.678E-38	57.77778	0.2631547
mmu-miR-292-3p	60.94444	0.1541654	55.85185	0.1246999	255.4444	3.678E-38	113.2222	1.11717E-16
mmu-miR-292-5p	46.61111	0.7878367	1176.259	3.678E-38	1583	3.678E-38	48.55556	0.7345276
mmu-miR-293	922.8889	3.678E-38	732.2593	3.678E-38	1560.111	3.678E-38	1084.111	3.678E-38
mmu-miR-293*	2875.111	3.678E-38	127.9259	3.678E-38	284.8148	3.678E-38	723.5555	3.678E-38
mmu-miR-294	93.66666	1.06305E-08	576.4445	3.678E-38	2557	3.678E-38	71	0.007334237
mmu-miR-294*	74.61111	0.000918722	844.5185	3.678E-38	691.0741	3.678E-38	73.22222	0.003033644
mmu-miR-295	46.22222	0.7993115	1066.481	3.678E-38	2501.556	3.678E-38	55.11111	0.3938685
mmu-miR-295*	688.2778	3.678E-38	1018.296	3.678E-38	332.963	3.678E-38	782.5555	3.678E-38
mmu-miR-296-3p	1738.333	3.678E-38	3396.593	3.678E-38	1024.185	3.678E-38	55.55556	0.3707062
mmu-miR-296-5p	4486.444	3.678E-38	761.7778	3.678E-38	969.0741	3.678E-38	72.22222	0.004560621
mmu-miR-297a	1721.056	3.678E-38	853.2593	3.678E-38	789.1111	3.678E-38	1680.778	3.678E-38
mmu-miR-297b-5p	600.8889	3.678E-38	60.44444	0.02086512	57.74074	0.03863371	138	2.22099E-31
mmu-miR-297c	68.88889	0.01042124	1801.852	3.678E-38	293.4445	3.678E-38	4376.444	3.678E-38
mmu-miR-297c*,mmu-miR-	7688.722	3.678E-38	2338.704	3.678E-38	1990.111	3.678E-38	7220.889	3.678E-38

mmu-miR-298	5096.667	3.678E-38	702.3333	3.678E-38	2179.963	3.678E-38	52.77778	0.519779
mmu-miR-299	273.2222	3.678E-38	245.6296	3.678E-38	82.62963	3.28722E-11	902.1111	3.678E-38
mmu-miR-299*	181.5	3.678E-38	81.66666	7.80219E-10	108.8889	6.04734E-36	83.44444	1.73003E-05
mmu-miR-29a	4139.5	3.678E-38	8278.223	3.678E-38	7547.593	3.678E-38	12661.56	3.678E-38
mmu-miR-29a*	1022.333	3.678E-38	1376.037	3.678E-38	1112.333	3.678E-38	733.5555	3.678E-38
mmu-miR-29b	4943.278	3.678E-38	8861.296	3.678E-38	6769.296	3.678E-38	15303	3.678E-38
mmu-miR-29b*	43.44444	0.8818891	478.1482	3.678E-38	314.1111	3.678E-38	45.22222	0.8603877
mmu-miR-29c	85.44444	7.16094E-06	5006.556	3.678E-38	3970.63	3.678E-38	9912.111	3.678E-38
mmu-miR-29c*	72.83334	0.004048863	66.44444	0.000838505	85.25926	7.75891E-13	69.11111	0.01454194
mmu-miR-300	50.38889	0.5925888	60.11111	0.03583225	52.66667	0.2431705	52.11111	0.555946
mmu-miR-300*	895.2222	3.678E-38	1084.37	3.678E-38	61	0.003930886	88	9.52464E-07
mmu-miR-301a	1040.944	3.678E-38	178.5556	3.678E-38	349.5185	3.678E-38	143.6667	1.95388E-35
mmu-miR-301b	47.72222	0.7489274	46.96296	0.6948089	47.11111	0.639396	47.55556	0.7773321
mmu-miR-302a	73.38889	0.002227926	64.96296	0.00285836	208.1111	3.678E-38	131.6667	3.74492E-27
mmu-miR-302a*	52	0.4999629	49.62963	0.4953757	49.51852	0.4583347	47.55556	0.7773321
mmu-miR-302b	1669.056	3.678E-38	528.2222	3.678E-38	6855.815	3.678E-38	692.7778	3.678E-38
mmu-miR-302b*	47.66667	0.7527938	47.07407	0.6963459	47.33333	0.6248093	57.88889	0.2582231
mmu-miR-302c	392	3.678E-38	120.037	5.32281E-37	65.7037	0.000442275	698.7778	3.678E-38
mmu-miR-302c*	55.38889	0.3320003	1006.407	3.678E-38	91.55556	5.95938E-19	3099.333	3.678E-38
mmu-miR-302d	152.8333	6.83175E-37	598.037	3.678E-38	737.7037	3.678E-38	187.4444	3.678E-38
mmu-miR-30a	2252	3.678E-38	6508	3.678E-38	7024.407	3.678E-38	8938.777	3.678E-38
mmu-miR-30a*	193.3889	3.678E-38	2037.296	3.678E-38	4475.074	3.678E-38	3211.667	3.678E-38
mmu-miR-30b	10296.28	3.678E-38	10447.63	3.678E-38	11105.15	3.678E-38	10288.56	3.678E-38
mmu-miR-30b*	79.55556	0.000305108	219.4444	3.678E-38	228.4444	3.678E-38	88.77778	5.59359E-07
mmu-miR-30c	12035	3.678E-38	13156.59	3.678E-38	14089	3.678E-38	14713.67	3.678E-38
mmu-miR-30c-1*	675.2778	3.678E-38	226.8889	3.678E-38	208.3704	3.678E-38	316.5555	3.678E-38
mmu-miR-30c-2*	981.5555	3.678E-38	371.0741	3.678E-38	120.2222	3.678E-38	1236.111	3.678E-38
mmu-miR-30d	7435.056	3.678E-38	12846.63	3.678E-38	10708.67	3.678E-38	16001.11	3.678E-38
mmu-miR-30e	4043.944	3.678E-38	6457.296	3.678E-38	3893.963	3.678E-38	7131.889	3.678E-38
mmu-miR-30e*	5112.278	3.678E-38	5927.222	3.678E-38	5323.889	3.678E-38	4517.556	3.678E-38
mmu-miR-31	49.77778	0.6303791	2898.741	3.678E-38	2418.407	3.678E-38	49.77778	0.6770796
mmu-miR-31*	83.94444	3.29695E-05	540.4074	3.678E-38	434.037	3.678E-38	56.11111	0.342408
mmu-miR-32	56.83333	0.3468821	60.37037	0.01306815	93.85185	1.80911E-24	73.44444	0.002764431
mmu-miR-320	6680.056	3.678E-38	10502.59	3.678E-38	8911.259	3.678E-38	4242.889	3.678E-38
mmu-miR-322	539.0555	3.678E-38	444.1852	3.678E-38	1500.185	3.678E-38	2694.778	3.678E-38
mmu-miR-322*	51.72222	0.5268273	284.8518	3.678E-38	1002.407	3.678E-38	55.88889	0.3536319
mmu-miR-323-3p	328.6667	3.678E-38	128.1111	3.678E-38	69.7037	2.55958E-05	482.8889	3.678E-38
mmu-miR-323-5p	369.2222	3.678E-38	125.7037	3.678E-38	96.03704	3.91432E-29	252.8889	3.678E-38
mmu-miR-324-3p	4477.111	3.678E-38	3610.704	3.678E-38	4412.778	3.678E-38	4626.667	3.678E-38
mmu-miR-324-5p	2174.833	3.678E-38	1143.259	3.678E-38	704.963	3.678E-38	2237.778	3.678E-38
mmu-miR-325	178.3889	3.678E-38	98.18519	2.75648E-19	81.59259	1.515E-11	246.7778	3.678E-38
mmu-miR-325*	61.22222	0.2157767	48.37037	0.5863166	46.55556	0.6825427	53.33333	0.4895019
mmu-miR-326	3352.667	3.678E-38	3035.222	3.678E-38	2553.519	3.678E-38	1442.444	3.678E-38
mmu-miR-327	1068.889	3.678E-38	159	3.678E-38	100.5926	2.25793E-24	540.2222	3.678E-38
mmu-miR-328	4037.722	3.678E-38	6687.148	3.678E-38	6146.963	3.678E-38	6980.333	3.678E-38
mmu-miR-329	252.8889	3.678E-38	103.1852	1.27222E-22	200.1852	3.678E-38	465.3333	3.678E-38
mmu-miR-33	1401.278	3.678E-38	278.7037	3.678E-38	238.5185	3.678E-38	176.3333	3.678E-38
mmu-miR-33*	47.44444	0.7467883	191.7778	3.678E-38	649.3333	3.678E-38	56.11111	0.342408
mmu-miR-330	122.1111	1.96406E-20	3281.111	3.678E-38	1073.296	3.678E-38	476.5555	3.678E-38
mmu-miR-330*	997.5	3.678E-38	762.0741	3.678E-38	439.3333	3.678E-38	53.66667	0.4713536
mmu-miR-331-3p	2671.056	3.678E-38	3459.333	3.678E-38	3214.852	3.678E-38	58.44444	0.2343028
mmu-miR-331-5p	57.66667	0.2066234	50.22222	0.4585161	73.88889	5.42838E-10	58.66667	0.2250896
mmu-miR-335-3p	45.16667	0.825226	705.2222	3.678E-38	933.037	3.678E-38	52.22222	0.5499422
mmu-miR-335-5p	357.1111	3.678E-38	192.2222	3.678E-38	451.5185	3.678E-38	1591.889	3.678E-38
mmu-miR-337-3p	328.1667	3.678E-38	49	0.5578387	48.77778	0.5035037	46.88889	0.8035344
mmu-miR-337-5p	1088.556	3.678E-38	593.7037	3.678E-38	352.4074	3.678E-38	1476.778	3.678E-38
mmu-miR-338-3p	55.66667	0.3251066	189.2593	3.678E-38	95.40741	1.5821E-21	94.55556	7.61985E-09
mmu-miR-338-5p	493.6111	3.678E-38	290.7778	3.678E-38	262.8889	3.678E-38	914.5555	3.678E-38
mmu-miR-339-3p	1242	3.678E-38	1076.815	3.678E-38	249.5556	3.678E-38	101.7778	1.51147E-11
mmu-miR-339-5p	310	3.678E-38	790.7037	3.678E-38	641.8889	3.678E-38	2184	3.678E-38
mmu-miR-340-3p	3887.389	3.678E-38	1661.333	3.678E-38	1282.741	3.678E-38	2879	3.678E-38
mmu-miR-340-5p	1421.056	3.678E-38	2038.852	3.678E-38	1876.296	3.678E-38	2209	3.678E-38
mmu-miR-341	1104.667	3.678E-38	180	3.678E-38	442.963	3.678E-38	46.44444	0.8199165
mmu-miR-341:9.1	559.0555	3.678E-38	175.1481	3.678E-38	84.81481	7.55417E-13	797.2222	3.678E-38
mmu-miR-342-3p	7257.167	3.678E-38	8754.259	3.678E-38	7011.963	3.678E-38	8700.111	3.678E-38
mmu-miR-342-5p	5911.056	3.678E-38	6045.741	3.678E-38	3597.667	3.678E-38	2017.556	3.678E-38
mmu-miR-343	373.7778	3.678E-38	526.6296	3.678E-38	562	3.678E-38	49.11111	0.7090624
mmu-miR-344	8556.889	3.678E-38	3989.667	3.678E-38	3004.444	3.678E-38	9226.223	3.678E-38
mmu-miR-345-3p	739.8333	3.678E-38	646.7778	3.678E-38	700.4074	3.678E-38	777.4445	3.678E-38
mmu-miR-345-5p	1484.611	3.678E-38	898.3704	3.678E-38	473.5185	3.678E-38	163.4444	3.678E-38
mmu-miR-346	16293.56	3.678E-38	11507.78	3.678E-38	8738.444	3.678E-38	16296	3.678E-38
mmu-miR-34a	3409.056	3.678E-38	3450.037	3.678E-38	3640.333	3.678E-38	5493.222	3.678E-38
mmu-miR-34b-3p	241.1667	3.678E-38	6980.037	3.678E-38	5083.074	3.678E-38	107.7778	4.16288E-14
mmu-miR-34b-5p	3409.056	3.678E-38	373.3333	3.678E-38	707.1481	3.678E-38	342.5555	3.678E-38
mmu-miR-34c	51.94444	0.5253707	1436.704	3.678E-38	153.2963	3.678E-38	2043.444	3.678E-38
mmu-miR-34c*	93.22222	1.01046E-08	7583.371	3.678E-38	5327.593	3.678E-38	4924.333	3.678E-38
mmu-miR-350	4355.167	3.678E-38	3663.519	3.678E-38	2860.148	3.678E-38	169.3333	3.678E-38
mmu-miR-351	4580.222	3.678E-38	641.2593	3.678E-38	994.6296	3.678E-38	6932.556	3.678E-38
mmu-miR-361	6103.722	3.678E-38	4528.185	3.678E-38	5523.259	3.678E-38	1011.111	3.678E-38
mmu-miR-362-3p	1948.278	3.678E-38	149.2593	3.678E-38	3164.185	3.678E-38	2483.444	3.678E-38

mmu-miR-362-5p	95.61111	3.58613E-06	266.5185	3.678E-38	211.8519	3.678E-38	210.1111	3.678E-38
mmu-miR-363	1270.333	3.678E-38	68.40741	1.18208E-05	50.51852	0.3414095	51.66667	0.5798195
mmu-miR-365	448.7222	3.678E-38	2771.815	3.678E-38	1584.37	3.678E-38	2670.444	3.678E-38
mmu-miR-367	47.77778	0.7271334	47.7037	0.6408591	51.81482	0.2669974	49.11111	0.7090624
mmu-miR-369-3p	172.7778	3.678E-38	100.963	1.69878E-23	219.2222	3.678E-38	363.8889	3.678E-38
mmu-miR-369-5p	97.88889	2.85095E-07	57.62963	0.06719078	77.40741	5.80984E-12	175.5556	3.678E-38
mmu-miR-370	44.61111	0.8599087	44.2963	0.8574236	43.62963	0.8560344	49	0.7142457
mmu-miR-374	10672.22	3.678E-38	5598.889	3.678E-38	7032	3.678E-38	6170	3.678E-38
mmu-miR-374*	55.33333	0.3957507	120.8889	3.678E-38	46.48148	0.6778152	76.44444	0.000725226
mmu-miR-375	466.6667	3.678E-38	14770.85	3.678E-38	12872.78	3.678E-38	9434	3.678E-38
mmu-miR-376a	344.6667	3.678E-38	132.5185	3.678E-38	1346.111	3.678E-38	376.8889	3.678E-38
mmu-miR-376a*	60.55556	0.136874	356.4074	3.678E-38	52.40741	0.2397387	154.5556	3.678E-38
mmu-miR-376b	686.5	3.678E-38	380.2222	3.678E-38	530.7037	3.678E-38	561.8889	3.678E-38
mmu-miR-376b*	100.9444	5.54509E-09	2353.63	3.678E-38	880.4815	3.678E-38	4810	3.678E-38
mmu-miR-376c	1574.167	3.678E-38	232.5185	3.678E-38	206.8148	3.678E-38	46.77778	0.8077124
mmu-miR-376c*	399.6111	3.678E-38	128.8889	3.678E-38	99.40741	2.37971E-29	682.2222	3.678E-38
mmu-miR-377	48.61111	0.6977588	47.66667	0.6523899	75.40741	1.22703E-10	48.22222	0.7492445
mmu-miR-378	5386	3.678E-38	5932.296	3.678E-38	5970.629	3.678E-38	2324	3.678E-38
mmu-miR-378*	2014.833	3.678E-38	3071.815	3.678E-38	1667.926	3.678E-38	92.88889	2.79949E-08
mmu-miR-379	207.5	3.678E-38	417.8889	3.678E-38	850.2963	3.678E-38	220.2222	3.678E-38
mmu-miR-380-3p	46.22222	0.7864215	435.3704	3.678E-38	44.74074	0.7976072	3116.778	3.678E-38
mmu-miR-380-5p	46.77778	0.792152	47.62963	0.662028	46.48148	0.6834381	56.55556	0.3203811
mmu-miR-381	46.94444	0.7843611	43.44444	0.8933723	52.33333	0.1526066	43.66667	0.9022759
mmu-miR-382	149.3333	2.88002E-33	98.74074	1.55274E-19	503.4074	3.678E-38	275.6667	3.678E-38
mmu-miR-382*	79.27778	0.000129717	111.2963	3.44006E-31	139.0741	3.678E-38	593.2222	3.678E-38
mmu-miR-383	1729.556	3.678E-38	1610.815	3.678E-38	388.2592	3.678E-38	56.88889	0.3042614
mmu-miR-384-3p	60.05556	0.1655545	80.66666	1.19058E-12	53.77778	0.1646605	67	0.02912149
mmu-miR-384-5p	828.6111	3.678E-38	142.1481	3.678E-38	249	3.678E-38	1604.444	3.678E-38
mmu-miR-409-3p	46.44444	0.777756	43.77778	0.870882	102.1852	3.48639E-21	46.11111	0.8316225
mmu-miR-409-5p	271.1111	3.678E-38	201.4444	3.678E-38	452.6667	3.678E-38	769.4445	3.678E-38
mmu-miR-410	74.33334	0.00087861	58.74074	0.04960597	52.40741	0.2464341	56.55556	0.3203811
mmu-miR-411	67.94444	0.01366788	161.2593	3.678E-38	107.6667	3.24415E-35	4979.333	3.678E-38
mmu-miR-411*	47.05556	0.7493452	45.48148	0.7953746	45	0.7818189	49	0.7142457
mmu-miR-411*:9.1	1985.778	3.678E-38	572.2593	3.678E-38	248.2222	3.678E-38	2300.778	3.678E-38
mmu-miR-412	57.83333	0.2741613	60	0.03033474	53.62963	0.1665652	266.2222	3.678E-38
mmu-miR-412:9.1	49.11111	0.6863651	47.81482	0.6506492	47	0.6531579	57.22222	0.2885132
mmu-miR-421	3179.889	3.678E-38	2333.074	3.678E-38	2859.111	3.678E-38	607	3.678E-38
mmu-miR-423-3p	4707.889	3.678E-38	2560.37	3.678E-38	2646.667	3.678E-38	958.2222	3.678E-38
mmu-miR-423-5p	3091.222	3.678E-38	1288.519	3.678E-38	941.3704	3.678E-38	44.88889	0.8702583
mmu-miR-425	9449.444	3.678E-38	9620.852	3.678E-38	14681.67	3.678E-38	8266.667	3.678E-38
mmu-miR-425*	96.44444	3.73626E-12	443.1111	3.678E-38	93.66666	1.1156E-23	72	0.00498151
mmu-miR-429	444.6111	3.678E-38	16859.67	3.678E-38	16008.89	3.678E-38	10619.22	3.678E-38
mmu-miR-431	50.72222	0.5841861	49.40741	0.5218261	46.74074	0.679152	49.11111	0.7090624
mmu-miR-431*	52.5	0.5332662	51.55556	0.3648357	60.03704	0.01249814	100.8889	3.42232E-11
mmu-miR-433	62.66667	0.09407564	111.6296	1.2657E-30	61.88889	0.004896403	57.33333	0.2833506
mmu-miR-433*	64.94444	0.07739285	58.40741	0.04280236	52.74074	0.2164311	107	9.27712E-14
mmu-miR-434-3p	59.33333	0.1601564	473.6667	3.678E-38	291.5926	3.678E-38	68.44444	0.01825292
mmu-miR-434-5p	1484.389	3.678E-38	1881.481	3.678E-38	1392.593	3.678E-38	10702.33	3.678E-38
mmu-miR-448	70.88889	0.01452939	2071.222	3.678E-38	49.66667	0.4422547	58.22222	0.243721
mmu-miR-449a	1572.722	3.678E-38	2143.407	3.678E-38	4088.593	3.678E-38	49	0.7142457
mmu-miR-449b	65.72222	0.05735474	251.1111	3.678E-38	226.5185	3.678E-38	70	0.01061655
mmu-miR-449c	78.61111	0.006127425	63.85185	0.01142048	59.07407	0.006456828	49	0.7142457
mmu-miR-450a-3p	51.77778	0.5666509	50.74074	0.4367064	83.7037	8.87302E-18	53.22222	0.4955581
mmu-miR-450a-5p	45.22222	0.818692	1099.037	3.678E-38	1135.556	3.678E-38	50	0.6661043
mmu-miR-450b-3p	96.11111	1.59197E-09	282.5185	3.678E-38	368.6667	3.678E-38	66.44444	0.03453605
mmu-miR-450b-5p	52.5	0.4998915	701.1111	3.678E-38	100.037	1.60639E-34	49.88889	0.6716105
mmu-miR-451	12159.22	3.678E-38	6478.444	3.678E-38	7928.889	3.678E-38	3767.111	3.678E-38
mmu-miR-452	51.33333	0.5657278	50.62963	0.4394315	69.07407	0.000113444	53.88889	0.4592853
mmu-miR-453	1734.444	3.678E-38	572.8519	3.678E-38	259.7778	3.678E-38	3917.667	3.678E-38
mmu-miR-455	4948.833	3.678E-38	8600.519	3.678E-38	11253.15	3.678E-38	1418.778	3.678E-38
mmu-miR-455*	372.2778	3.678E-38	686.9259	3.678E-38	558.2963	3.678E-38	340.1111	3.678E-38
mmu-miR-463	229.5556	3.678E-38	112.1852	1.79279E-29	67.22222	0.00020289	389.1111	3.678E-38
mmu-miR-463*	3195.111	3.678E-38	46.25926	0.7554158	262.963	3.678E-38	44.55556	0.8796347
mmu-miR-464	63.16667	0.1053177	54.14815	0.212082	49.40741	0.4466235	68.44444	0.01825292
mmu-miR-465a-3p,mmu-m	162	3.678E-38	206.4815	3.678E-38	1610.481	3.678E-38	186	3.678E-38
mmu-miR-465a-5p	291.2778	3.678E-38	71.37037	2.11282E-06	205.2593	3.678E-38	643.4445	3.678E-38
mmu-miR-465b-5p	55.27778	0.3708948	187.5926	3.678E-38	63.2963	0.000524708	52.33333	0.543927
mmu-miR-465c-5p	1098.056	3.678E-38	452.4445	3.678E-38	198.5556	3.678E-38	1321.667	3.678E-38
mmu-miR-466a-3p,mmu-m	13306.17	3.678E-38	6046.111	3.678E-38	6400.259	3.678E-38	5060	3.678E-38
mmu-miR-466a-5p	879.5	3.678E-38	366.1482	3.678E-38	132.1111	3.678E-38	937.5555	3.678E-38
mmu-miR-466b-5p	1636.333	3.678E-38	59.11111	0.03688905	68.11111	2.96208E-06	47.33333	0.7862802
mmu-miR-466c-5p	5811.667	3.678E-38	1208.444	3.678E-38	1021.63	3.678E-38	1185.667	3.678E-38
mmu-miR-466d-3p	10582.83	3.678E-38	2803.444	3.678E-38	5585.556	3.678E-38	1534.667	3.678E-38
mmu-miR-466d-5p	79.5	0.000762087	513.6667	3.678E-38	54.51852	0.09201875	47.11111	0.7950153
mmu-miR-466e-5p	1094.222	3.678E-38	113.8519	2.87138E-31	299.4445	3.678E-38	533.3333	3.678E-38
mmu-miR-466f	2339.667	3.678E-38	462.1482	3.678E-38	311.3333	3.678E-38	929.2222	3.678E-38
mmu-miR-466f-3p	12520.39	3.678E-38	8547.741	3.678E-38	8815.556	3.678E-38	8202	3.678E-38
mmu-miR-466f-5p	490.5	3.678E-38	444.2963	3.678E-38	90.25926	1.28842E-18	816.3333	3.678E-38
mmu-miR-466g	5628.111	3.678E-38	666.7037	3.678E-38	2624.333	3.678E-38	52.44444	0.5379017
mmu-miR-466h	4922.722	3.678E-38	557.5185	3.678E-38	660	3.678E-38	625.3333	3.678E-38

mmu-miR-466i	11133.83	3.678E-38	7444.148	3.678E-38	7724.296	3.678E-38	7822.889	3.678E-38
mmu-miR-466j	1574.222	3.678E-38	82.44444	2.24096E-10	65.81481	0.00044571	657	3.678E-38
mmu-miR-466k	871.4445	3.678E-38	454.8889	3.678E-38	207.8148	3.678E-38	43.11111	0.9147417
mmu-miR-466l	102.7222	1.14701E-11	88.22222	4.31059E-15	58.18518	0.03339455	197	3.678E-38
mmu-miR-467a	4247.944	3.678E-38	1738.556	3.678E-38	1977.63	3.678E-38	1143.667	3.678E-38
mmu-miR-467a*,mmu-miR-	8447.556	3.678E-38	1201.63	3.678E-38	4391.852	3.678E-38	6582.444	3.678E-38
mmu-miR-467b	209.6667	3.678E-38	52.62963	0.2646001	52.62963	0.2175836	207	3.678E-38
mmu-miR-467b*	47.27778	0.7481795	46.14815	0.7555484	46.55556	0.6555863	45.66667	0.8464509
mmu-miR-467c	6145.611	3.678E-38	1558.889	3.678E-38	1343.556	3.678E-38	991.4445	3.678E-38
mmu-miR-467d	71	0.01175478	55.22222	0.1554333	594.1111	3.678E-38	115.3333	9.7135E-18
mmu-miR-467e	4780.222	3.678E-38	1557.63	3.678E-38	4126.963	3.678E-38	57.55556	0.2731601
mmu-miR-467e*	580.7222	3.678E-38	56.66667	0.08886591	245.7407	3.678E-38	98.55556	2.73005E-10
mmu-miR-467f	4519.889	3.678E-38	4008.63	3.678E-38	4050.185	3.678E-38	1553.556	3.678E-38
mmu-miR-467g	43.83333	0.8770495	44.85185	0.828762	45.07407	0.7854547	51.22222	0.6034028
mmu-miR-467h	1970.056	3.678E-38	506.963	3.678E-38	95.2963	7.50891E-20	664.8889	3.678E-38
mmu-miR-468	80.88889	0.000328274	75	5.60641E-08	54.7037	0.1165864	310.1111	3.678E-38
mmu-miR-469	1486.278	3.678E-38	1693.741	3.678E-38	55.11111	0.1103173	57.33333	0.2833506
mmu-miR-470	114.5556	4.81488E-14	55.07407	0.1555658	189.6667	3.678E-38	112.8889	1.6306E-16
mmu-miR-470*	55.72222	0.3149875	262.9259	3.678E-38	82.7037	9.56079E-12	50.88889	0.6208503
mmu-miR-471	71.77778	0.00121618	49.88889	0.4881071	69.62963	5.6789E-06	50.77778	0.6266137
mmu-miR-471:9.1	366.1111	3.678E-38	47.96296	0.6270539	46.7037	0.658602	48.11111	0.7540524
mmu-miR-483	43	0.8948983	71.88889	2.46961E-05	213.7778	3.678E-38	47.88889	0.7635178
mmu-miR-483*	249.8333	3.678E-38	66.96296	0.000751681	224.3333	3.678E-38	79.33334	0.000172356
mmu-miR-484	4175.611	3.678E-38	3560.667	3.678E-38	3354.37	3.678E-38	1888.222	3.678E-38
mmu-miR-485	47.38889	0.7465656	430.5185	3.678E-38	201.1481	3.678E-38	45.88889	0.8391483
mmu-miR-485*	55.88889	0.3261093	52.18518	0.3269123	306.3704	3.678E-38	57.33333	0.2833506
mmu-miR-486	7212	3.678E-38	2413.296	3.678E-38	3285.481	3.678E-38	97.55556	6.44872E-10
mmu-miR-487b	75.72222	0.003096281	68.40741	0.000233124	65.40741	9.5674E-05	99.66666	1.02825E-10
mmu-miR-488	46.55556	0.7762489	47.11111	0.7000406	60.40741	0.0024414	48.33333	0.7443873
mmu-miR-488*	555.1667	3.678E-38	98.48148	3.15002E-20	78.51852	2.61491E-09	495.1111	3.678E-38
mmu-miR-489	1046	3.678E-38	638	3.678E-38	304.1852	3.678E-38	1353.778	3.678E-38
mmu-miR-490	106.4444	3.44948E-18	393.8889	3.678E-38	122.7407	3.678E-38	1162.889	3.678E-38
mmu-miR-491	1198.5	3.678E-38	163.9259	3.678E-38	53.14815	0.2008342	194	3.678E-38
mmu-miR-493	212.1667	3.678E-38	125.4815	3.678E-38	139.3333	3.678E-38	190.8889	3.678E-38
mmu-miR-494	602.5555	3.678E-38	287.1852	3.678E-38	120.3704	3.678E-38	867.4445	3.678E-38
mmu-miR-495	51.27778	0.5207615	46.40741	0.7403803	110.6667	3.678E-38	47.22222	0.7906746
mmu-miR-496	52.94444	0.482841	50.92593	0.3956747	48	0.5818611	75.77778	0.00098964
mmu-miR-497	44.44444	0.8594432	79.44444	2.60156E-08	149.4815	3.678E-38	42.22222	0.932152
mmu-miR-499	4199.778	3.678E-38	1343.333	3.678E-38	4542.593	3.678E-38	5836.111	3.678E-38
mmu-miR-500	492.7222	3.678E-38	939.5185	3.678E-38	389.4815	3.678E-38	3257.111	3.678E-38
mmu-miR-501-3p	282.5	3.678E-38	350.037	3.678E-38	896.4074	3.678E-38	2584	3.678E-38
mmu-miR-501-5p	46.16667	0.7908795	44.92593	0.8183091	618	3.678E-38	45.44444	0.8535306
mmu-miR-503	1524.889	3.678E-38	300.2963	3.678E-38	3280.111	3.678E-38	103.6667	2.53722E-12
mmu-miR-503*	1961.944	3.678E-38	286.7408	3.678E-38	622.8889	3.678E-38	44.22222	0.8885233
mmu-miR-504	16477.11	3.678E-38	12030.44	3.678E-38	14510.67	3.678E-38	19182.55	3.678E-38
mmu-miR-505	134.3889	1.57979E-17	49.11111	0.5687792	48.33333	0.5527506	47.77778	0.7681743
mmu-miR-509-3p	65.55556	0.04717536	50.62963	0.4463719	48.40741	0.5417933	48.77778	0.7244784
mmu-miR-509-5p	51.66667	0.538078	172.4444	3.678E-38	49.11111	0.4777797	2766.333	3.678E-38
mmu-miR-511	1146.167	3.678E-38	245	3.678E-38	118.1481	3.678E-38	1462.222	3.678E-38
mmu-miR-532-3p	2589.944	3.678E-38	899.2593	3.678E-38	1874.407	3.678E-38	1128.778	3.678E-38
mmu-miR-532-5p	47.61111	0.7443584	48.51852	0.5897952	506.6667	3.678E-38	51.55556	0.5857458
mmu-miR-539	48.38889	0.706839	50.11111	0.4742062	70.18519	0.000048192	56.88889	0.3042614
mmu-miR-540-3p	537.8333	3.678E-38	182.5185	3.678E-38	584.2963	3.678E-38	982.8889	3.678E-38
mmu-miR-540-5p	41.44444	0.9281614	42.51852	0.9207352	41.7037	0.9273226	43.77778	0.89963
mmu-miR-541	50.72222	0.5954574	169.8148	3.678E-38	163.963	3.678E-38	58.77778	0.2205611
mmu-miR-542-3p	631.4445	3.678E-38	676.4445	3.678E-38	714.3704	3.678E-38	754.7778	3.678E-38
mmu-miR-542-5p	47.33333	0.7524922	47.85185	0.6433639	47.51852	0.5926598	45.66667	0.8464509
mmu-miR-543	46.05556	0.8128958	49.59259	0.5149719	47.22222	0.6168847	51.88889	0.5679138
mmu-miR-544	48.27778	0.7085742	155.1481	3.678E-38	44.51852	0.8109679	45.66667	0.8464509
mmu-miR-546	349.5	3.678E-38	118.963	8.15121E-37	81.66666	3.30833E-11	3448	3.678E-38
mmu-miR-547	936.5555	3.678E-38	144.5185	3.678E-38	88.25926	2.30359E-15	746.1111	3.678E-38
mmu-miR-551b	70.88889	0.01338341	80.37037	3.59633E-09	56.03704	0.08126591	157	3.678E-38
mmu-miR-551b:9.1	63.55556	0.1021318	57.51852	0.07636913	64.88889	0.000114988	123.3333	4.40451E-22
mmu-miR-568	56.94444	0.2715308	61.18518	0.006739093	49.44444	0.451313	109.5556	6.39452E-15
mmu-miR-574-3p	926.0555	3.678E-38	4458.333	3.678E-38	2649.519	3.678E-38	5217.333	3.678E-38
mmu-miR-574-5p	9590.833	3.678E-38	6965.815	3.678E-38	6049.259	3.678E-38	3413.222	3.678E-38
mmu-miR-582-3p	62.61111	0.07015878	206.9259	3.678E-38	132.3704	3.678E-38	51.22222	0.6034028
mmu-miR-582-5p	799.8889	3.678E-38	368.4074	3.678E-38	404.5926	3.678E-38	854.7778	3.678E-38
mmu-miR-590-3p	43.44444	0.8831587	44.59259	0.8379826	44.55556	0.8060933	50.77778	0.6266137
mmu-miR-590-5p	116.3333	5.82481E-17	75.92593	1.11456E-06	58.03704	0.03416626	56.55556	0.3203811
mmu-miR-592	1085.222	3.678E-38	1297.111	3.678E-38	687.3704	3.678E-38	3484.889	3.678E-38
mmu-miR-598	48.55556	0.6918717	1226.741	3.678E-38	1925.296	3.678E-38	2441.667	3.678E-38
mmu-miR-615-3p	1126.056	3.678E-38	1132.926	3.678E-38	3871.148	3.678E-38	1280	3.678E-38
mmu-miR-615-5p	929.2222	3.678E-38	260.4815	3.678E-38	639.7778	3.678E-38	1299.778	3.678E-38
mmu-miR-652	1907.167	3.678E-38	3161.481	3.678E-38	2753.259	3.678E-38	5104.333	3.678E-38
mmu-miR-653	48.5	0.6961475	47.14815	0.6969168	58.22222	0.008317948	46.11111	0.8316225
mmu-miR-654-3p	8920.056	3.678E-38	5411.704	3.678E-38	2819.741	3.678E-38	6317	3.678E-38
mmu-miR-654-5p	363.6111	3.678E-38	112.4074	1.0186E-29	66.2963	0.000315575	136.2222	3.67811E-30
mmu-miR-665	1059.222	3.678E-38	48.81482	0.5691864	86.85185	3.0249E-14	50.44444	0.6437303
mmu-miR-666-3p	83.5	0.000117914	55.59259	0.1525728	1002.963	3.678E-38	50.66667	0.632349

mmu-miR-666-5p	503.7778	3.678E-38	46.7037	0.7154158	46.7037	0.6745452	2643.333	3.678E-38
mmu-miR-667	1265.222	3.678E-38	559.4815	3.678E-38	62.51852	0.006381213	46.88889	0.8035344
mmu-miR-668	399.1667	3.678E-38	529.037	3.678E-38	641.2593	3.678E-38	77.66666	0.000401987
mmu-miR-669a	3943.889	3.678E-38	1110.815	3.678E-38	980.037	3.678E-38	63.77778	0.07303588
mmu-miR-669b	503.0555	3.678E-38	317.7408	3.678E-38	141.9259	3.678E-38	883.5555	3.678E-38
mmu-miR-669c	5637.167	3.678E-38	3254.185	3.678E-38	1927.63	3.678E-38	2355.444	3.678E-38
mmu-miR-669d	3338.944	3.678E-38	1113.889	3.678E-38	1342.63	3.678E-38	1715.333	3.678E-38
mmu-miR-669e	1026.556	3.678E-38	1176.593	3.678E-38	385.4815	3.678E-38	920.2222	3.678E-38
mmu-miR-669f	12006.67	3.678E-38	2018.889	3.678E-38	4818.852	3.678E-38	61.77778	0.1189507
mmu-miR-669g	44.72222	0.8423081	516.5926	3.678E-38	42.40741	0.909386	43.44444	0.9074137
mmu-miR-669h-3p	2629.778	3.678E-38	312.037	3.678E-38	200.5556	3.678E-38	366.7778	3.678E-38
mmu-miR-669h-5p	106.1667	8.31263E-10	62.66667	0.006753893	60.55556	0.003663411	4757	3.678E-38
mmu-miR-669i	473.1111	3.678E-38	44.03704	0.8587548	369.5926	3.678E-38	121.2222	6.91689E-21
mmu-miR-669j	56.38889	0.2821409	228.963	3.678E-38	51.59259	0.293439	62.11111	0.1101373
mmu-miR-669k	54.66667	0.3728726	52.55556	0.3009324	49.74074	0.4281783	82.66666	2.73485E-05
mmu-miR-670	3505	3.678E-38	102.2222	8.18943E-22	348.1111	3.678E-38	164.6667	3.678E-38
mmu-miR-671-3p	415.6667	3.678E-38	226.0741	3.678E-38	319.4074	3.678E-38	50.33333	0.6493741
mmu-miR-671-5p	466.2778	3.678E-38	86.92593	7.76874E-12	94.85185	2.28006E-26	217.5556	3.678E-38
mmu-miR-672	1473.722	3.678E-38	5011.889	3.678E-38	5257.481	3.678E-38	1567.778	3.678E-38
mmu-miR-673-3p	47	0.7592674	44.92593	0.8183672	44	0.8434626	45.33333	0.8569869
mmu-miR-673-5p	81.16666	3.73403E-05	182.1481	3.678E-38	384.2963	3.678E-38	60.33333	0.1628368
mmu-miR-674	5824.278	3.678E-38	4018.111	3.678E-38	5474.519	3.678E-38	3180.444	3.678E-38
mmu-miR-674*	3615.778	3.678E-38	2828.481	3.678E-38	1937.37	3.678E-38	3965.556	3.678E-38
mmu-miR-675-3p	5082.167	3.678E-38	48.62963	0.5599955	343.4445	3.678E-38	50	0.6661043
mmu-miR-675-5p	686.6111	3.678E-38	279.7037	3.678E-38	246.037	3.678E-38	401.1111	3.678E-38
mmu-miR-676	1188.5	3.678E-38	2518.148	3.678E-38	1781.778	3.678E-38	2552.889	3.678E-38
mmu-miR-676*	48.11111	0.7188234	154.7037	3.678E-38	61.25926	0.004639477	51.55556	0.5857458
mmu-miR-677	2002.111	3.678E-38	683.9259	3.678E-38	416.8889	3.678E-38	129.7778	5.91074E-26
mmu-miR-678	100.5	3.14489E-11	67.40741	0.000410025	55.88889	0.08351159	93.77778	1.40734E-08
mmu-miR-679	926.8333	3.678E-38	377.963	3.678E-38	117.4074	3.678E-38	1115.778	3.678E-38
mmu-miR-680	46.61111	0.7686813	46.66667	0.7202696	50.62963	0.3875293	51.44444	0.5916526
mmu-miR-681	236.7778	3.678E-38	69.40741	9.36116E-05	139.1481	3.678E-38	307.4445	3.678E-38
mmu-miR-682	6988	3.678E-38	3098.148	3.678E-38	1673.148	3.678E-38	2815	3.678E-38
mmu-miR-683	2417.833	3.678E-38	1104.185	3.678E-38	592.037	3.678E-38	548.3333	3.678E-38
mmu-miR-684	1920.278	3.678E-38	718.7407	3.678E-38	91.48148	9.18338E-19	74.66666	0.001633142
mmu-miR-685	3763.5	3.678E-38	5270.741	3.678E-38	3640.074	3.678E-38	2057.778	3.678E-38
mmu-miR-686	52.5	0.4716426	93.14815	3.4625E-16	233.9259	3.678E-38	59.88889	0.1782297
mmu-miR-687	47.77778	0.7282031	48.03704	0.6314704	47.66667	0.6093456	48.66667	0.7295263
mmu-miR-688	43.72222	0.8844689	43.48148	0.8843595	43.77778	0.8529523	43.11111	0.9147417
mmu-miR-689	58.72222	0.2030602	107.8519	2.29777E-22	59.18518	0.03485938	78.22222	0.000304771
mmu-miR-690	15008.89	3.678E-38	22060.45	3.678E-38	23341.59	3.678E-38	18663.67	3.678E-38
mmu-miR-691	218.5556	3.678E-38	115.8148	3.678E-38	173.5185	3.678E-38	110.3333	2.76653E-15
mmu-miR-692	285.5555	3.678E-38	210.5185	3.678E-38	79	2.27808E-09	362.1111	3.678E-38
mmu-miR-693-3p	3018.611	3.678E-38	58.92593	0.04485887	51.55556	0.2849482	79.88889	0.000128561
mmu-miR-693-5p	49.33333	0.6439985	58.11111	0.05390209	52.62963	0.2260643	139.5556	1.81544E-32
mmu-miR-694	712.0555	3.678E-38	338.8889	3.678E-38	106.1852	1.11577E-29	1337.222	3.678E-38
mmu-miR-695	94.72222	1.13043E-09	56.96296	0.07188205	81.88889	4.41231E-15	79.11111	0.000193504
mmu-miR-696	3538.722	3.678E-38	1755.963	3.678E-38	1005.815	3.678E-38	3148.222	3.678E-38
mmu-miR-697	689.9445	3.678E-38	54.22222	0.1991303	49	0.4872956	63.33333	0.08183796
mmu-miR-698	99.72222	8.58742E-09	52.59259	0.3029108	52.77778	0.2089511	96.22222	1.972E-09
mmu-miR-699	1251.944	3.678E-38	2032.296	3.678E-38	5502.704	3.678E-38	222.7778	3.678E-38
mmu-miR-700	1100.333	3.678E-38	330.037	3.678E-38	132.0741	3.678E-38	615.4445	3.678E-38
mmu-miR-701	1057.556	3.678E-38	471.2592	3.678E-38	296.8518	3.678E-38	421.1111	3.678E-38
mmu-miR-702	53.27778	0.4283458	47.62963	0.6612082	46.51852	0.6867253	48.11111	0.7540524
mmu-miR-703	2828.056	3.678E-38	2485.704	3.678E-38	1496.519	3.678E-38	61.22222	0.1347246
mmu-miR-704	55.44444	0.4062512	49.40741	0.4942027	269.4445	3.678E-38	61.33333	0.1314602
mmu-miR-705	49.22222	0.6566032	48.2963	0.6207526	50.74074	0.3912042	45.66667	0.8464509
mmu-miR-706	16638.83	3.678E-38	12063.33	3.678E-38	13134.59	3.678E-38	12283.22	3.678E-38
mmu-miR-707	939.5555	3.678E-38	380.037	3.678E-38	156.1481	3.678E-38	1381.444	3.678E-38
mmu-miR-708	16643.17	3.678E-38	15132.26	3.678E-38	15037.89	3.678E-38	17476.67	3.678E-38
mmu-miR-708*	374.4445	3.678E-38	108.0741	4.945E-26	462.5555	3.678E-38	67.77778	0.02274214
mmu-miR-709	14037.17	3.678E-38	13950.33	3.678E-38	13937.22	3.678E-38	8800.333	3.678E-38
mmu-miR-710	56.5	0.3093163	53.92593	0.2020213	51.44444	0.3067788	65.22222	0.04937633
mmu-miR-711	54.11111	0.3923381	53.55556	0.2516282	49.92593	0.4131644	60.88889	0.1448495
mmu-miR-712	6695.889	3.678E-38	4759.741	3.678E-38	2668.407	3.678E-38	1003.222	3.678E-38
mmu-miR-712*	212.3333	3.678E-38	93.92593	1.47546E-16	290.6296	3.678E-38	226.5556	3.678E-38
mmu-miR-713	54.16667	0.3876613	654.5555	3.678E-38	95.18519	2.17656E-30	47.88889	0.7635178
mmu-miR-714	54.22222	0.4279322	646.963	3.678E-38	428.9259	3.678E-38	54.55556	0.4233487
mmu-miR-715	115.7222	8.42253E-16	1430.889	3.678E-38	410.1111	3.678E-38	523.5555	3.678E-38
mmu-miR-717	66.72222	0.008735938	74.77778	9.95693E-06	71.51852	6.53282E-06	55.66667	0.3649845
mmu-miR-718	75.55556	0.001199308	65.62963	0.001171479	54.55556	0.1313883	106.4444	1.63323E-13
mmu-miR-719	4671.278	3.678E-38	2508.37	3.678E-38	2114.704	3.678E-38	4198.444	3.678E-38
mmu-miR-720	13073.83	3.678E-38	12947.19	3.678E-38	14953.89	3.678E-38	11366.33	3.678E-38
mmu-miR-721	871.3889	3.678E-38	229.2222	3.678E-38	95.44444	1.83928E-20	1522.556	3.678E-38
mmu-miR-741	59.16667	0.1808452	53.2963	0.2522564	244.8519	3.678E-38	326.4445	3.678E-38
mmu-miR-742	50.88889	0.5912684	50.92593	0.4142826	50.81482	0.3715779	55.22222	0.388039
mmu-miR-742*	6771.833	3.678E-38	4287.667	3.678E-38	2252.259	3.678E-38	8065.556	3.678E-38
mmu-miR-743a	56.38889	0.2887072	67.40741	0.000609796	137.2222	3.678E-38	2537.111	3.678E-38
mmu-miR-743b-3p	121.4444	4.5485E-17	73.66666	2.73384E-06	177.2593	3.678E-38	133	5.13316E-28
mmu-miR-743b-5p	44.55556	0.8517061	46.18518	0.7763954	44.11111	0.838087	48.22222	0.7492445

mmu-miR-744	6508.944	3.678E-38	4452.889	3.678E-38	4209.074	3.678E-38	2267.444	3.678E-38
mmu-miR-744*	2614.667	3.678E-38	521.1111	3.678E-38	449.4815	3.678E-38	130.1111	3.64989E-26
mmu-miR-758	536.8333	3.678E-38	124.963	3.678E-38	68.11111	2.39461E-05	741.2222	3.678E-38
mmu-miR-759	3494.389	3.678E-38	3710.593	3.678E-38	532.9259	3.678E-38	4785.778	3.678E-38
mmu-miR-760	2853.167	3.678E-38	919.3333	3.678E-38	511.5185	3.678E-38	2912.222	3.678E-38
mmu-miR-760:9.1	5304.222	3.678E-38	1127.926	3.678E-38	755.5185	3.678E-38	5591.111	3.678E-38
mmu-miR-761	6112.5	3.678E-38	4880.667	3.678E-38	1734.111	3.678E-38	7687.778	3.678E-38
mmu-miR-762	4346.056	3.678E-38	1829.333	3.678E-38	922.5926	3.678E-38	4293	3.678E-38
mmu-miR-763	8895.889	3.678E-38	4757.741	3.678E-38	1769.963	3.678E-38	7900.556	3.678E-38
mmu-miR-764-3p	689.5555	3.678E-38	809	3.678E-38	191.6296	3.678E-38	2384.667	3.678E-38
mmu-miR-764-5p	5624.444	3.678E-38	5313.852	3.678E-38	3174.444	3.678E-38	7622.778	3.678E-38
mmu-miR-770-3p	3372.944	3.678E-38	1923.259	3.678E-38	375.1852	3.678E-38	3688	3.678E-38
mmu-miR-770-5p	5884.056	3.678E-38	5215.148	3.678E-38	3571.593	3.678E-38	52.66667	0.5258263
mmu-miR-7a	6400.556	3.678E-38	5831.889	3.678E-38	7858.037	3.678E-38	6825.222	3.678E-38
mmu-miR-7a*	14799.39	3.678E-38	9491.889	3.678E-38	11218.15	3.678E-38	15553.33	3.678E-38
mmu-miR-7b	536.1111	3.678E-38	2052.037	3.678E-38	2269.778	3.678E-38	87.66666	1.19253E-06
mmu-miR-801:9.1	5483.556	3.678E-38	930.7778	3.678E-38	791.9259	3.678E-38	335.2222	3.678E-38
mmu-miR-802	145.0556	6.78375E-32	190.6667	3.678E-38	143.9259	3.678E-38	82.22222	3.53572E-05
mmu-miR-804	83.16666	2.72251E-05	56.85185	0.08580624	53.25926	0.1918142	59.44444	0.1945091
mmu-miR-805	5109	3.678E-38	2636.704	3.678E-38	2444.889	3.678E-38	52.22222	0.5499422
mmu-miR-871	226.3333	3.678E-38	149.5926	3.678E-38	584.1481	3.678E-38	716.8889	3.678E-38
mmu-miR-872	214.3333	3.678E-38	128.6667	3.678E-38	68.33334	0.000146412	283	3.678E-38
mmu-miR-872*	6806.278	3.678E-38	6245.296	3.678E-38	7720.296	3.678E-38	3393.222	3.678E-38
mmu-miR-873	735.4445	3.678E-38	286.037	3.678E-38	322.7408	3.678E-38	969.2222	3.678E-38
mmu-miR-874	997.9445	3.678E-38	127.963	3.678E-38	94.40741	5.85895E-18	1943.444	3.678E-38
mmu-miR-875-3p	9107.723	3.678E-38	3615.778	3.678E-38	3267.148	3.678E-38	12035.22	3.678E-38
mmu-miR-875-5p	1971	3.678E-38	119.5926	2.80592E-30	46.48148	0.6933344	47.55556	0.7773321
mmu-miR-876-3p	54.38889	0.4312823	461.2592	3.678E-38	116.8889	3.678E-38	1021.222	3.678E-38
mmu-miR-876-5p	47.94444	0.7137511	48.77778	0.5509253	118.5185	3.678E-38	47.44444	0.7818326
mmu-miR-877	2147.889	3.678E-38	4243.481	3.678E-38	2974.333	3.678E-38	350.3333	3.678E-38
mmu-miR-877*	1964.111	3.678E-38	3858	3.678E-38	3903.556	3.678E-38	53.66667	0.4713536
mmu-miR-878-3p	121.3889	1.35236E-22	137.3333	3.678E-38	61.81482	0.004960634	86	3.56092E-06
mmu-miR-878-5p	71.72222	0.005107428	125.3333	3.678E-38	503.2222	3.678E-38	262.8889	3.678E-38
mmu-miR-879	83.94444	0.000229061	55.66667	0.1291755	52.77778	0.2102001	53.66667	0.4713536
mmu-miR-879*	45.55556	0.8197907	45.88889	0.7732774	51.37037	0.2321972	44.44444	0.8826514
mmu-miR-880	45.27778	0.8171732	43.11111	0.8959296	138.7778	3.678E-38	50	0.6661043
mmu-miR-881	2145.833	3.678E-38	353.7408	3.678E-38	517.8889	3.678E-38	3543.556	3.678E-38
mmu-miR-881*	89.61111	2.86908E-08	74.66666	5.80681E-06	119.1111	3.678E-38	86.55556	2.48659E-06
mmu-miR-882	323.0555	3.678E-38	55.40741	0.1114205	51.22222	0.3299852	158.3333	3.678E-38
mmu-miR-883a-3p	91.88889	1.78438E-08	79.03704	1.01759E-11	127.7037	3.678E-38	85.22222	5.83265E-06
mmu-miR-883a-5p	65.05556	0.03657513	50.44444	0.4600379	49.18518	0.4814166	53.66667	0.4713536
mmu-miR-883b-3p	615.1667	3.678E-38	165.7037	3.678E-38	77.44444	9.1558E-09	809.6667	3.678E-38
mmu-miR-883b-5p	69.44444	0.01215088	66.48148	0.000942903	54.03704	0.1536976	62.77778	0.09393643
mmu-miR-9	364.1667	3.678E-38	4548.556	3.678E-38	8815.259	3.678E-38	50.22222	0.6549851
mmu-miR-9*	1944.278	3.678E-38	3725.704	3.678E-38	6932.741	3.678E-38	471.5555	3.678E-38
mmu-miR-92a	13764.72	3.678E-38	8006.407	3.678E-38	8437.667	3.678E-38	6773.111	3.678E-38
mmu-miR-92a*	3822.778	3.678E-38	619.5555	3.678E-38	230.5926	3.678E-38	1860	3.678E-38
mmu-miR-92b	1755.556	3.678E-38	5826.185	3.678E-38	4652.852	3.678E-38	826.8889	3.678E-38
mmu-miR-93	8385.667	3.678E-38	4675.074	3.678E-38	4777.111	3.678E-38	4009	3.678E-38
mmu-miR-93*	3183.5	3.678E-38	555	3.678E-38	726.7778	3.678E-38	91.33334	9.01173E-08
mmu-miR-96	691.1111	3.678E-38	1397.444	3.678E-38	1149.407	3.678E-38	757.5555	3.678E-38
mmu-miR-98	7182	3.678E-38	5427.185	3.678E-38	6819.333	3.678E-38	5147.333	3.678E-38
mmu-miR-99a	48	0.69621	3638.63	3.678E-38	2601.556	3.678E-38	2522	3.678E-38
mmu-miR-99b	735.3333	3.678E-38	6146.741	3.678E-38	4165.704	3.678E-38	3659.333	3.678E-38
mmu-miR-99b*	45.33333	0.8241021	45.59259	0.7843166	61	0.006492534	45.22222	0.8603877
solexa-103-3961	787.5	3.678E-38	1085.148	3.678E-38	1332.259	3.678E-38	53.11111	0.5016153
solexa-110-3896	872.3889	3.678E-38	444	3.678E-38	414.8889	3.678E-38	417.8889	3.678E-38
solexa-1127-427	8787.444	3.678E-38	7818.259	3.678E-38	9396.667	3.678E-38	4866.111	3.678E-38
solexa-1201-400	531.5555	3.678E-38	203.9259	3.678E-38	225.1481	3.678E-38	462	3.678E-38
solexa-1278-371	889.2222	3.678E-38	333.2963	3.678E-38	1059.37	3.678E-38	4088.333	3.678E-38
solexa-130-3526	55.11111	0.367676	50	0.460898	46.37037	0.6874777	69.44444	0.01294367
solexa-1328-360	3967.111	3.678E-38	7265.852	3.678E-38	8998.037	3.678E-38	1651.333	3.678E-38
solexa-1416-339_2	191.5	3.678E-38	1359.407	3.678E-38	61.40741	0.006295532	428.5555	3.678E-38
solexa-173-2522	51.66667	0.521419	1254.185	3.678E-38	1231.074	3.678E-38	68.66666	0.01693506
solexa-1780-267	142.5	1.22073E-32	93.66666	2.89819E-19	104.8148	3.678E-38	179.3333	3.678E-38
solexa-1837-257	4026.556	3.678E-38	1596.926	3.678E-38	2270.074	3.678E-38	2938.778	3.678E-38
solexa-200-2167	556.3333	3.678E-38	1588.148	3.678E-38	718.5926	3.678E-38	513.6667	3.678E-38
solexa-2011-236	45.27778	0.8255843	1162.593	3.678E-38	2257.741	3.678E-38	43.22222	0.9123489
solexa-201-2163	58.27778	0.2528407	61.07407	0.01757363	126.4815	3.678E-38	70.55556	0.008662669
solexa-2012-235	51.11111	0.5557389	44.07407	0.8620071	48.33333	0.4703683	41.22222	0.9483077
solexa-2054-231	423.5555	3.678E-38	144.3333	3.678E-38	70.44444	9.26015E-06	230.6667	3.678E-38
solexa-2307-205	291.1111	3.678E-38	91.25926	2.18751E-14	63	0.002796799	557.4445	3.678E-38
solexa-231-1844	769.3333	3.678E-38	803.963	3.678E-38	559.1111	3.678E-38	67.88889	0.02193497
solexa-239-1823	11368.78	3.678E-38	8994.814	3.678E-38	9892.223	3.678E-38	6343.444	3.678E-38
solexa-2402-197	95.16666	1.36243E-06	51.85185	0.3475721	172.6667	3.678E-38	62	0.1130215
solexa-2546-186	55.72222	0.4088525	55.44444	0.1296687	48.44444	0.5384564	62.66667	0.09650699
solexa-2564-185	3371.722	3.678E-38	1101.222	3.678E-38	951.0741	3.678E-38	2420.333	3.678E-38
solexa-2572-184	64.66666	0.04047309	60.74074	0.0262329	51.14815	0.3248147	78.77778	0.000229815
solexa-27-9416	9312.444	3.678E-38	17847.33	3.678E-38	18761.74	3.678E-38	13852	3.678E-38
solexa-284-1594	7923.778	3.678E-38	5275.296	3.678E-38	6350.741	3.678E-38	61	0.1414191

solexa-3014-156	51.88889	0.5911294	65.40741	0.004881769	63.96296	0.002703401	49.88889	0.6716105
solexa-3024-155	100.1111	6.86949E-10	610.3704	3.678E-38	276.9259	3.678E-38	201.8889	3.678E-38
solexa-3062-153	4855.556	3.678E-38	4784	3.678E-38	2216.963	3.678E-38	5313.556	3.678E-38
solexa-308-1456	2649.944	3.678E-38	5317.704	3.678E-38	5732.704	3.678E-38	4673.778	3.678E-38
solexa-3253-144	8311.611	3.678E-38	9535.37	3.678E-38	7051.889	3.678E-38	6731.778	3.678E-38
solexa-403-1161	3123.389	3.678E-38	5187.444	3.678E-38	3208.889	3.678E-38	4465.556	3.678E-38
solexa-4153-111	1084.611	3.678E-38	445.5555	3.678E-38	624.1111	3.678E-38	93.11111	2.36044E-08
solexa-4179-110	13275.06	3.678E-38	3430.889	3.678E-38	1155.037	3.678E-38	8422.333	3.678E-38
solexa-4327-106	1886.444	3.678E-38	592.1852	3.678E-38	490.1111	3.678E-38	48.77778	0.7244784
solexa-447-1003	2572.667	3.678E-38	3829.37	3.678E-38	3530.148	3.678E-38	2830.778	3.678E-38
solexa-4983-92	1398.944	3.678E-38	4389.296	3.678E-38	1210.704	3.678E-38	7175.444	3.678E-38
solexa-5067-90	671.9445	3.678E-38	856.1481	3.678E-38	97	6.94095E-30	2970.889	3.678E-38
solexa-5306-86	794.6667	3.678E-38	1120.185	3.678E-38	103.4815	1.72397E-37	4402.889	3.678E-38
solexa-5560-82	19003.05	3.678E-38	13203.26	3.678E-38	16908.37	3.678E-38	15002.67	3.678E-38
solexa-5593-81	3160.667	3.678E-38	2003.741	3.678E-38	583.1481	3.678E-38	453.3333	3.678E-38
solexa-564-789	324.7222	3.678E-38	104.2593	1.40723E-23	64.66666	0.000929141	285.5555	3.678E-38
solexa-622-718	6503.889	3.678E-38	9120.519	3.678E-38	8792.519	3.678E-38	2098.778	3.678E-38
solexa-783-586	55.94444	0.2978758	49.22222	0.5307058	50.48148	0.3888818	55.66667	0.3649845
solexa-897-515	2088	3.678E-38	2241.148	3.678E-38	1709.556	3.678E-38	105.2222	5.55635E-13

Supporting Information Table 2. miRNA expression in human sorted thymic cell populations
(average of two biological replicates)

	Tc		mTEC_low		mTEC_high		cTEC	
**TargetID**	Avg_signal	p-val	Avg_signal	p-val	Avg_signal	p-val	Avg_signal	p-val
HS_1	80.8125	0.6391678	78.1875	0.754454	79	0.7679042	77.875	0.7687999
HS_10	84.4375	0.5313092	341.8125	3.678E-38	82.6875	0.691529	109.0625	0.01420164
HS_100	9500.813	3.678E-38	5320.438	3.678E-38	10098.13	3.678E-38	8188.5	3.678E-38
HS_101	83	0.5906218	110.875	0.01847372	88.1875	0.6146315	79.8125	0.7274255
HS_104	929.9375	3.678E-38	203.875	1.20284E-25	79.0625	0.7443505	77.8125	0.7608179
HS_105	271.25	3.678E-38	116.1875	0.03155001	204.3125	1.11408E-14	154.625	7.26771E-05
HS_106	382.6875	4.72357E-38	596.8125	3.678E-38	1972.313	3.678E-38	1410.25	3.678E-38
HS_107	74.625	0.7927057	75.625	0.7977502	77.9375	0.7644401	78.5625	0.730462
HS_108.1	1134.188	3.678E-38	1165.938	3.678E-38	1438.563	3.678E-38	175.125	6.87582E-17
HS_109	94	0.264643	83.75	0.6095418	305.25	3.678E-38	84.5	0.6048616
HS_11.1	108.0625	0.0293226	751.3125	3.678E-38	147.1875	0.007821185	1889.875	3.678E-38
HS_110	176.9375	1.44609E-10	153.1875	1.53333E-05	168.9375	0.000870985	153.125	8.92964E-05
HS_111	653.3125	3.678E-38	425.375	3.678E-38	80.8125	0.6686117	508.8125	3.678E-38
HS_112	74.9375	0.7834842	2266.813	3.678E-38	485.1875	2.24406E-37	87.0625	0.413836
HS_113	548.375	3.678E-38	267.3125	3.54372E-32	369.4375	2.10504E-32	303	1.16372E-35
HS_114	210.4375	2.28888E-22	231.8125	1.72564E-19	305.8125	6.76186E-16	212.5625	4.68222E-13
HS_115	87.1875	0.4282857	83	0.6347563	96.1875	0.2553122	80.5	0.7163349
HS_116	86.75	0.4788907	85.1875	0.5636503	92.375	0.3126391	119.8125	0.000677306
HS_117	86.625	0.4132588	92.8125	0.2484006	79.6875	0.7567543	79.3125	0.7138371
HS_119	116.0625	0.02644924	98.1875	0.2111559	100.6875	0.2640539	94.25	0.3026854
HS_12	79.375	0.6716195	77.1875	0.7669339	84.1875	0.5862792	77.6875	0.7636475
HS_120	141.1875	2.90536E-05	107.6875	0.1057583	2880.375	3.678E-38	165.25	3.59952E-08
HS_121	136.125	0.005108793	211.1875	1.54251E-13	128.375	0.1094877	96.5	0.3148302
HS_122.1	312.1875	3.678E-38	178.0625	7.10095E-09	2973.875	3.678E-38	219.875	3.44215E-14
HS_123	185.25	1.22241E-15	172.125	4.06933E-12	182.125	2.75624E-06	136.5625	0.003982408
HS_124	76.1875	0.7321187	76.5	0.7807597	79.375	0.7599845	77.1875	0.7691624
HS_126	442.875	3.678E-38	164.625	1.07657E-06	198.5	1.66147E-05	203.5	2.78597E-11
HS_127.1	92	0.3202505	87.3125	0.5089136	382.4375	3.678E-38	88.9375	0.4855389
HS_128	148.4375	4.39792E-07	101.625	0.1824458	114.375	0.1665017	105.875	0.1323721
HS_129	4158.813	3.678E-38	88.625	0.5033144	90.1875	0.5877782	1262.438	3.678E-38
HS_13	82.625	0.543473	1959.875	3.678E-38	193.0625	8.03055E-20	82.6875	0.5950689
HS_130	2840.375	3.678E-38	129.75	0.003115191	3218.625	3.678E-38	244.4375	1.08663E-17
HS_131	107.8125	0.04915344	91.125	0.3870161	117.3125	0.2167962	87.5	0.4856654
HS_132.1	78.8125	0.6832169	94.75	0.2192155	78.5625	0.7496473	82.1875	0.6403969
HS_133.1	140	6.33348E-05	98.375	0.1959644	107.625	0.1983758	101	0.1871173
HS_134	891.375	3.678E-38	321.1875	3.678E-38	117.6875	0.2008292	95.625	0.3149397
HS_135	85.1875	0.5207799	80.8125	0.6917738	90.4375	0.4318149	83.625	0.6080446
HS_136	93.4375	0.3595956	88.25	0.5467996	78.375	0.7388268	112.375	0.1159833
HS_137	105.125	0.08261481	87.875	0.490543	93.375	0.5079427	94.3125	0.3621789
HS_138	341.3125	3.678E-38	151.1875	3.36012E-05	389.75	6.1023E-27	283.875	1.17872E-27
HS_139	936.4375	3.678E-38	1460.75	3.678E-38	1235.625	3.678E-38	301.3125	2.21649E-37
HS_14.1	103.375	0.139977	93.5	0.349113	88	0.5457514	86.625	0.4688666
HS_140	76.25	0.7528927	79.375	0.6986117	77.875	0.7650768	76.4375	0.7761087
HS_141	115.3125	0.01448154	95.4375	0.2736491	159.25	0.005319255	97.8125	0.2042438
HS_142.1	104.5625	0.1069341	91	0.3841475	101.875	0.2763097	148.6875	1.48832E-08
HS_143	81.3125	0.6769903	440.625	3.678E-38	88.875	0.5891872	87.75	0.5365784
HS_144	96.375	0.2093781	97.9375	0.2667933	93.25	0.433055	91.5625	0.3952904
HS_145.1	351.5625	3.678E-38	185.25	2.08496E-11	267.6875	5.38513E-14	218.8125	1.2106E-14
HS_146.1	98.375	0.191934	99.125	0.2244369	105.3125	0.298628	94.875	0.3395372
HS_147	95.1875	0.2471575	91.375	0.3715383	92.125	0.4530155	88.8125	0.4323062
HS_149	3859.25	3.678E-38	2035.438	3.678E-38	1821.125	3.678E-38	1767.125	3.678E-38
HS_15.1	83.5	0.53618	84.4375	0.5809446	89.375	0.5658073	85.1875	0.5682094
HS_150	3037.625	3.678E-38	221.4375	2.98475E-18	3114.438	3.678E-38	202.25	3.53527E-12
HS_151.1	86.5	0.3530577	73.4375	0.842966	73.9375	0.8490898	75.0625	0.8200403
HS_152	579.125	3.678E-38	307.375	3.678E-38	470.25	3.678E-38	359.0625	3.678E-38
HS_153	119.625	0.04996531	97	0.2301991	112.1875	0.1353279	97.875	0.2218167
HS_154	75.875	0.7647967	115	0.03623802	129.9375	0.08318078	101.1875	0.1747689
HS_155	73.3125	0.8124889	74.9375	0.8164341	76.0625	0.8002634	71.4375	0.8674555
HS_156	108.125	0.2450223	81.875	0.6674452	653.25	3.678E-38	85.75	0.5858772
HS_157	288.125	1.02912E-29	200.0625	4.32596E-21	207.8125	4.81244E-16	151.375	3.88557E-05
HS_159	2680.875	3.678E-38	82.875	0.6458334	83.4375	0.6574776	81.875	0.6503905
HS_16	101.375	0.1937336	160.9375	1.47716E-06	1814.813	3.678E-38	176.875	1.19041E-07
HS_160	388.4375	3.678E-38	225.375	5.87021E-21	251.8125	1.24503E-10	233.375	8.88793E-17
HS_161	85.4375	0.500668	81.5625	0.6847176	80.625	0.7307287	78.25	0.7511913
HS_162	85.8125	0.5318864	90.9375	0.3623516	93.4375	0.3703491	90.75	0.3431334
HS_163	113.5	0.1232994	88.0625	0.4950311	91.75	0.4737829	90.8125	0.4155388
HS_164	89.8125	0.3843307	85.125	0.5694758	84.625	0.6697868	83.1875	0.6368542
HS_166.1	122.0625	0.007985999	377.8125	3.678E-38	118.4375	0.1040548	281.4375	3.678E-38
HS_167.1	185.5	1.12329E-16	111.6875	0.05301733	144.3125	0.01940723	143.4375	0.000657974
HS_168	110.4375	0.04457369	105.4375	0.1110239	104.5	0.2761092	100.25	0.2318503
HS_169	80.8125	0.6203087	97.4375	0.1601	80.8125	0.6454154	76.6875	0.7856081
HS_17	80.625	0.678223	81.5625	0.6558357	663.3125	3.678E-38	79.8125	0.6797349
HS_170	96.125	0.317347	110.125	0.08557174	119.1875	0.1749308	308.125	5.87633E-31
HS_174.1	86.8125	0.4466145	81.0625	0.6686104	83.4375	0.6732011	92.875	0.427646
HS_175	127.375	0.000341932	94.5625	0.298066	125.4375	0.1134582	124	0.003974238
HS_176	891.3125	3.678E-38	239.8125	1.47064E-36	240.75	9.23217E-23	163.3125	9.43536E-07

HS_177	3395.375	3.678E-38	91.1875	0.3962991	91.9375	0.4554772	1737.938	3.678E-38
HS_179	103.3125	0.1058583	2307.438	3.678E-38	104.9375	0.2104364	101.75	0.2103019
HS_18	2418.313	3.678E-38	96.0625	0.3326772	82.0625	0.7010897	1815.625	3.678E-38
HS_182.1	2799.438	3.678E-38	1895.063	3.678E-38	353.6875	3.678E-38	2488.813	3.678E-38
HS_183.1	893.375	3.678E-38	74.875	0.8154361	74.75	0.8218871	867	3.678E-38
HS_184	417.125	3.678E-38	2561.063	3.678E-38	2245.375	3.678E-38	273.1875	9.09622E-27
HS_185.1	81.5	0.617413	81.4375	0.668021	322.4375	3.678E-38	80.0625	0.693019
HS_186	272.6875	3.678E-38	284.625	3.678E-38	309.0625	1.60853E-21	282.125	2.57893E-32
HS_187	114	0.02868587	88.625	0.408613	91.3125	0.3933892	475.125	3.678E-38
HS_188	1832.438	3.678E-38	710.625	3.678E-38	995.875	3.678E-38	2323.5	3.678E-38
HS_189.1	77.75	0.7439888	78.4375	0.7432714	80.6875	0.7289453	106.8125	0.02359976
HS_19	658.3125	3.678E-38	216.6875	1.81059E-17	301.3125	3.51643E-18	249.625	2.03458E-20
HS_190	107.8125	0.01487642	80.875	0.6692414	152.9375	8.8793E-08	81.4375	0.6616856
HS_192.1	6780.5	3.678E-38	3344	3.678E-38	5157.375	3.678E-38	3683.813	3.678E-38
HS_193	113.125	0.02879914	450.5	3.678E-38	629.9375	3.678E-38	125.5	0.01358608
HS_194	77.375	0.7442266	381.6875	3.678E-38	102.1875	0.0684097	450.25	3.678E-38
HS_195	90.8125	0.329329	82.6875	0.6416393	94.5625	0.3588424	83.75	0.618771
HS_196.1	280.8125	3.678E-38	119.75	0.001225807	119.75	0.002325992	439.9375	3.678E-38
HS_197	98.1875	0.219875	83.25	0.6250032	87.25	0.5582065	88.4375	0.5149632
HS_198	88	0.4382966	74.5	0.8298458	76.125	0.7921544	78.5625	0.7544517
HS_199	660.9375	3.678E-38	167.4375	1.02318E-07	242.375	2.62485E-09	228.375	7.52287E-16
HS_2	116.25	0.02161717	103.4375	0.1393736	113.125	0.1677212	96.5	0.2684074
HS_20	513.125	3.678E-38	304.375	3.678E-38	377.75	1.65427E-35	1154.25	3.678E-38
HS_200	170.5625	2.70227E-09	308.5625	3.678E-38	171.875	2.95698E-11	106.9375	0.1135972
HS_201	165.6875	7.21615E-11	111.375	0.0586325	168	0.002708677	155.75	8.61844E-05
HS_202.1	4565.25	3.678E-38	3449.688	3.678E-38	4202.438	3.678E-38	2236.75	3.678E-38
HS_203	217.6875	9.54481E-20	110.6875	0.04812682	111.6875	0.1227423	164.625	7.76945E-06
HS_204.1	5781.063	3.678E-38	4320.813	3.678E-38	5144.438	3.678E-38	5058.688	3.678E-38
HS_205.1	94.4375	0.2295738	85.125	0.5745741	82.8125	0.6746777	85.5	0.5555322
HS_206	1465.813	3.678E-38	73.75	0.8380561	105.25	0.03252166	177.5625	5.68029E-19
HS_208	101.4375	0.1441096	93	0.3120299	107.8125	0.1966498	113.0625	0.06935125
HS_209.1	90.9375	0.3084387	93.75	0.2757771	350.3125	3.678E-38	80.6875	0.6700654
HS_21	101.25	0.09381421	86.3125	0.5404369	86.9375	0.5649058	90.125	0.4410149
HS_211	91.625	0.3279975	87.5625	0.4889856	85.6875	0.624668	86.0625	0.5492273
HS_215	97.3125	0.2564414	85.6875	0.5652334	89.0625	0.5554822	93	0.3961912
HS_216	204.5625	5.24537E-34	99.5	0.2374979	110.9375	0.0819151	88.3125	0.4741397
HS_217	5320.438	3.678E-38	1837.125	3.678E-38	3817.75	3.678E-38	3263.188	3.678E-38
HS_218	82.625	0.6069982	82.3125	0.6573715	84	0.6591724	84.25	0.5924274
HS_219	230.5625	6.96499E-28	142.875	0.000119493	342.0625	3.678E-38	247.375	1.28912E-34
HS_22.1	956.125	3.678E-38	429	3.678E-38	433.1875	3.678E-38	420.25	3.678E-38
HS_220	94.25	0.2215603	87.625	0.5141714	89.125	0.5768813	87.4375	0.540915
HS_221	1148.188	3.678E-38	74.4375	0.8188075	73.5	0.8357841	97.5625	0.1112578
HS_228.1	4061.563	3.678E-38	4005.688	3.678E-38	4156.938	3.678E-38	4375.688	3.678E-38
HS_23	166.25	9.71363E-13	102.8125	0.156419	119.875	0.1169946	106.25	0.1087913
HS_231	657.6875	3.678E-38	75.25	0.8054417	75.625	0.791189	76.5	0.7790973
HS_232	85.0625	0.5275073	155.9375	3.73298E-10	86.0625	0.4972512	171.75	2.41872E-16
HS_239	3510.25	3.678E-38	2102.375	3.678E-38	2544.75	3.678E-38	1161.813	3.678E-38
HS_24	1098.125	3.678E-38	86.8125	0.5198893	86.75	0.608696	254.5	3.678E-38
HS_240	1084.563	3.678E-38	83.9375	0.6053059	84.3125	0.6854143	84.5625	0.5861143
HS_241.1	97.6875	0.4299631	92.9375	0.310049	92.75	0.4135316	84.875	0.5756218
HS_242	7592.5	3.678E-38	3893	3.678E-38	4262.25	3.678E-38	3552.063	3.678E-38
HS_243.1	2662.938	3.678E-38	2793.75	3.678E-38	834.75	3.678E-38	1942.688	3.678E-38
HS_244	9204.75	3.678E-38	4430	3.678E-38	7304	3.678E-38	5561.938	3.678E-38
HS_25	193.25	1.0818E-09	107	0.1010105	175.8125	1.71836E-09	116.125	0.04424134
HS_250	88.375	0.4119157	111.6875	0.01626609	212.625	2.10158E-24	108.625	0.1225163
HS_251.1	413.75	2.19786E-30	82.1875	0.6654904	584.3125	3.678E-38	546.0625	3.678E-38
HS_252.1	110.5625	0.1078247	100.0625	0.151612	108.4375	0.1341759	100.75	0.1703982
HS_253	84.125	0.5521922	82.5	0.641596	85.75	0.6401555	80.375	0.695707
HS_254	95.8125	0.4265135	342.375	3.678E-38	93.5625	0.3046351	77.9375	0.7332845
HS_255	92.6875	0.3585925	87.75	0.5223383	87.25	0.6264512	243.75	3.678E-38
HS_257	383.0625	3.678E-38	80.375	0.7128949	80.3125	0.7502241	92.8125	0.2954199
HS_258	102.625	0.09266852	86.1875	0.5152971	89.625	0.5651623	82	0.6088474
HS_26.1	105.5	0.1427684	98.75	0.1809969	118.1875	0.1989822	86.4375	0.5267301
HS_260	86.4375	0.5545036	518.625	3.678E-38	185.25	2.36166E-17	84.6875	0.5794339
HS_261.1	92.1875	0.5219145	80.0625	0.7166114	83.75	0.5685518	76.5625	0.7958754
HS_262.1	113.6875	0.04207221	120.1875	0.01473654	136.5625	0.02791405	101.875	0.1751464
HS_263.1	74.9375	0.7883365	543.625	3.678E-38	3299.188	3.678E-38	227.4375	3.678E-38
HS_264.1	76.1875	0.7728467	78.6875	0.7415238	76.9375	0.7948996	76.75	0.769613
HS_265.1	123.125	0.003634594	91.5625	0.3871196	100.0625	0.3615092	106.6875	0.1370386
HS_266.1	76	0.7556687	76.5625	0.7878339	74.0625	0.8252068	75	0.8145706
HS_267	226.5	6.54069E-26	126	0.007441762	214.5	4.68974E-06	152.375	0.000191227
HS_268	410.0625	3.678E-38	155.5	1.52034E-06	208.9375	3.95394E-08	176.25	6.35231E-08
HS_269	221.6875	1.9266E-37	96.0625	0.2996762	142.6875	0.03542992	141.625	0.002009335
HS_27	236.1875	1.15145E-25	130.75	0.001666528	195.1875	7.94218E-06	287.625	8.23289E-28
HS_273	87.0625	0.462915	83.3125	0.6137058	86.375	0.5525987	85	0.5824293
HS_275	522.875	3.678E-38	322.1875	3.678E-38	379.5	3.678E-38	305.6875	3.678E-38
HS_276.1	2213.375	3.678E-38	847.0625	3.678E-38	1573.75	3.678E-38	1145.688	3.678E-38
HS_278	79.75	0.6376135	76.75	0.7724912	78.0625	0.763164	77	0.7656472
HS_279_a	375.125	3.678E-38	175.1875	1.10449E-08	290.3125	1.87485E-14	247.375	2.9834E-21
HS_280_a	79.375	0.6879937	81.5	0.6288096	78.625	0.7573748	78.25	0.7478123

HS_280_b	78.25	0.7328352	658.25	3.678E-38	76.0625	0.8002634	126.75	2.55657E-05
HS_282	110.3125	0.03014742	93.5625	0.3567119	103.75	0.3506439	92.625	0.4098988
HS_283_a	87.25	0.4586374	78.875	0.7169912	81.4375	0.7048073	2127.438	3.678E-38
HS_283_b	76.1875	0.7469796	74.625	0.821757	75.75	0.8113304	75.75	0.8083521
HS_284	89.25	0.4636819	107.625	0.1233297	87.875	0.5891529	84.5	0.6109838
HS_284.1	110.5625	0.02817893	156.375	8.97931E-07	184.375	1.25801E-15	146.4375	1.45937E-07
HS_285	96.6875	0.2122054	103.5625	0.1473661	95.75	0.440993	91.5625	0.3749895
HS_286_a	87.4375	0.4560145	80.9375	0.6658849	80.4375	0.7070657	110.4375	0.01430782
HS_287	1229.313	3.678E-38	91.3125	0.4114232	97.5625	0.2898084	91.4375	0.422567
HS_29	2444.438	3.678E-38	1714.063	3.678E-38	2130.75	3.678E-38	1865.063	3.678E-38
HS_3	73.6875	0.8100395	73.625	0.8407534	74.5625	0.8066677	88.0625	0.3564783
HS_30	78.1875	0.7026547	77.3125	0.7776045	76.3125	0.7796969	77.1875	0.7739818
HS_303_a	997.8125	3.678E-38	518.625	3.678E-38	418.75	3.678E-38	278.5	3.678E-38
HS_303_b	1755.563	3.678E-38	194.375	8.71467E-20	237.5	5.12477E-36	211.5625	4.1945E-28
HS_304_a	133.9375	0.000431255	113.1875	0.0432904	126.375	0.06117477	268.5625	3.678E-38
HS_304_b	628.3125	3.678E-38	1420.125	3.678E-38	154.5	0.01295374	182.6875	1.32017E-07
HS_305_b	1421.875	3.678E-38	195.375	2.10311E-22	2192.563	3.678E-38	129.5625	1.42052E-05
HS_31.1	414.375	2.3212E-35	95.625	0.304112	162.3125	1.77432E-08	106.3125	0.07483418
HS_32	226.5	4.38794E-21	561.5	3.678E-38	448.8125	3.678E-38	199.375	3.17491E-14
HS_33	634.9375	3.678E-38	178.0625	2.82512E-09	299.5	3.57301E-14	1583.563	3.678E-38
HS_35	77.1875	0.7355703	78.5	0.7492279	76.8125	0.7747573	75.75	0.7764647
HS_36.1	84.375	0.5421046	83.875	0.5916544	82.6875	0.6938637	82.125	0.6306426
HS_37	88.1875	0.3887422	99.625	0.1230787	80.625	0.6768479	153.4375	1.99972E-10
HS_38.1	711.125	3.678E-38	315.875	3.678E-38	498.5	3.678E-38	372.5625	3.678E-38
HS_4.1	120.0625	0.000938725	94.3125	0.3051275	90	0.5431874	93	0.3648445
HS_40	528.5	3.678E-38	128.625	0.001858231	160.0625	0.000937381	123.875	0.01241225
HS_41	80.5	0.6510165	75.625	0.8019091	75.4375	0.8087318	78.5625	0.730462
HS_42	92.5	0.3349212	229.125	4.68441E-37	85	0.6232268	86.6875	0.5194427
HS_43.1	91.9375	0.304824	86	0.5557952	87.6875	0.5838094	90.4375	0.439849
HS_44.1	84.625	0.4805598	77.4375	0.77652	76.1875	0.8000341	84.8125	0.6250464
HS_45.1	127.5	0.000403422	118	0.02847035	154.8125	0.01316396	2277.313	3.678E-38
HS_46	85.8125	0.4146937	79.5625	0.7202659	78.75	0.7663741	83.3125	0.651256
HS_47	139.8125	0.001901669	106.125	0.07791065	117.625	0.069749	105.8125	0.09179962
HS_48.1	105.1875	0.06491666	123.9375	0.004588783	120.4375	0.085622	127.4375	0.007401892
HS_49	96.5	0.2509566	101.3125	0.2047956	112.9375	0.2273095	96.75	0.3009234
HS_5.1	4126.875	3.678E-38	86.25	0.5130926	103.75	0.1311482	227.1875	3.678E-38
HS_50	78.5625	0.6995087	76.8125	0.7747371	79.8125	0.7231118	79.125	0.7313358
HS_51	172.875	4.48033E-09	100.9375	0.1465661	2319.875	3.678E-38	96.6875	0.337153
HS_52	1660.438	3.678E-38	639.4375	3.678E-38	1218.438	3.678E-38	1020.938	3.678E-38
HS_53	74.625	0.7867036	74.375	0.8197737	74.1875	0.8309241	76.8125	0.7875819
HS_54	600.1875	3.678E-38	95.375	0.2198786	81.8125	0.7142058	107.5	0.02680775
HS_55	74.6875	0.7824062	75.5	0.8085351	74.3125	0.8340491	74.75	0.8130693
HS_55.1	76.0625	0.7627589	75.875	0.7988383	75.125	0.8255515	77.3125	0.7663693
HS_56	85.4375	0.4824094	169.8125	9.66853E-14	80.4375	0.7195046	96.3125	0.1736505
HS_57.1	85.6875	0.5203872	85.375	0.5490362	2692.75	3.678E-38	85.6875	0.5715674
HS_58	79.1875	0.6829536	80.9375	0.6772885	78	0.7857215	97.9375	0.1056556
HS_59	88.9375	0.4042128	237.1875	3.678E-38	84.0625	0.651068	135.1875	3.45286E-06
HS_6	606.6875	3.678E-38	83.3125	0.6158345	86.4375	0.6290944	109.0625	0.01449199
HS_60	121.375	0.002176088	100.625	0.1813022	112.8125	0.1931093	93.4375	0.3396533
HS_61	75	0.7779126	77.25	0.7595007	75	0.8153248	75.3125	0.7999536
HS_62	181	1.3055E-13	110.375	0.07748294	101.4375	0.3252295	122.25	0.02435228
HS_63	80.8125	0.6391678	98.5	0.2310455	84	0.5899891	152.3125	1.9004E-10
HS_64	88.4375	0.4404183	333.875	3.678E-38	77.6875	0.758775	135.9375	1.18667E-06
HS_65	182.5625	1.40329E-06	108.0625	0.09597129	172.875	5.90722E-09	1517.625	3.678E-38
HS_66	82.9375	0.558872	79.6875	0.7152747	79.1875	0.7546554	77.1875	0.7667313
HS_67	81.375	0.6549897	74.625	0.821757	77.8125	0.7394852	77.75	0.7526878
HS_68	113.875	0.05027651	94.6875	0.3189982	98.1875	0.4054882	100.125	0.2504217
HS_69	71.125	0.8437604	72.375	0.8604097	74.5625	0.7992959	75.625	0.7923495
HS_7	98.1875	0.1759862	1415.063	3.678E-38	377.9375	1.01034E-22	97.4375	0.1919286
HS_70	77.125	0.7083987	76.8125	0.7797185	77.875	0.7620041	77.25	0.7761933
HS_71.1	564.5625	3.678E-38	262	6.40593E-30	432.375	3.678E-38	328.875	3.678E-38
HS_72	89.8125	0.3106881	86.75	0.5228314	80.8125	0.7417229	87.6875	0.5588974
HS_73.1	90.625	0.423182	91.25	0.3262274	100.75	0.2199165	95	0.2600199
HS_74	89.1875	0.4414771	79.0625	0.718285	96.5625	0.3307744	86.5625	0.5072088
HS_75.1	127.8125	0.000206151	100.625	0.1897381	113.6875	0.1924483	103.5625	0.1617827
HS_76	78.8125	0.6727717	79.375	0.7239618	94.5625	0.2336337	78.75	0.7428867
HS_77	77.8125	0.7000229	78.625	0.7385013	77.4375	0.7872778	79.375	0.7392545
HS_78	316.875	3.678E-38	86.75	0.506554	92.6875	0.4746422	100.6875	0.2155357
HS_79.1	97.1875	0.1160114	85.6875	0.5557189	84.8125	0.6467815	85.3125	0.5916191
HS_8	84.1875	0.6272619	86.625	0.5427227	84.1875	0.6258773	90.1875	0.4106016
HS_80	121.25	0.01311645	104.25	0.09307442	113.75	0.1003539	104.8125	0.1020704
HS_81	1878.313	3.678E-38	265.9375	6.99795E-33	366.5	4.35957E-33	335.0625	3.678E-38
HS_82.1	79.9375	0.7129069	80.0625	0.70327	82.75	0.7081344	85.3125	0.5916191
HS_83.1	87.875	0.4564976	85.75	0.5732701	86.875	0.6160154	88.8125	0.5167252
HS_84	82.3125	0.5951856	84.875	0.5540537	85.875	0.6373603	79.5625	0.7175379
HS_85.1	78.1875	0.708394	75.875	0.7935837	78.125	0.7706593	299.8125	1.15307E-27
HS_86	116.25	0.02921768	157.5625	2.30914E-06	155.625	0.004224753	181.625	2.70299E-08
HS_87	82.0625	0.6644084	114.3125	0.008413716	84.375	0.6558282	118.1875	0.001214222
HS_88	77.1875	0.7542785	78.75	0.7256913	78.4375	0.7413868	78.8125	0.7172425
HS_89	87.6875	0.4164984	91.875	0.385773	93.5	0.4890658	91.75	0.4069245

HS_9	89.9375	0.4277915	371.9375	3.678E-38	87.25	0.5971866	90.6875	0.4420595
HS_90	168.875	5.08162E-07	128.6875	0.002018508	153.5	0.001777052	153.6875	4.18781E-05
HS_91.1	76.75	0.7499433	158.25	4.33688E-11	74.875	0.824261	75.8125	0.7880141
HS_92	107.6875	0.0582504	94.625	0.3117286	90.875	0.5237431	87.125	0.4966118
HS_93	173.375	1.84236E-07	126.5	0.01111502	146.4375	0.02555617	116.5625	0.0579565
HS_94	437.3125	3.678E-38	261.75	1.18326E-31	564.5	3.678E-38	261.8125	6.30674E-25
HS_95	140.9375	0.000490757	100.125	0.2125782	98.5	0.3848757	97.3125	0.2650097
HS_96	1350.063	3.678E-38	620.5625	3.678E-38	1142.625	3.678E-38	730.4375	3.678E-38
HS_97	670.75	3.678E-38	317	3.678E-38	486.0625	3.678E-38	569.3125	3.678E-38
HS_99.1	301.3125	3.678E-38	161.75	1.48078E-06	233.75	2.55919E-08	265.5625	2.03354E-23
hsa-let-7a	23846.44	3.678E-38	23846.44	3.678E-38	24157.5	3.678E-38	23846.44	3.678E-38
hsa-let-7a*	78.9375	0.7248011	401.8125	3.678E-38	223.9375	2.78966E-32	236.9375	3.678E-38
hsa-let-7b	10082.56	3.678E-38	12873.88	3.678E-38	14478.31	3.678E-38	14724.63	3.678E-38
hsa-let-7b*	5730.063	3.678E-38	2515.063	3.678E-38	1108.375	3.678E-38	1875.188	3.678E-38
hsa-let-7c	3615.25	3.678E-38	15027.5	3.678E-38	16592.63	3.678E-38	12781.44	3.678E-38
hsa-let-7c*	143.125	1.116E-07	198.125	4.50668E-20	946.625	3.678E-38	148.3125	9.36353E-07
hsa-let-7d	16393.44	3.678E-38	15151.19	3.678E-38	16593.19	3.678E-38	14844.69	3.678E-38
hsa-let-7d*	10009.19	3.678E-38	8373	3.678E-38	8106.5	3.678E-38	7464.625	3.678E-38
hsa-let-7e	7388.688	3.678E-38	17579.75	3.678E-38	17513.38	3.678E-38	16980.06	3.678E-38
hsa-let-7e*	352.4375	3.678E-38	351.25	3.678E-38	337.9375	1.3594E-24	285.1875	1.60041E-37
hsa-let-7f	17583.44	3.678E-38	15738.13	3.678E-38	17186.94	3.678E-38	17189.38	3.678E-38
hsa-let-7f-1*	446.25	3.678E-38	156.0625	1.75557E-08	258.5625	3.678E-38	207.75	8.62078E-31
hsa-let-7f-2*	173	1.33971E-12	143.5625	0.000291366	169.9375	0.002083588	125.125	0.01720639
hsa-let-7g	20983.31	3.678E-38	21741.38	3.678E-38	19652.94	3.678E-38	20237.88	3.678E-38
hsa-let-7g*	109.4375	0.300047	143.25	1.56548E-07	102.875	0.06847247	85.125	0.4797998
hsa-let-7i	11704.63	3.678E-38	11897	3.678E-38	11897	3.678E-38	11451.06	3.678E-38
hsa-let-7i*	89.4375	0.3909903	304.6875	3.678E-38	173.3125	6.14301E-14	209.75	9.84573E-32
hsa-miR-1	77.8125	0.7419361	3811.688	3.678E-38	776.5	3.678E-38	574.8125	3.678E-38
hsa-miR-100	216.25	8.1369E-09	15584.94	3.678E-38	14362.75	3.678E-38	13630.94	3.678E-38
hsa-miR-100*	1786.75	3.678E-38	974.375	3.678E-38	82.9375	0.6488782	182.125	3.70563E-20
hsa-miR-101	1654.438	3.678E-38	13030.81	3.678E-38	3540.688	3.678E-38	7088.313	3.678E-38
hsa-miR-101*	479	3.678E-38	217.375	5.89784E-31	82.5	0.6866833	92.1875	0.2865216
hsa-miR-103	4819	3.678E-38	6575.5	3.678E-38	4052.75	3.678E-38	5140.938	3.678E-38
hsa-miR-105	1162.625	3.678E-38	466.5625	3.678E-38	1234.125	3.678E-38	757.75	3.678E-38
hsa-miR-105*	131.0625	0.000146326	107	0.07798948	95.6875	0.3173147	95.0625	0.2729078
hsa-miR-106a	8706.25	3.678E-38	6319.063	3.678E-38	4698.875	3.678E-38	2922.938	3.678E-38
hsa-miR-106a*	104.75	0.08272747	93.875	0.2985624	89.5625	0.55156	89.25	0.4854194
hsa-miR-106a:9.1	102.375	0.1160462	98.625	0.2524948	99.5	0.4118229	123	0.001981272
hsa-miR-106b	5201.875	3.678E-38	4825.75	3.678E-38	4139.875	3.678E-38	2946.438	3.678E-38
hsa-miR-106b*	90.875	0.4344252	1006.313	3.678E-38	508.3125	3.678E-38	938.0625	3.678E-38
hsa-miR-107	2654.625	3.678E-38	1543.438	3.678E-38	206.875	3.534E-24	787.4375	3.678E-38
hsa-miR-10a	81.0625	0.6498355	148.875	5.14332E-08	495.5625	3.678E-38	1081.813	3.678E-38
hsa-miR-10a*	552.625	3.678E-38	335.5625	3.678E-38	436.125	3.678E-38	465.9375	3.678E-38
hsa-miR-10b	114.0625	0.02352156	548.75	3.678E-38	915.9375	3.678E-38	2874.625	3.678E-38
hsa-miR-10b*	370.375	3.678E-38	186.125	2.44519E-10	174.875	0.00056768	123.25	0.01066916
hsa-miR-1178	342.8125	3.678E-38	1116.188	3.678E-38	109.1875	0.02565788	2253.5	3.678E-38
hsa-miR-1179	3716.5	3.678E-38	3547.125	3.678E-38	4951.875	3.678E-38	3451.188	3.678E-38
hsa-miR-1180	5747.75	3.678E-38	3471.438	3.678E-38	4005.188	3.678E-38	4333.063	3.678E-38
hsa-miR-1181	5564.125	3.678E-38	2965.25	3.678E-38	6386.813	3.678E-38	4109.063	3.678E-38
hsa-miR-1182	8137.875	3.678E-38	4069.313	3.678E-38	1087.563	3.678E-38	2323.375	3.678E-38
hsa-miR-1183	8729.438	3.678E-38	3904.063	3.678E-38	4632.688	3.678E-38	7428.688	3.678E-38
hsa-miR-1184	7615.125	3.678E-38	4294.5	3.678E-38	6250.688	3.678E-38	5877.375	3.678E-38
hsa-miR-1185	81.4375	0.7004322	74.75	0.8159098	75.375	0.7730961	74.5625	0.8308235
hsa-miR-1197	11614.75	3.678E-38	6422.688	3.678E-38	6455.25	3.678E-38	8189.313	3.678E-38
hsa-miR-1200	109.875	0.03301425	96	0.2977307	118.875	0.2009751	3208.313	3.678E-38
hsa-miR-1201	10273.81	3.678E-38	8854.313	3.678E-38	8853.813	3.678E-38	8773.438	3.678E-38
hsa-miR-1202	86.25	0.5307158	84.4375	0.5932302	80.25	0.7193592	81.625	0.6561642
hsa-miR-1203	81.375	0.6317145	96.875	0.2635376	84.625	0.6127641	93.375	0.3645097
hsa-miR-1204	510.875	3.678E-38	199.625	7.62398E-14	824.125	3.678E-38	283.25	9.40575E-28
hsa-miR-1205	147.375	3.04009E-05	106.625	0.1046249	119.6875	0.1162587	99.9375	0.2053237
hsa-miR-1206	298.875	3.678E-38	169.75	2.13394E-08	215.6875	2.0116E-08	168.875	5.40858E-07
hsa-miR-1207-3p	513.8125	3.678E-38	297.375	5.51615E-35	82.125	0.6934091	82.1875	0.6413895
hsa-miR-1207-5p	279.0625	3.678E-38	138.625	0.000170641	207.5	6.38132E-07	161.25	4.11873E-06
hsa-miR-1208	425.6875	3.678E-38	204	4.19962E-14	288.6875	1.21871E-14	270.9375	4.47295E-25
hsa-miR-122	3257.813	3.678E-38	472.6875	3.678E-38	3293	3.678E-38	210.125	5.55174E-19
hsa-miR-122*	116.0625	0.0158565	159	2.02189E-10	304.4375	3.678E-38	102.3125	0.1866364
hsa-miR-1224-3p	392.3125	3.678E-38	296	3.678E-38	257.125	2.81784E-14	322.6875	3.678E-38
hsa-miR-1224-5p	79.4375	0.6930854	96.125	0.1840062	111.4375	0.01758444	77.8125	0.7406024
hsa-miR-1225-3p	249.5	3.47244E-24	527.5625	3.678E-38	253.0625	2.49296E-13	543.375	3.678E-38
hsa-miR-1225-5p	10923.38	3.678E-38	4649.938	3.678E-38	7047.375	3.678E-38	8916.813	3.678E-38
hsa-miR-1226	1184.188	3.678E-38	230.1875	4.29403E-36	170.5	1.42273E-12	192	1.13138E-23
hsa-miR-1226*	342.9375	3.678E-38	82.3125	0.6366882	85.4375	0.5869688	147.125	3.39245E-09
hsa-miR-1227	81.875	0.6043637	108.375	0.02796568	122.125	0.00191239	120.625	0.00051743
hsa-miR-1228	168.0625	2.16728E-09	116.75	0.02319816	182.625	1.89135E-12	124.375	0.01313165
hsa-miR-1228*	4703.25	3.678E-38	2115.563	3.678E-38	2825.5	3.678E-38	2906.813	3.678E-38
hsa-miR-1229	132.375	9.24696E-05	184.125	1.5354E-11	114.0625	0.2181776	838.8125	3.678E-38
hsa-miR-1231	542.8125	3.678E-38	95.875	0.2555188	100.9375	0.2913327	96.9375	0.246512
hsa-miR-1233	724.6875	3.678E-38	351.75	3.678E-38	515.375	3.678E-38	423.5	3.678E-38
hsa-miR-1234	235.375	4.8984E-32	179.6875	5.8108E-09	342.25	3.678E-38	356.625	3.678E-38
hsa-miR-1236	78.75	0.6383524	76.625	0.7796841	75.625	0.8006009	75.875	0.7886119

hsa-miR-1237	78.75	0.7058659	242.9375	1.09461E-35	158.3125	2.19451E-10	91.6875	0.2881332
hsa-miR-1238	12564.81	3.678E-38	8553.5	3.678E-38	12774.88	3.678E-38	2329.438	3.678E-38
hsa-miR-124	91	0.3637354	79.625	0.7121041	80.375	0.7134566	79.875	0.6814215
hsa-miR-124*	385.875	3.678E-38	78.6875	0.7010459	73.875	0.8343497	72.9375	0.8398975
hsa-miR-1243	120.3125	0.004206743	102.8125	0.1417728	101.1875	0.2976385	104.125	0.1690426
hsa-miR-1244	82.625	0.6149766	81.5625	0.6496666	78.75	0.7215493	80.125	0.655521
hsa-miR-1245	592.875	3.678E-38	453.8125	3.678E-38	533.3125	3.678E-38	385.25	3.678E-38
hsa-miR-1246	18402.63	3.678E-38	13065.38	3.678E-38	10956.19	3.678E-38	13950.69	3.678E-38
hsa-miR-1247	92.8125	0.1798303	84.375	0.6061242	77.25	0.7632701	209.125	4.53693E-32
hsa-miR-1248	4780.813	3.678E-38	116	0.007315605	160.5625	3.97623E-09	108.1875	0.03092371
hsa-miR-1249	2262	3.678E-38	701.375	3.678E-38	791.6875	3.678E-38	439.5	3.678E-38
hsa-miR-124a:9.1	2443.125	3.678E-38	1124.063	3.678E-38	4162.875	3.678E-38	3682.25	3.678E-38
hsa-miR-1250	88.625	0.4198399	82.375	0.6422602	94.875	0.5013702	93	0.394146
hsa-miR-1251	79.0625	0.7451637	77.8125	0.7502731	77.25	0.7632701	75.8125	0.7818055
hsa-miR-1252	89.5	0.3586078	81.9375	0.6372975	84.4375	0.645225	83.0625	0.6171997
hsa-miR-1253	92.0625	0.3254347	84.4375	0.5939502	687.5	3.678E-38	85.8125	0.563794
hsa-miR-1254	3373.938	3.678E-38	2210.063	3.678E-38	2813.938	3.678E-38	893.625	3.678E-38
hsa-miR-1255a	80.9375	0.637516	78.9375	0.7294284	78.6875	0.7598366	157.125	5.18542E-12
hsa-miR-1255b	601.9375	3.678E-38	103.3125	0.1490474	162.125	0.005978484	152.8125	8.9974E-07
hsa-miR-1256	110.4375	0.1149774	97.4375	0.2411347	98.0625	0.3332339	109.1875	0.08539984
hsa-miR-1257	537.125	3.678E-38	297.75	3.678E-38	412.25	4.81615E-35	347.4375	3.678E-38
hsa-miR-1258	83.4375	0.4806634	79.625	0.7184024	76.9375	0.7853308	75.375	0.7937837
hsa-miR-1259	905.1875	3.678E-38	415.8125	3.678E-38	106	0.1185569	108.0625	0.09489509
hsa-miR-125a-3p	273.125	1.30419E-33	468	3.678E-38	1173.063	3.678E-38	606.875	3.678E-38
hsa-miR-125a-5p	3124.688	3.678E-38	13636.88	3.678E-38	14076.38	3.678E-38	9399.875	3.678E-38
hsa-miR-125b	3713.813	3.678E-38	18279.75	3.678E-38	16437.38	3.678E-38	17125.94	3.678E-38
hsa-miR-125b-1*	108.1875	0.05910943	548.5625	3.678E-38	104.9375	0.2455213	376.375	3.678E-38
hsa-miR-125b-2*	92.9375	0.3568549	1704.938	3.678E-38	768.625	3.678E-38	189.4375	1.80318E-22
hsa-miR-126	1749.25	3.678E-38	7268.75	3.678E-38	3959.75	3.678E-38	6972.375	3.678E-38
hsa-miR-126*	4331.938	3.678E-38	7264.313	3.678E-38	8475.75	3.678E-38	9940.625	3.678E-38
hsa-miR-1260	5800.438	3.678E-38	7464.625	3.678E-38	8470	3.678E-38	7850.75	3.678E-38
hsa-miR-1261	97.4375	0.1930088	87.625	0.4749479	95.5625	0.4238458	90.4375	0.4045297
hsa-miR-1262	423.5625	3.678E-38	326.6875	3.678E-38	334.0625	5.91724E-22	837.3125	3.678E-38
hsa-miR-1263	90	0.5228033	87.0625	0.4933038	89.1875	0.5380508	83.625	0.5978054
hsa-miR-1264	259.9375	3.678E-38	140.9375	0.000816965	135.375	0.06753941	200.5	1.25362E-09
hsa-miR-1265	81.75	0.6638741	87.1875	0.5450684	97.3125	0.3508957	86.875	0.5723708
hsa-miR-1266	73.75	0.7749178	74.8125	0.8149307	71.8125	0.8671242	73.75	0.834742
hsa-miR-1267	1776.25	3.678E-38	246.375	4.10604E-27	319.5	4.58743E-24	1359.25	3.678E-38
hsa-miR-1268	8648.5	3.678E-38	547	3.678E-38	1538.063	3.678E-38	3741.25	3.678E-38
hsa-miR-1269	142.4375	1.39522E-08	190.4375	1.21594E-19	164.5625	6.25097E-09	201.75	5.23209E-20
hsa-miR-1270	90.0625	0.4704913	124.625	0.000884636	90.5	0.4611928	98	0.1323979
hsa-miR-1271	2579.875	3.678E-38	1716.813	3.678E-38	482.875	3.678E-38	904.625	3.678E-38
hsa-miR-1272	177.75	1.71834E-08	126	0.004153182	174.5625	0.000328363	123.6875	0.01108353
hsa-miR-1273	252	3.678E-38	77.1875	0.7663658	77.9375	0.7785071	75.875	0.7916719
hsa-miR-127-3p	1763.438	3.678E-38	2403.75	3.678E-38	2592.625	3.678E-38	4211.625	3.678E-38
hsa-miR-1274a	722.125	3.678E-38	536.5	3.678E-38	943	3.678E-38	3920.188	3.678E-38
hsa-miR-1274b	10264.81	3.678E-38	9545.688	3.678E-38	11483.56	3.678E-38	8684.188	3.678E-38
hsa-miR-1275	9209.688	3.678E-38	4178.688	3.678E-38	6080.688	3.678E-38	537.75	3.678E-38
hsa-miR-127-5p	83.375	0.5280031	158.5	3.43372E-11	83.8125	0.5598892	101.375	0.2949673
hsa-miR-1276	146.4375	1.08276E-05	102.1875	0.1617234	140.1875	0.03217294	104.8125	0.1277553
hsa-miR-1277	77.75	0.750639	1078.063	3.678E-38	73.5625	0.8294402	909.1875	3.678E-38
hsa-miR-1278	90.375	0.3066338	99.375	0.1388172	84.75	0.6388793	83.5625	0.6113287
hsa-miR-1279	100.25	0.1598232	88.1875	0.4928409	99.1875	0.4327705	130.0625	4.24071E-05
hsa-miR-128	18279.75	3.678E-38	7773.813	3.678E-38	6667.188	3.678E-38	9439.438	3.678E-38
hsa-miR-1280	8716.063	3.678E-38	7769.313	3.678E-38	9446.5	3.678E-38	10025.81	3.678E-38
hsa-miR-1281	134.4375	0.01462607	87.375	0.4904197	88.3125	0.5178638	88.6875	0.4780149
hsa-miR-1282	4194.688	3.678E-38	145.5	3.39263E-07	98.5	0.2905475	166.5625	2.5062E-13
hsa-miR-1283	147.8125	8.02236E-08	130.4375	0.002276589	139.5	0.02481225	140.75	0.000978168
hsa-miR-1284	780.8125	3.678E-38	344.75	3.678E-38	569.8125	3.678E-38	450.4375	3.678E-38
hsa-miR-1285	88.625	0.5519757	135.25	5.59131E-06	626.6875	3.678E-38	76.8125	0.7621026
hsa-miR-1286	390.875	3.678E-38	1331.438	3.678E-38	263	2.99518E-14	257.9375	1.19057E-22
hsa-miR-1287	100.1875	0.14753	139.6875	1.27681E-06	96.375	0.3599887	111.0625	0.02077616
hsa-miR-1288	185.75	1.2559E-13	152	3.13809E-05	161.625	0.00195417	135.875	0.001869846
hsa-miR-1289	236.9375	1.56491E-20	1189.438	3.678E-38	232.875	8.80318E-09	187.125	2.17353E-08
hsa-miR-128a:9.1	6979.375	3.678E-38	1253.875	3.678E-38	1012.688	3.678E-38	1898.125	3.678E-38
hsa-miR-128b:9.1	4609.5	3.678E-38	1165.875	3.678E-38	514.1875	3.678E-38	1617.563	3.678E-38
hsa-miR-129*	105.0625	0.0527707	132.6875	6.54318E-05	189.5625	2.64717E-16	216.375	2.67784E-27
hsa-miR-1290	957.1875	3.678E-38	394.9375	3.678E-38	713.5625	3.678E-38	1994.188	3.678E-38
hsa-miR-1291	81.25	0.5854855	128.9375	0.00067426	114	0.1638122	158.625	4.10004E-11
hsa-miR-1292	76.0625	0.7443895	77	0.7725378	78.5625	0.7464678	76.4375	0.773719
hsa-miR-1293	92.8125	0.2343605	86.75	0.5515761	84.25	0.6326246	83.5625	0.6204578
hsa-miR-129-3p	75.625	0.7763035	565.1875	3.678E-38	1174.125	3.678E-38	211.5625	1.39522E-33
hsa-miR-1294	227.3125	6.56099E-31	140.5	0.000231927	178.3125	0.000134124	140.625	0.000431236
hsa-miR-1295	442.8125	3.678E-38	205.25	5.48162E-18	359.6875	3.678E-38	266.8125	4.20303E-31
hsa-miR-129-5p	3097.563	3.678E-38	1036.5	3.678E-38	1362.125	3.678E-38	646.75	3.678E-38
hsa-miR-1296	403.6875	3.678E-38	1568.438	3.678E-38	437.5625	3.678E-38	2355.25	3.678E-38
hsa-miR-1297	114.25	0.01494711	181.625	1.08364E-16	136.5	0.000119387	95.9375	0.2588681
hsa-miR-1298	171.625	1.28887E-08	123.5625	0.008502581	139.5	0.01878832	173.0625	8.27824E-14
hsa-miR-1299	2734.188	3.678E-38	450.75	3.678E-38	2588.813	3.678E-38	1197.125	3.678E-38
hsa-miR-1300	597.5	3.678E-38	223.25	4.22997E-21	189.3125	0.000259742	282.5625	3.678E-38

hsa-miR-1301	1533.625	3.678E-38	2493.875	3.678E-38	2698.75	3.678E-38	901.5	3.678E-38
hsa-miR-1302	4175.125	3.678E-38	252.4375	3.678E-38	79.375	0.7020159	139.8125	1.5137E-07
hsa-miR-1303	2972.25	3.678E-38	482.75	3.678E-38	845.5	3.678E-38	93.1875	0.2023452
hsa-miR-1304	9632	3.678E-38	1788.5	3.678E-38	6681.563	3.678E-38	7328.813	3.678E-38
hsa-miR-1305	433.625	3.678E-38	211	2.92852E-16	306.5	1.01274E-20	248.5625	1.83349E-20
hsa-miR-1307	109.9375	0.1268887	205.1875	1.31137E-25	589.3125	3.678E-38	961.1875	3.678E-38
hsa-miR-1308	9354.188	3.678E-38	6615.875	3.678E-38	8035.75	3.678E-38	8514.875	3.678E-38
hsa-miR-130a	5815.188	3.678E-38	14173.25	3.678E-38	10330.44	3.678E-38	7282.188	3.678E-38
hsa-miR-130a*	1341.625	3.678E-38	1026.625	3.678E-38	386.875	1.06578E-37	610.375	3.678E-38
hsa-miR-130b	2045.5	3.678E-38	1560	3.678E-38	218.8125	2.51471E-22	1262.313	3.678E-38
hsa-miR-130b*	7464.625	3.678E-38	2751.938	3.678E-38	3989.438	3.678E-38	3705.813	3.678E-38
hsa-miR-132	6758.5	3.678E-38	8216.875	3.678E-38	10459	3.678E-38	7844.5	3.678E-38
hsa-miR-132*	385.8125	3.678E-38	1829.625	3.678E-38	1400.938	3.678E-38	2165.5	3.678E-38
hsa-miR-1321	85.875	0.5458829	82.75	0.6164106	82.5	0.6566842	85.875	0.5320161
hsa-miR-1322	105.3125	0.1990752	224.5	7.60856E-18	275.3125	8.36341E-13	282.625	7.50226E-28
hsa-miR-1323	220.875	2.00741E-25	151.75	2.10976E-05	195.4375	2.20178E-05	139.6875	0.000822498
hsa-miR-1324	119.3125	0.008198172	82.25	0.6456898	285.6875	6.66297E-11	111.5625	0.07162447
hsa-miR-133a	835.25	3.678E-38	5582	3.678E-38	855	3.678E-38	1021.125	3.678E-38
hsa-miR-133b	81.125	0.6350334	2842.375	3.678E-38	96.6875	0.1993816	727.0625	3.678E-38
hsa-miR-134	106.6875	0.03686821	2146.438	3.678E-38	2288.25	3.678E-38	3406	3.678E-38
hsa-miR-135a	1826.375	3.678E-38	6352.438	3.678E-38	3907.063	3.678E-38	5980.5	3.678E-38
hsa-miR-135a*	100.125	0.1218924	221.75	1.04977E-32	88.1875	0.5941623	86.0625	0.5313109
hsa-miR-135b	93.4375	0.3037892	518.375	3.678E-38	3300.25	3.678E-38	863.875	3.678E-38
hsa-miR-135b*	92.75	0.4411692	191.125	7.01705E-12	120.75	0.003566159	150.8125	1.20984E-05
hsa-miR-136	87.3125	0.479278	5950.188	3.678E-38	2775.188	3.678E-38	4244.5	3.678E-38
hsa-miR-136*	78.4375	0.7415378	2225.75	3.678E-38	234	6.5744E-38	405.3125	3.678E-38
hsa-miR-137	273	3.678E-38	163.1875	9.51299E-07	229.8125	4.5633E-08	196.125	2.32889E-10
hsa-miR-138	151.0625	0.00019659	170.5625	2.89639E-08	332.0625	3.678E-38	165.8125	2.51992E-06
hsa-miR-138-1*	171.375	2.05488E-10	108.6875	0.0889651	150.5	0.01532776	131.8125	0.006948203
hsa-miR-138-2*	148.6875	8.31419E-08	115.125	0.03728933	107.25	0.2037303	147.0625	4.64268E-07
hsa-miR-139-3p	92.6875	0.2108739	152.75	2.30062E-09	132	5.00635E-05	265.125	3.678E-38
hsa-miR-139-5p	155.875	1.21298E-08	1147.875	3.678E-38	1899.313	3.678E-38	2693.563	3.678E-38
hsa-miR-140-3p	4213.438	3.678E-38	920.1875	3.678E-38	2821.75	3.678E-38	3343.125	3.678E-38
hsa-miR-140-5p	2397.875	3.678E-38	2239.563	3.678E-38	268.8125	3.678E-38	2339.25	3.678E-38
hsa-miR-141	88.9375	0.4979153	8182.5	3.678E-38	7019.625	3.678E-38	8176.813	3.678E-38
hsa-miR-141*	75.625	0.7623823	1138.75	3.678E-38	81.5	0.6319076	77.625	0.7385918
hsa-miR-142-3p	14427.44	3.678E-38	5420.813	3.678E-38	5094.625	3.678E-38	7440.5	3.678E-38
hsa-miR-142-5p	7784.75	3.678E-38	1470.875	3.678E-38	130.9375	0.000128182	227.125	3.678E-38
hsa-miR-143	768.25	3.678E-38	9245.75	3.678E-38	6010.375	3.678E-38	5243.75	3.678E-38
hsa-miR-143*	1025.75	3.678E-38	464	3.678E-38	805.375	3.678E-38	572.4375	3.678E-38
hsa-miR-144	165.125	6.2541E-11	103.0625	0.1368666	175.1875	0.001907953	136.6875	0.004031396
hsa-miR-144*	373.4375	1.56303E-23	268.125	3.678E-38	169.625	5.31092E-13	260.75	3.678E-38
hsa-miR-144:9.1	464.9375	3.678E-38	212.25	1.83778E-14	379.1875	1.20142E-23	377.6875	3.678E-38
hsa-miR-145	1037.125	3.678E-38	7809.5	3.678E-38	6204.313	3.678E-38	6283.813	3.678E-38
hsa-miR-145*	111	0.04427445	2437.438	3.678E-38	725.4375	3.678E-38	1330.813	3.678E-38
hsa-miR-1468	89.875	0.2406529	173.8125	1.59408E-15	74.9375	0.816759	73.3125	0.853501
hsa-miR-146a	13490.56	3.678E-38	7448.313	3.678E-38	7775.188	3.678E-38	10345.38	3.678E-38
hsa-miR-146a*	104.25	0.04319394	85.5	0.5549398	88.6875	0.5603859	86.75	0.5413876
hsa-miR-146b-3p	143.875	3.56709E-06	114.5625	0.0271491	110.875	0.1001634	116.9375	0.04352759
hsa-miR-146b-5p	6846.938	3.678E-38	10654.88	3.678E-38	12641.06	3.678E-38	10743.81	3.678E-38
hsa-miR-147	207.8125	1.72739E-19	123.625	0.01090218	178.625	0.001408941	171.3125	5.27991E-06
hsa-miR-147b	395.75	3.678E-38	277.875	3.678E-38	315.9375	1.60655E-20	246.875	9.62679E-21
hsa-miR-148a	6636.438	3.678E-38	9915.5	3.678E-38	8482.813	3.678E-38	12910.31	3.678E-38
hsa-miR-148a*	109.1875	0.02862577	92.625	0.3824053	110.1875	0.3025339	89.0625	0.4512851
hsa-miR-148b	89.3125	0.4893197	2085.813	3.678E-38	2040.813	3.678E-38	2049.75	3.678E-38
hsa-miR-148b*	169.875	7.59266E-12	209.75	2.45577E-23	142.625	0.01831054	129.375	0.007461898
hsa-miR-149	90.1875	0.5614424	6495.063	3.678E-38	4853.938	3.678E-38	4375.125	3.678E-38
hsa-miR-149*	91.625	0.3901337	179.3125	1.43553E-16	185.875	7.82856E-17	87.75	0.4974319
hsa-miR-150	23846.44	3.678E-38	8646.375	3.678E-38	5316.625	3.678E-38	8874.875	3.678E-38
hsa-miR-150*	6075.438	3.678E-38	574.3125	3.678E-38	677.6875	3.678E-38	491.875	3.678E-38
hsa-miR-151:9.1	1791.75	3.678E-38	6508.688	3.678E-38	5765.875	3.678E-38	5578.375	3.678E-38
hsa-miR-151-3p	2840.938	3.678E-38	5867.75	3.678E-38	6280.75	3.678E-38	6311.75	3.678E-38
hsa-miR-151-5p	4343.125	3.678E-38	12184.94	3.678E-38	8980.25	3.678E-38	9644.625	3.678E-38
hsa-miR-152	101.3125	0.1733287	9605.125	3.678E-38	5672	3.678E-38	6495	3.678E-38
hsa-miR-153	93	0.371659	183.5	8.02785E-18	110.8125	0.03566539	149.5625	1.22153E-09
hsa-miR-1537	92.6875	0.314576	84.875	0.5518519	87.6875	0.5747174	270	3.678E-38
hsa-miR-154	89.3125	0.3926839	2800.75	3.678E-38	116.9375	0.03435928	2927.563	3.678E-38
hsa-miR-154*	93.125	0.3590479	1364	3.678E-38	152.5625	0.00828931	345.375	3.678E-38
hsa-miR-155	10287.63	3.678E-38	4735.125	3.678E-38	6136.5	3.678E-38	7325.688	3.678E-38
hsa-miR-155*	96.0625	0.2585801	100.6875	0.1262643	129.3125	0.00064087	124.625	0.000403237
hsa-miR-15a	12019.63	3.678E-38	7880.438	3.678E-38	6984.125	3.678E-38	5238.063	3.678E-38
hsa-miR-15a*	2204.813	3.678E-38	727.3125	3.678E-38	772.375	3.678E-38	82.625	0.6318638
hsa-miR-15b	18801.63	3.678E-38	12079.06	3.678E-38	15051.63	3.678E-38	13738.5	3.678E-38
hsa-miR-15b*	4919.688	3.678E-38	468.375	3.678E-38	1970.25	3.678E-38	1306.688	3.678E-38
hsa-miR-16	15392.31	3.678E-38	10221.31	3.678E-38	12432.06	3.678E-38	11524.25	3.678E-38
hsa-miR-16-1*	3097.375	3.678E-38	226.9375	4.42866E-19	814.5625	3.678E-38	489.5625	3.678E-38
hsa-miR-16-2*	10085.75	3.678E-38	4621.75	3.678E-38	3847.188	3.678E-38	3503.938	3.678E-38
hsa-miR-17	11500.25	3.678E-38	8074.063	3.678E-38	8055.813	3.678E-38	4628.5	3.678E-38
hsa-miR-17*	282.8125	1.31941E-12	1080.25	3.678E-38	1229.563	3.678E-38	234.8125	3.678E-38
hsa-miR-17-5p:9.1	99.875	0.4570192	328.5	3.678E-38	312.5	1.17706E-28	197.375	1.28568E-25

hsa-miR-181a	21741.38	3.678E-38	14368.19	3.678E-38	10879.63	3.678E-38	14860.94	3.678E-38
hsa-miR-181a*	877.625	3.678E-38	814.5	3.678E-38	569.3125	3.678E-38	1682.938	3.678E-38
hsa-miR-181a-2*	4471.063	3.678E-38	2072.938	3.678E-38	1133.125	3.678E-38	1367	3.678E-38
hsa-miR-181b	15828.25	3.678E-38	7903.063	3.678E-38	6354.188	3.678E-38	7491.813	3.678E-38
hsa-miR-181c	2494.375	3.678E-38	2825.25	3.678E-38	1705	3.678E-38	4850	3.678E-38
hsa-miR-181c*	2402.125	3.678E-38	331.8125	3.678E-38	113.625	0.1273077	302.625	3.678E-38
hsa-miR-181d	87.1875	0.4545599	158.125	2.088E-10	81.875	0.6798633	85.4375	0.5683239
hsa-miR-182	3825.438	3.678E-38	10475.25	3.678E-38	10021.69	3.678E-38	4938.188	3.678E-38
hsa-miR-182*	838.375	3.678E-38	1629	3.678E-38	2438.938	3.678E-38	1290.688	3.678E-38
hsa-miR-1825	103.4375	0.08408213	93.4375	0.3233747	108.0625	0.2299441	113.625	0.06899679
hsa-miR-1826	6523.313	3.678E-38	5594	3.678E-38	6132.063	3.678E-38	6467.125	3.678E-38
hsa-miR-1827	83.5625	0.5534536	86.4375	0.5153587	82	0.6663874	81.1875	0.6576855
hsa-miR-183	264.75	1.05808E-09	13296.81	3.678E-38	13449.69	3.678E-38	8914.375	3.678E-38
hsa-miR-183*	128.875	0.000140631	1040.688	3.678E-38	997.5625	3.678E-38	275.9375	3.678E-38
hsa-miR-184	77.125	0.7457214	430.6875	3.678E-38	1315.25	3.678E-38	555.125	3.678E-38
hsa-miR-185	2513.875	3.678E-38	725.875	3.678E-38	702.875	3.678E-38	625.5625	3.678E-38
hsa-miR-185*	81.5	0.6284788	112.6875	0.00937084	150.9375	3.43594E-06	85.625	0.5276745
hsa-miR-186	171.8125	1.04928E-08	2671.625	3.678E-38	351.625	3.678E-38	1886	3.678E-38
hsa-miR-186*	228.125	1.18441E-27	116.0625	0.03412903	194.25	1.06874E-06	150.4375	0.000213376
hsa-miR-187	2919.375	3.678E-38	125.625	0.000443021	2014.75	3.678E-38	85.125	0.5453759
hsa-miR-187*	5770.188	3.678E-38	1994.188	3.678E-38	3425.063	3.678E-38	4019.688	3.678E-38
hsa-miR-188-3p	121.3125	0.000710134	91.4375	0.4128981	93	0.4468014	95.5	0.2706544
hsa-miR-188-5p	797.625	3.678E-38	425.125	3.678E-38	705.8125	3.678E-38	532.875	3.678E-38
hsa-miR-189:9.1	2979.813	3.678E-38	3531.438	3.678E-38	4776.063	3.678E-38	3386.688	3.678E-38
hsa-miR-18a	3086.438	3.678E-38	4156.813	3.678E-38	3288.938	3.678E-38	4114.625	3.678E-38
hsa-miR-18a*	1154.5	3.678E-38	396.8125	3.678E-38	374.25	1.74748E-23	525.0625	3.678E-38
hsa-miR-18b	126.5625	0.000482169	198.0625	7.67135E-22	200.1875	1.14543E-18	113.9375	0.03139401
hsa-miR-18b*	5332.375	3.678E-38	1931.938	3.678E-38	3204	3.678E-38	2063.063	3.678E-38
hsa-miR-190	839.5	3.678E-38	235.375	1.87512E-30	238.875	2.41465E-10	320.5	3.678E-38
hsa-miR-190b	81.8125	0.6147934	202.125	5.0766E-25	269.875	3.678E-38	82.375	0.5900315
hsa-miR-191	13515.44	3.678E-38	13404.94	3.678E-38	12319.69	3.678E-38	12997.88	3.678E-38
hsa-miR-191*	90.5625	0.4453375	179.4375	6.58563E-10	104.375	0.1969113	117.5	0.02173369
hsa-miR-192	1863.188	3.678E-38	3137.688	3.678E-38	3107.563	3.678E-38	3262.5	3.678E-38
hsa-miR-192*	163.375	2.93464E-15	217.4375	4.98326E-24	83.3125	0.6318113	186.3125	2.81486E-12
hsa-miR-193a-3p	632.375	3.678E-38	978.3125	3.678E-38	742.0625	3.678E-38	634.625	3.678E-38
hsa-miR-193a-5p	2603.25	3.678E-38	5615.5	3.678E-38	3523.375	3.678E-38	3327.188	3.678E-38
hsa-miR-193b	4932.375	3.678E-38	10723.31	3.678E-38	6281.875	3.678E-38	9353.563	3.678E-38
hsa-miR-193b*	1999.813	3.678E-38	1441.875	3.678E-38	1716.188	3.678E-38	792.25	3.678E-38
hsa-miR-194	99.625	0.1642092	8775.313	3.678E-38	3763.375	3.678E-38	3969.188	3.678E-38
hsa-miR-194*	593.5625	3.678E-38	269.875	2.97395E-34	446.9375	3.678E-38	361.9375	3.678E-38
hsa-miR-195	2875.688	3.678E-38	15771.5	3.678E-38	15489.19	3.678E-38	15143.5	3.678E-38
hsa-miR-195*	91	0.3874152	479.75	3.678E-38	89.25	0.4657676	197.25	4.49527E-26
hsa-miR-196a	4130.75	3.678E-38	149.625	1.17693E-07	2699.813	3.678E-38	1592.563	3.678E-38
hsa-miR-196a*	79.4375	0.6556301	82.9375	0.6499814	82.5625	0.7215225	86.75	0.5935575
hsa-miR-196b	6757.125	3.678E-38	540.3125	3.678E-38	431.4375	3.678E-38	1104.563	3.678E-38
hsa-miR-197	13707.38	3.678E-38	9164.563	3.678E-38	10238.44	3.678E-38	10316.56	3.678E-38
hsa-miR-198	104.4375	0.08250433	93.1875	0.348355	1865.063	3.678E-38	97.25	0.2983574
hsa-miR-199a*:9.1	4327.5	3.678E-38	2965.625	3.678E-38	4220.375	3.678E-38	8812.125	3.678E-38
hsa-miR-199a-3p,hsa-	6136.688	3.678E-38	9775.938	3.678E-38	7308.75	3.678E-38	9445.938	3.678E-38
hsa-miR-199a-5p	77.8125	0.7378854	2026	3.678E-38	2163.625	3.678E-38	2742.063	3.678E-38
hsa-miR-199b-5p	113.75	0.0255929	2483.813	3.678E-38	615.75	3.678E-38	3048.563	3.678E-38
hsa-miR-19a	919.625	3.678E-38	1291.938	3.678E-38	281.125	3.678E-38	1598.688	3.678E-38
hsa-miR-19a*	84.5625	0.5707178	81.0625	0.6659129	88.1875	0.5747346	84.8125	0.5882956
hsa-miR-19b	9961.188	3.678E-38	3336.25	3.678E-38	2946.563	3.678E-38	1514.5	3.678E-38
hsa-miR-19b-1*	91.6875	0.3671383	90.3125	0.3797388	82.625	0.6278687	83.875	0.582436
hsa-miR-19b-2*	2310.938	3.678E-38	181.0625	2.56521E-11	231.6875	3.05265E-09	1129.563	3.678E-38
hsa-miR-200a	123.25	0.1014032	14509.13	3.678E-38	14299.06	3.678E-38	13286.75	3.678E-38
hsa-miR-200a*	207.25	2.97434E-26	2186.438	3.678E-38	4057.25	3.678E-38	2417.188	3.678E-38
hsa-miR-200b	8781.875	3.678E-38	20707.81	3.678E-38	22182.25	3.678E-38	20361.25	3.678E-38
hsa-miR-200b*	101.25	0.1665166	8097.25	3.678E-38	10972.44	3.678E-38	8192.563	3.678E-38
hsa-miR-200c	13161.63	3.678E-38	22858.81	3.678E-38	24922.06	3.678E-38	23846.44	3.678E-38
hsa-miR-200c*	80.125	0.6589559	222.1875	7.20595E-34	260.625	3.19684E-08	403.3125	3.678E-38
hsa-miR-202	105.625	0.05287544	104.75	0.1342649	225.4375	3.51488E-30	92.625	0.3752072
hsa-miR-202*	325.375	1.95567E-32	89	0.449491	110.125	0.07997388	118.6875	0.02749919
hsa-miR-202*:9.1	14170.56	3.678E-38	7167.75	3.678E-38	9778.75	3.678E-38	9174.313	3.678E-38
hsa-miR-203	636.4375	3.678E-38	4092.313	3.678E-38	5811.188	3.678E-38	7781.875	3.678E-38
hsa-miR-204	1283.875	3.678E-38	7878.688	3.678E-38	6319.063	3.678E-38	7763.375	3.678E-38
hsa-miR-205	300.8125	5.04403E-27	17239.19	3.678E-38	16167.56	3.678E-38	16008.19	3.678E-38
hsa-miR-206	5939	3.678E-38	16406.13	3.678E-38	9835.813	3.678E-38	9708.813	3.678E-38
hsa-miR-208a	2357.813	3.678E-38	88.5	0.4317974	83.3125	0.7108539	83.1875	0.6536548
hsa-miR-208b	115	0.006863985	98.375	0.2355663	103.75	0.3027711	95.6875	0.3016486
hsa-miR-20a	13700.06	3.678E-38	11895	3.678E-38	10703.06	3.678E-38	7800.438	3.678E-38
hsa-miR-20a*	1414.313	3.678E-38	1759.25	3.678E-38	500.0625	3.678E-38	1316.063	3.678E-38
hsa-miR-20b	8706.25	3.678E-38	4686.688	3.678E-38	1398	3.678E-38	2061.625	3.678E-38
hsa-miR-20b*	530	3.678E-38	90.8125	0.4287134	90	0.5379143	86.25	0.5327177
hsa-miR-21	19955.81	3.678E-38	21755.63	3.678E-38	22668	3.678E-38	21998.5	3.678E-38
hsa-miR-21*	83.4375	0.5748532	230.6875	9.95815E-36	203.125	9.88933E-16	169.3125	1.72603E-14
hsa-miR-210	78.875	0.7227547	856.0625	3.678E-38	267.1875	3.678E-38	592.6875	3.678E-38
hsa-miR-211	141.3125	5.06513E-05	425.1875	3.678E-38	419.875	3.678E-38	142.6875	0.001345813
hsa-miR-212	394.6875	6.14542E-28	2143.125	3.678E-38	1998.125	3.678E-38	3136.625	3.678E-38

hsa-miR-214	1084.25	3.678E-38	9896	3.678E-38	9169.438	3.678E-38	11286.25	3.678E-38
hsa-miR-214*	100.9375	0.1170092	105.6875	0.0738957	96.4375	0.4281082	102.3125	0.2149246
hsa-miR-215	106.25	0.07262647	157	1.82656E-09	446.1875	3.678E-38	184.4375	1.58339E-18
hsa-miR-216a	798.0625	3.678E-38	173.5625	1.19441E-13	356.0625	3.678E-38	511.1875	3.678E-38
hsa-miR-216b	538.5	3.678E-38	364.6875	3.678E-38	464	3.678E-38	367.0625	3.678E-38
hsa-miR-217	86.0625	0.5151163	3129.563	3.678E-38	121.375	0.002681507	93	0.2341402
hsa-miR-218	3783.875	3.678E-38	5438.688	3.678E-38	8536	3.678E-38	9257	3.678E-38
hsa-miR-218-1*	463.9375	3.678E-38	288.5	3.678E-38	424.6875	3.678E-38	284.1875	2.57265E-31
hsa-miR-218-2*	81.6875	0.621236	80.1875	0.6916378	95.75	0.2297413	79.75	0.6996224
hsa-miR-219-1-3p	83.4375	0.5634059	84.1875	0.5721632	86.6875	0.6018209	86.125	0.5699428
hsa-miR-219-2-3p	215.625	5.92866E-29	95.4375	0.262642	106.4375	0.2291451	138.8125	0.001681695
hsa-miR-219-5p	4115.75	3.678E-38	103.25	0.1660136	4970.125	3.678E-38	3518.375	3.678E-38
hsa-miR-22	85.0625	0.5703307	5024.313	3.678E-38	3978.75	3.678E-38	7002.063	3.678E-38
hsa-miR-22*	84.875	0.5052612	2446	3.678E-38	3558.375	3.678E-38	3231.375	3.678E-38
hsa-miR-220a	6152.313	3.678E-38	93.25	0.3127415	111	0.2128749	3989.125	3.678E-38
hsa-miR-220b	200	1.55848E-07	154.4375	3.98998E-05	187	2.10127E-16	97.1875	0.2688905
hsa-miR-220c	87.8125	0.492159	95.125	0.2905101	93	0.415482	97.875	0.2538233
hsa-miR-221	9436.563	3.678E-38	12149.56	3.678E-38	12016.44	3.678E-38	12616.69	3.678E-38
hsa-miR-221*	85.125	0.5315979	1928.75	3.678E-38	1001.5	3.678E-38	295.875	3.678E-38
hsa-miR-222	267.625	1.82379E-22	6749.438	3.678E-38	5321.25	3.678E-38	4631.188	3.678E-38
hsa-miR-222*	289.9375	3.678E-38	137.375	0.000411426	190.1875	6.71361E-06	200.75	5.92701E-11
hsa-miR-223	6325.688	3.678E-38	5785.688	3.678E-38	7145.438	3.678E-38	9259.188	3.678E-38
hsa-miR-223*	106.5625	0.1446511	187.3125	7.44174E-18	195.3125	7.01678E-20	196.625	5.99496E-25
hsa-miR-224	74.75	0.7842025	2029.75	3.678E-38	2429.875	3.678E-38	2526.313	3.678E-38
hsa-miR-23a	2630.875	3.678E-38	10541.38	3.678E-38	13021.25	3.678E-38	12857.63	3.678E-38
hsa-miR-23a*	266.5625	3.678E-38	158.5	6.98887E-08	384.75	3.678E-38	2042.188	3.678E-38
hsa-miR-23b	10541.94	3.678E-38	17346.31	3.678E-38	18193.5	3.678E-38	16567.38	3.678E-38
hsa-miR-23b*	234.75	7.37287E-11	258.5	3.678E-38	635.5625	3.678E-38	210.5625	1.54203E-29
hsa-miR-24	6309.438	3.678E-38	15088.75	3.678E-38	15833	3.678E-38	14735.5	3.678E-38
hsa-miR-24-1*	88.875	0.4099135	1107.813	3.678E-38	253.6875	3.678E-38	597.75	3.678E-38
hsa-miR-24-2*	1352.813	3.678E-38	2147.5	3.678E-38	2534.375	3.678E-38	2331.875	3.678E-38
hsa-miR-25	15917.56	3.678E-38	9476.5	3.678E-38	12184.94	3.678E-38	9441.938	3.678E-38
hsa-miR-25*	1354.375	3.678E-38	213.75	1.49584E-28	204.6875	4.87827E-19	423.3125	3.678E-38
hsa-miR-26a	20450.69	3.678E-38	20299.56	3.678E-38	19740.06	3.678E-38	19808.19	3.678E-38
hsa-miR-26a-1*	71.8125	0.848863	374.8125	3.678E-38	79.8125	0.7708301	89.5	0.313821
hsa-miR-26a-2*	104.875	0.07242623	228.875	1.20043E-36	167.25	4.71735E-11	295.0625	3.678E-38
hsa-miR-26b	19740.06	3.678E-38	19935.5	3.678E-38	19784.38	3.678E-38	19935.5	3.678E-38
hsa-miR-26b*	1034.313	3.678E-38	388.125	3.678E-38	303.125	3.678E-38	334.375	3.678E-38
hsa-miR-27a	2171.25	3.678E-38	9423	3.678E-38	8431.813	3.678E-38	9450.938	3.678E-38
hsa-miR-27a*	374.4375	3.678E-38	465.3125	3.678E-38	507.5625	3.678E-38	596.125	3.678E-38
hsa-miR-27b	2079.5	3.678E-38	10079	3.678E-38	6333.063	3.678E-38	7517.75	3.678E-38
hsa-miR-27b*	414.25	3.678E-38	1105.688	3.678E-38	546.3125	3.678E-38	308.5	3.678E-38
hsa-miR-28-3p	3202.25	3.678E-38	2807.75	3.678E-38	3598.688	3.678E-38	2575.063	3.678E-38
hsa-miR-28-5p	9786.188	3.678E-38	8398.75	3.678E-38	4479.063	3.678E-38	5520.813	3.678E-38
hsa-miR-296-3p	82.75	0.5908133	235.375	3.678E-38	157.625	1.13173E-09	79.75	0.6996224
hsa-miR-296-5p	796.6875	3.678E-38	917.5	3.678E-38	275.875	3.678E-38	308.375	3.678E-38
hsa-miR-297	2107.938	3.678E-38	86.125	0.5271115	87.125	0.5624595	87.9375	0.4723803
hsa-miR-298	439.9375	3.678E-38	262.5	2.22236E-28	393.1875	1.57546E-30	268.75	1.78557E-25
hsa-miR-299-3p	113.6875	0.01178631	646.0625	3.678E-38	121.9375	0.01943192	104.5	0.154513
hsa-miR-299-5p	1482.125	3.678E-38	2478.313	3.678E-38	494.25	3.678E-38	1521.438	3.678E-38
hsa-miR-29a	1366.938	3.678E-38	7074.625	3.678E-38	9110.063	3.678E-38	8464.75	3.678E-38
hsa-miR-29a*	311.25	3.678E-38	3086.813	3.678E-38	543.75	3.678E-38	911.875	3.678E-38
hsa-miR-29b	3234	3.678E-38	12825.31	3.678E-38	13680.5	3.678E-38	17346.31	3.678E-38
hsa-miR-29b-1*	635.8125	3.678E-38	5724.875	3.678E-38	4371.625	3.678E-38	4337	3.678E-38
hsa-miR-29b-2*	901.0625	3.678E-38	625.8125	3.678E-38	372.8125	3.678E-38	1401.375	3.678E-38
hsa-miR-29c	2143.25	3.678E-38	5880.375	3.678E-38	5240.063	3.678E-38	7564.625	3.678E-38
hsa-miR-29c*	1109.313	3.678E-38	2542.063	3.678E-38	1855.25	3.678E-38	2427.875	3.678E-38
hsa-miR-300	296.25	3.678E-38	252.375	2.07472E-33	213.75	3.41127E-06	179.9375	1.23455E-08
hsa-miR-301a	655.5625	3.678E-38	2927.875	3.678E-38	619.4375	3.678E-38	719.1875	3.678E-38
hsa-miR-301b	78.875	0.6839137	75.375	0.8080075	77.6875	0.7618705	76.0625	0.7888671
hsa-miR-302a	82.625	0.5451232	111.875	0.01468346	243.375	3.678E-38	109.6875	0.01046865
hsa-miR-302a*	168.0625	1.45752E-06	267.5625	3.678E-38	177	1.4977E-12	99	0.1857802
hsa-miR-302b	90.8125	0.3248235	89.75	0.4474716	3508.438	3.678E-38	279.6875	3.678E-38
hsa-miR-302b*	5835.5	3.678E-38	2664.938	3.678E-38	3678.688	3.678E-38	2796.25	3.678E-38
hsa-miR-302c	86.3125	0.4338702	101.125	0.1969492	108.375	0.2676813	900.5	3.678E-38
hsa-miR-302c*	282.3125	3.678E-38	203.0625	1.44567E-13	218	7.82932E-07	255.0625	8.0549E-21
hsa-miR-302d	13645	3.678E-38	6185.188	3.678E-38	6806.25	3.678E-38	7230.063	3.678E-38
hsa-miR-302d*	89.4375	0.4086739	85.25	0.5695062	80.125	0.7060559	84.75	0.5719205
hsa-miR-302e	78.8125	0.6787576	75.625	0.7977502	77.25	0.7919448	76.6875	0.7793591
hsa-miR-302f	72.375	0.8308743	72.1875	0.8692552	73.6875	0.8171703	72.4375	0.8589861
hsa-miR-30a	819.75	3.678E-38	4966.813	3.678E-38	7091.375	3.678E-38	6646.438	3.678E-38
hsa-miR-30a*	77.3125	0.6974667	262	3.678E-38	2079.625	3.678E-38	1036	3.678E-38
hsa-miR-30b	6826	3.678E-38	10288.56	3.678E-38	8397.563	3.678E-38	8146.75	3.678E-38
hsa-miR-30b*	220.625	6.76721E-33	203.625	1.73021E-12	183.75	0.000390683	128	0.01090228
hsa-miR-30c	12016.63	3.678E-38	16338.25	3.678E-38	14078.5	3.678E-38	14963.25	3.678E-38
hsa-miR-30c-1*	2041.375	3.678E-38	82	0.654568	166.0625	2.36497E-11	158.75	2.75486E-11
hsa-miR-30c-2*	125.375	0.001094727	97.6875	0.2481792	225.875	1.22813E-15	257.875	3.678E-38
hsa-miR-30d	9257	3.678E-38	18577.69	3.678E-38	13477.31	3.678E-38	15465.81	3.678E-38
hsa-miR-30d*	1065.938	3.678E-38	272.8125	1.43095E-32	359.6875	5.25577E-27	293.9375	8.21087E-32
hsa-miR-30e	4496.813	3.678E-38	7039.813	3.678E-38	3848.063	3.678E-38	4384.188	3.678E-38

hsa-miR-30e*	10069.5	3.678E-38	10906.56	3.678E-38	11648.88	3.678E-38	12562.38	3.678E-38
hsa-miR-31	554.8125	3.678E-38	5519.188	3.678E-38	5019.375	3.678E-38	4844.375	3.678E-38
hsa-miR-31*	1426.125	3.678E-38	2276.313	3.678E-38	165.3125	0.001436311	3755.313	3.678E-38
hsa-miR-32	78.875	0.7129115	100.5	0.08726622	144.3125	3.19733E-07	126.625	3.80626E-05
hsa-miR-32*	589.375	3.678E-38	351.9375	3.678E-38	164.5625	8.75362E-12	653.5	3.678E-38
hsa-miR-320d,hsa-mil	14076.38	3.678E-38	13581.38	3.678E-38	15481.38	3.678E-38	14427.44	3.678E-38
hsa-miR-323-3p	107.75	0.02249852	1889.688	3.678E-38	489.75	3.678E-38	452.3125	3.678E-38
hsa-miR-323-5p	111.8125	0.01139383	110.25	0.03841726	103.125	0.2802483	99.6875	0.2190969
hsa-miR-324-3p	1589.75	3.678E-38	5587.438	3.678E-38	5561.563	3.678E-38	7486.875	3.678E-38
hsa-miR-324-5p	3136.688	3.678E-38	3747.813	3.678E-38	4987.625	3.678E-38	4177.438	3.678E-38
hsa-miR-325	83.5	0.5674524	101.625	0.1804878	108.8125	0.2264495	98.8125	0.219188
hsa-miR-326	906.3125	3.678E-38	141.875	7.13463E-07	2840.938	3.678E-38	4025.938	3.678E-38
hsa-miR-328	2472.938	3.678E-38	4672.063	3.678E-38	4299.313	3.678E-38	7873.813	3.678E-38
hsa-miR-329	218.6875	1.54348E-20	4009.25	3.678E-38	3240.75	3.678E-38	2894.125	3.678E-38
hsa-miR-330-3p	103.1875	0.08610333	269.75	3.678E-38	146.0625	7.1418E-07	94.75	0.3293332
hsa-miR-330-5p	118.9375	0.007759357	122.6875	0.001994928	92.3125	0.4928329	240.8125	3.678E-38
hsa-miR-331-3p	9824.875	3.678E-38	8889.5	3.678E-38	10791.88	3.678E-38	9428.438	3.678E-38
hsa-miR-331-5p	650.1875	3.678E-38	358	3.678E-38	1216.875	3.678E-38	2507.813	3.678E-38
hsa-miR-335	912	3.678E-38	6818.313	3.678E-38	3747.375	3.678E-38	6103.563	3.678E-38
hsa-miR-335*	2169.938	3.678E-38	7668	3.678E-38	2336.375	3.678E-38	4400.5	3.678E-38
hsa-miR-337:9.1	1800.25	3.678E-38	82.5625	0.6121072	211.9375	5.12707E-05	84.8125	0.5507531
hsa-miR-337-3p	134.25	0.000608787	5032.438	3.678E-38	1739.188	3.678E-38	2540.563	3.678E-38
hsa-miR-337-5p	235.5	1.4226E-32	810.25	3.678E-38	125.9375	0.08061454	208.75	6.55234E-23
hsa-miR-338-3p	144	2.9865E-06	1125.188	3.678E-38	184.6875	7.04802E-13	715.625	3.678E-38
hsa-miR-338-5p	90.25	0.3289774	123.3125	0.009256392	105.6875	0.2729603	109.5625	0.08735056
hsa-miR-339-3p	74.6875	0.795634	302.875	3.678E-38	193.25	6.24564E-21	256.0625	3.678E-38
hsa-miR-339-5p	270.5625	5.34675E-12	300.3125	3.678E-38	309.75	3.678E-38	840.875	3.678E-38
hsa-miR-33a	3874.625	3.678E-38	104.6875	0.0651456	91.125	0.5019011	518.9375	3.678E-38
hsa-miR-33a*	122.375	0.002695765	129.6875	0.00360282	109.9375	0.2124861	104.625	0.1480103
hsa-miR-33b	81.8125	0.5399803	603.875	3.678E-38	87.375	0.6583673	177.1875	6.34257E-18
hsa-miR-33b*	83	0.5282972	80.3125	0.7052467	83.3125	0.6674858	91.375	0.3419373
hsa-miR-340	272.5	3.678E-38	1492	3.678E-38	339.75	3.678E-38	646.5625	3.678E-38
hsa-miR-340*	201.5	1.41268E-05	342.375	3.678E-38	690.5	3.678E-38	638.5	3.678E-38
hsa-miR-342-3p	10644.5	3.678E-38	3496.188	3.678E-38	2971.688	3.678E-38	5726.813	3.678E-38
hsa-miR-342-5p	9620.688	3.678E-38	1443.813	3.678E-38	4031.625	3.678E-38	2511.25	3.678E-38
hsa-miR-345	678.125	3.678E-38	126.875	0.001085889	85.125	0.6564637	278.75	4.85682E-26
hsa-miR-345:9.1	201.75	2.82283E-15	290.0625	3.678E-38	162.3125	0.001165643	154.5	6.21413E-05
hsa-miR-346	4118.75	3.678E-38	2429.313	3.678E-38	3827.313	3.678E-38	1705.625	3.678E-38
hsa-miR-34a	133.4375	5.09982E-06	745.125	3.678E-38	2789.938	3.678E-38	1834	3.678E-38
hsa-miR-34a*	177	7.94447E-17	197.1875	3.17892E-19	1731.938	3.678E-38	976.75	3.678E-38
hsa-miR-34b	73.125	0.8063664	499.5625	3.678E-38	422.3125	3.678E-38	527.4375	3.678E-38
hsa-miR-34b*	432.1875	3.678E-38	255.9375	6.4608E-24	424	3.678E-38	257.1875	4.46748E-21
hsa-miR-34c-3p	250.3125	3.678E-38	489.0625	3.678E-38	1885.438	3.678E-38	1758	3.678E-38
hsa-miR-34c-5p	226.3125	6.68789E-16	311.25	3.678E-38	224.6875	5.92533E-16	435.6875	3.678E-38
hsa-miR-361-3p	83.6875	0.5827996	1398.875	3.678E-38	329.5	3.678E-38	1133.438	3.678E-38
hsa-miR-361-5p	6447.125	3.678E-38	8204.063	3.678E-38	8583.188	3.678E-38	6209.5	3.678E-38
hsa-miR-362-3p	610.75	3.678E-38	1256.25	3.678E-38	649.875	3.678E-38	1038	3.678E-38
hsa-miR-362-5p	77.8125	0.7086477	190.25	7.66819E-21	600	3.678E-38	107.625	0.01540618
hsa-miR-363	4492.75	3.678E-38	1520.625	3.678E-38	488.75	3.678E-38	2295.25	3.678E-38
hsa-miR-363*	90.875	0.3591502	84.375	0.6175022	87.5	0.6433145	92.3125	0.4464789
hsa-miR-365	2927.625	3.678E-38	4566.188	3.678E-38	4576.75	3.678E-38	4104.188	3.678E-38
hsa-miR-367	89.6875	0.40042	87.75	0.5082812	95.1875	0.3823863	1861.938	3.678E-38
hsa-miR-367*	403.375	3.678E-38	178.0625	4.54177E-09	281.0625	6.68097E-14	225.9375	6.7324E-15
hsa-miR-369-3p	2425.313	3.678E-38	6210.75	3.678E-38	4663.188	3.678E-38	5406.375	3.678E-38
hsa-miR-369-5p	203.375	7.97694E-20	405	3.678E-38	167.5	0.001181995	328.75	3.678E-38
hsa-miR-370	208	3.11115E-25	7699.875	3.678E-38	689.6875	3.678E-38	2360.125	3.678E-38
hsa-miR-371-3p	113.5	0.090511	100.0625	0.1659852	124.8125	0.0563437	106.8125	0.08231509
hsa-miR-371-5p	932.5	3.678E-38	124.25	0.006155588	564.9375	3.678E-38	238.8125	1.30109E-25
hsa-miR-372	314.4375	3.678E-38	151.625	1.04625E-05	204.5	7.40469E-07	191.75	8.77716E-10
hsa-miR-373	73	0.8418705	74	0.8288257	74.9375	0.7939041	73.5625	0.8325338
hsa-miR-373*	79.6875	0.7116556	77.0625	0.7764508	77.625	0.75942	80.4375	0.6747545
hsa-miR-374a	9535.063	3.678E-38	9726.188	3.678E-38	11931.25	3.678E-38	10719.5	3.678E-38
hsa-miR-374a*	396.3125	3.678E-38	1098.75	3.678E-38	319.125	5.10516E-28	599.125	3.678E-38
hsa-miR-374b	73.1875	0.8170022	73.625	0.8334436	119.125	0.001478169	73.1875	0.8320912
hsa-miR-374b*	79.625	0.6439292	156.625	1.3669E-10	211.5	2.17987E-26	101.5	0.08268663
hsa-miR-375	9298.563	3.678E-38	10064.19	3.678E-38	5431.688	3.678E-38	4381	3.678E-38
hsa-miR-376a	9343.625	3.678E-38	5298.813	3.678E-38	6919.375	3.678E-38	7109.188	3.678E-38
hsa-miR-376a*	78.9375	0.6905031	1629.875	3.678E-38	150.8125	1.60506E-08	917.5	3.678E-38
hsa-miR-376a*:9.1	4441.938	3.678E-38	2920.313	3.678E-38	3382.688	3.678E-38	3282.75	3.678E-38
hsa-miR-376b	5176.75	3.678E-38	4169.813	3.678E-38	4430	3.678E-38	5816.688	3.678E-38
hsa-miR-376c	7928.125	3.678E-38	7752.563	3.678E-38	6293.5	3.678E-38	8560.813	3.678E-38
hsa-miR-377	6963.875	3.678E-38	2758.875	3.678E-38	3391.688	3.678E-38	5086.875	3.678E-38
hsa-miR-377*	93.125	0.3209463	130.9375	5.34969E-05	94.75	0.4226798	114	0.01312705
hsa-miR-378	2405.813	3.678E-38	925.4375	3.678E-38	1202.375	3.678E-38	3105.313	3.678E-38
hsa-miR-378*	3384.125	3.678E-38	561.875	3.678E-38	6721.063	3.678E-38	4334.188	3.678E-38
hsa-miR-379	8826.25	3.678E-38	7248	3.678E-38	6625.625	3.678E-38	9159.063	3.678E-38
hsa-miR-379*	81.1875	0.5960449	2175.375	3.678E-38	83.1875	0.6042635	292.3125	3.678E-38
hsa-miR-380	11933.69	3.678E-38	6074.813	3.678E-38	9211.563	3.678E-38	6445.438	3.678E-38
hsa-miR-380*	3512.5	3.678E-38	1694.5	3.678E-38	2459.625	3.678E-38	2378.438	3.678E-38
hsa-miR-381	369.875	3.678E-38	455.0625	3.678E-38	285	4.57967E-16	222.875	1.02405E-16

hsa-miR-382	87.1875	0.4578606	405.5625	3.678E-38	220.0625	1.3503E-29	653.875	3.678E-38
hsa-miR-383	208.5	3.5481E-19	180.5625	2.12283E-11	398.375	3.678E-38	217.5625	1.20057E-12
hsa-miR-384	152.8125	0.001775632	94.875	0.2784966	860.125	3.678E-38	111.25	0.08279887
hsa-miR-409-3p	84.9375	0.5209762	1710.938	3.678E-38	478.25	3.678E-38	1577.813	3.678E-38
hsa-miR-409-5p	81.75	0.6282925	91.5625	0.3708805	84.375	0.6558282	202.0625	6.85633E-28
hsa-miR-410	2556.938	3.678E-38	2185.313	3.678E-38	638.875	3.678E-38	2976.188	3.678E-38
hsa-miR-411	79.125	0.7012927	4084.688	3.678E-38	1814.875	3.678E-38	2730.5	3.678E-38
hsa-miR-411*	138.8125	1.00597E-06	97.875	0.2203362	96.6875	0.3660744	101.0625	0.1978496
hsa-miR-412	148.625	2.75408E-05	128.625	0.003950241	146.9375	0.01182639	148.9375	0.000145629
hsa-miR-421	121.4375	0.001680044	1101.625	3.678E-38	1746.188	3.678E-38	1979.25	3.678E-38
hsa-miR-422a	77.75	0.7094033	79.3125	0.7164545	79	0.7679042	76.8125	0.7742496
hsa-miR-423-3p	8114.688	3.678E-38	6642.313	3.678E-38	6362.688	3.678E-38	4425.875	3.678E-38
hsa-miR-423-5p	13934.44	3.678E-38	8808.188	3.678E-38	10886.38	3.678E-38	9625.063	3.678E-38
hsa-miR-424	1747.438	3.678E-38	3919.75	3.678E-38	3240.875	3.678E-38	5140.438	3.678E-38
hsa-miR-424*	573.125	3.678E-38	311.3125	3.678E-38	620.25	3.678E-38	1310.438	3.678E-38
hsa-miR-425	1928.75	3.678E-38	1994.75	3.678E-38	3785.438	3.678E-38	2670.25	3.678E-38
hsa-miR-425*	387.125	3.678E-38	217.125	8.94734E-29	235.5	9.85448E-24	4061.25	3.678E-38
hsa-miR-429	5298.125	3.678E-38	11791.81	3.678E-38	12941.44	3.678E-38	8047.5	3.678E-38
hsa-miR-431	120.9375	0.01941156	6360.188	3.678E-38	144.5	0.000541347	347.1875	3.678E-38
hsa-miR-431*	91.25	0.4097113	96.4375	0.2151581	92.25	0.5122086	602.75	3.678E-38
hsa-miR-432	170.875	1.36248E-11	6306.563	3.678E-38	2035.5	3.678E-38	6549	3.678E-38
hsa-miR-432*	82.3125	0.5951856	233.5625	3.678E-38	81.0625	0.7368751	79.875	0.7074782
hsa-miR-433	184.625	5.21116E-19	1900.563	3.678E-38	428.8125	3.678E-38	634.1875	3.678E-38
hsa-miR-448	1414.125	3.678E-38	282.8125	3.678E-38	77.75	0.7683772	182.5	8.56536E-07
hsa-miR-449a	3934.125	3.678E-38	2107.5	3.678E-38	3561.125	3.678E-38	1774.375	3.678E-38
hsa-miR-449b	681.75	3.678E-38	297.125	3.678E-38	583.5625	3.678E-38	438.0625	3.678E-38
hsa-miR-450a	1465.188	3.678E-38	647.5625	3.678E-38	347.5	3.678E-38	1166.25	3.678E-38
hsa-miR-450b-3p	86	0.4072652	81.0625	0.6766499	80.625	0.7174081	90.1875	0.4882372
hsa-miR-450b-5p	1435.688	3.678E-38	389.625	3.678E-38	407	3.678E-38	731.8125	3.678E-38
hsa-miR-451	13419.88	3.678E-38	11435.94	3.678E-38	7347.438	3.678E-38	10570.44	3.678E-38
hsa-miR-452	80.375	0.6511077	989.4375	3.678E-38	592.5	3.678E-38	331.9375	3.678E-38
hsa-miR-452*	179.75	4.133E-13	131.0625	0.001374831	208.5	1.85081E-06	120.875	0.01699659
hsa-miR-452*:9.1	1825.438	3.678E-38	446.9375	3.678E-38	730.6875	3.678E-38	618.125	3.678E-38
hsa-miR-453	92.0625	0.4507529	93.125	0.3306715	102.75	0.2699838	89.6875	0.4363648
hsa-miR-454	4013.125	3.678E-38	4938.063	3.678E-38	3071.625	3.678E-38	3874.375	3.678E-38
hsa-miR-454*	1481.688	3.678E-38	103.3125	0.05913692	459.25	3.678E-38	96.6875	0.1305152
hsa-miR-455-3p	446.25	3.678E-38	12913.75	3.678E-38	11699.44	3.678E-38	11476.56	3.678E-38
hsa-miR-455-5p	154.1875	3.19366E-10	2153.125	3.678E-38	593.4375	3.678E-38	1147.5	3.678E-38
hsa-miR-483-3p	432.5625	3.678E-38	470.125	3.678E-38	6658.375	3.678E-38	2702.188	3.678E-38
hsa-miR-483-5p	104.3125	0.0864092	128.5625	0.001847026	111.5	0.05513846	660.5625	3.678E-38
hsa-miR-484	8402.313	3.678E-38	5525.125	3.678E-38	7219.375	3.678E-38	5151.813	3.678E-38
hsa-miR-485-3p	214.625	3.51266E-28	1414.375	3.678E-38	1211.125	3.678E-38	1210.438	3.678E-38
hsa-miR-485-5p	119.5	0.0085216	1028.938	3.678E-38	257.5625	3.678E-38	372.6875	3.678E-38
hsa-miR-486-3p	128.0625	0.005709748	91.75	0.375844	1619.688	3.678E-38	329.3125	3.678E-38
hsa-miR-486-5p	6892.063	3.678E-38	2710.938	3.678E-38	2498.688	3.678E-38	2923.313	3.678E-38
hsa-miR-487a	90.8125	0.3553139	1130.875	3.678E-38	79.5	0.7232416	220.875	1.95313E-36
hsa-miR-487b	1779.688	3.678E-38	5794.875	3.678E-38	2218.313	3.678E-38	3560.5	3.678E-38
hsa-miR-488	2248.938	3.678E-38	417.25	3.678E-38	82.875	0.6679241	84.5625	0.5912797
hsa-miR-488*	89.4375	0.4200273	88.8125	0.4634286	94.3125	0.4809837	86.1875	0.5540122
hsa-miR-489	77.9375	0.7364475	76.5	0.7796612	75	0.8054231	305.875	2.77109E-29
hsa-miR-490-3p	86.0625	0.5001764	90.0625	0.4519553	101.25	0.1800275	165.5625	2.92072E-05
hsa-miR-490-5p	446.125	3.678E-38	91.6875	0.3751253	85.0625	0.5956651	143.875	2.22484E-07
hsa-miR-491-3p	141.375	0.002308553	106.6875	0.07987289	113.1875	0.1116648	116.25	0.03549441
hsa-miR-491-5p	4147.813	3.678E-38	219.0625	3.06854E-22	3019.938	3.678E-38	1138.688	3.678E-38
hsa-miR-492	620.3125	3.678E-38	83.375	0.6030268	88.1875	0.6272684	85.25	0.6021277
hsa-miR-493	2227.75	3.678E-38	3720.813	3.678E-38	82.125	0.6899018	257.875	3.678E-38
hsa-miR-493*	227.75	7.51419E-24	3122.813	3.678E-38	3530.188	3.678E-38	2630.438	3.678E-38
hsa-miR-494	204.375	2.846E-22	3326.125	3.678E-38	3341.688	3.678E-38	566.1875	3.678E-38
hsa-miR-495	121.1875	0.005396429	2457.875	3.678E-38	337.4375	3.678E-38	612.625	3.678E-38
hsa-miR-496	92	0.3055099	97.6875	0.1605899	84.6875	0.6483192	85.3125	0.5635645
hsa-miR-497	96.0625	0.217427	1015.125	3.678E-38	448.4375	3.678E-38	735.9375	3.678E-38
hsa-miR-497*	493.5625	3.678E-38	74.375	0.8349456	75.625	0.8006009	75.6875	0.7868156
hsa-miR-498	92.5	0.2724801	81.5	0.6578689	85.5625	0.6262377	781.75	3.678E-38
hsa-miR-499-3p	81.9375	0.5922437	78.9375	0.7220067	103.0625	0.112921	81.125	0.6617953
hsa-miR-499-5p	716.25	3.678E-38	2911.875	3.678E-38	4197.938	3.678E-38	2462.563	3.678E-38
hsa-miR-500	294.4375	3.678E-38	960.5	3.678E-38	2034.438	3.678E-38	2132.438	3.678E-38
hsa-miR-501-3p	74.0625	0.8064303	205.375	6.68497E-27	1070.25	3.678E-38	225.625	3.678E-38
hsa-miR-501-5p	85.5625	0.4972489	149.875	5.69005E-09	1045.625	3.678E-38	181.75	3.53776E-19
hsa-miR-502-3p,hsa-r	1057.5	3.678E-38	1651.313	3.678E-38	821.125	3.678E-38	1183.75	3.678E-38
hsa-miR-502-5p	253	1.52216E-14	800.6875	3.678E-38	440.9375	3.678E-38	327.875	3.678E-38
hsa-miR-503	590.75	3.678E-38	859.9375	3.678E-38	1314.375	3.678E-38	2392.938	3.678E-38
hsa-miR-504	9999.438	3.678E-38	6178.375	3.678E-38	8520.875	3.678E-38	3000.688	3.678E-38
hsa-miR-505	85	0.5515099	1565.813	3.678E-38	1402	3.678E-38	763.625	3.678E-38
hsa-miR-505*	1507.625	3.678E-38	456.5	3.678E-38	166.75	7.03114E-13	209.75	1.40491E-32
hsa-miR-506	235.625	7.50918E-19	133.25	9.0311E-05	392.0625	3.678E-38	299.9375	3.678E-38
hsa-miR-507	620.3125	3.678E-38	287.25	1.07832E-36	443.375	3.678E-38	353.5625	3.678E-38
hsa-miR-508-3p	90.8125	0.4222227	114.875	0.0131682	417.5625	3.678E-38	380.125	3.678E-38
hsa-miR-508-5p	106.1875	0.04576591	92.0625	0.361221	98.8125	0.3645836	93.375	0.3506347
hsa-miR-509-3-5p	80.4375	0.6441078	79.125	0.7392051	89.625	0.3868004	73.875	0.809565
hsa-miR-509-3p	94.6875	0.2157798	104.5	0.05706647	1914.75	3.678E-38	713	3.678E-38

hsa-miR-509-5p	93	0.3077403	87.4375	0.5296499	85.0625	0.658445	84.875	0.5880656
hsa-miR-510	78.5	0.69154	81.4375	0.6598974	75.875	0.7878403	78.25	0.737532
hsa-miR-511	96.3125	0.1959103	95.625	0.2925267	91.8125	0.4861921	124.8125	0.000248428
hsa-miR-512-3p	1358.938	3.678E-38	96.8125	0.2864301	144.1875	0.001718894	110.5625	0.08795889
hsa-miR-512-5p	5509.875	3.678E-38	3239.188	3.678E-38	173.6875	3.379E-14	4439.813	3.678E-38
hsa-miR-513:9.1	79.625	0.6907589	76.875	0.7802795	186.75	7.56994E-18	87	0.4244422
hsa-miR-513a-3p	139.375	0.000260654	165.8125	1.70603E-09	98.4375	0.3239397	461.9375	3.678E-38
hsa-miR-513a-5p	506.25	3.678E-38	416.875	3.678E-38	413.8125	2.86904E-35	349.1875	3.678E-38
hsa-miR-513b	88.8125	0.4698159	83.0625	0.6312764	138.3125	4.67944E-06	155.875	2.91818E-11
hsa-miR-513c	94.875	0.2834735	94.8125	0.3223474	107.375	0.3060796	137.5	2.5145E-06
hsa-miR-514	77.0625	0.7410682	420.1875	3.678E-38	396.5625	3.678E-38	789.4375	3.678E-38
hsa-miR-515-3p	514.375	3.678E-38	274.375	8.69986E-33	433.125	3.22831E-36	356.75	3.678E-38
hsa-miR-515-5p	79.125	0.6792821	76.375	0.7872617	83	0.6972157	114	0.1135197
hsa-miR-516a-3p,hsa-	242	9.98792E-26	149.25	2.16394E-05	3535.188	3.678E-38	176.125	1.4858E-07
hsa-miR-516a-5p	329.6875	3.678E-38	223	1.70232E-18	258.8125	4.70998E-12	203.9375	2.82706E-12
hsa-miR-516b	102.1875	0.197314	88.75	0.4796602	157.0625	2.00019E-08	89.25	0.4484812
hsa-miR-517*	84.3125	0.4750029	84.125	0.5516686	112.3125	0.01443636	79.6875	0.7053459
hsa-miR-517a	759.625	3.678E-38	437.25	3.678E-38	1054	3.678E-38	582.75	3.678E-38
hsa-miR-517a,hsa-mil	1587.125	3.678E-38	831.3125	3.678E-38	367.6875	3.678E-38	348.875	3.678E-38
hsa-miR-517c	381.125	3.678E-38	222.5	1.38583E-22	278.875	2.23633E-13	3390.813	3.678E-38
hsa-miR-518a-3p	1303	3.678E-38	595.9375	3.678E-38	1075.313	3.678E-38	821.6875	3.678E-38
hsa-miR-518a-5p,hsa-	89.5	0.3790119	82.5	0.6457472	86.1875	0.6107485	86.0625	0.5691318
hsa-miR-518b	1428.188	3.678E-38	343.25	3.678E-38	128.375	0.000818801	744.1875	3.678E-38
hsa-miR-518c	77	0.7363715	78.75	0.7385264	80.8125	0.7119132	80.125	0.7020794
hsa-miR-518c*	1692.063	3.678E-38	324.5	3.678E-38	564.4375	3.678E-38	417.125	3.678E-38
hsa-miR-518d-3p	595.9375	3.678E-38	271.625	1.05446E-30	416.25	8.70421E-34	318.125	3.678E-38
hsa-miR-518e	156.8125	6.64512E-09	121.6875	0.02285842	105.4375	0.3644906	169.25	8.60711E-06
hsa-miR-518e*,hsa-m	731.875	3.678E-38	88.8125	0.4700693	171.4375	1.47932E-12	89.8125	0.4526828
hsa-miR-518e:9.1	109.5625	0.1305415	101.75	0.1751916	116.4375	0.1314744	121.125	0.02496696
hsa-miR-518f	8570.813	3.678E-38	2104.438	3.678E-38	4215.188	3.678E-38	3778.625	3.678E-38
hsa-miR-518f*,hsa-mi	83.71875	0.6100246	79.53125	0.793547	76.5625	0.874309	78.25	0.8269756
hsa-miR-518f:9.1	80.25	0.6527372	181.875	1.01452E-15	119.1875	0.1632076	104.875	0.1639566
hsa-miR-519a	83.5	0.5411377	80.1875	0.6916378	82.5625	0.6632282	208.5625	7.48744E-32
hsa-miR-519b-3p	1004	3.678E-38	415.6875	3.678E-38	684.75	3.678E-38	493.1875	3.678E-38
hsa-miR-519c-3p	328.9375	1.07858E-20	111.875	0.03324045	128.25	0.03147496	109.8125	0.0664176
hsa-miR-519d	90.5625	0.5298173	92.5	0.2899546	899.9375	3.678E-38	164.125	6.75743E-14
hsa-miR-519e	253.25	3.12759E-33	140.6875	0.000443067	180.125	0.000322508	175	3.46573E-07
hsa-miR-519e*	84.9375	0.5226341	78.625	0.7403094	80	0.7221434	78.8125	0.7331831
hsa-miR-520a-3p	97.875	0.1305732	96.5625	0.2776012	93.9375	0.4306305	92.5625	0.3534868
hsa-miR-520a-5p	110.3125	0.04498991	111.75	0.05104578	138.25	0.02925338	121.1875	0.02255597
hsa-miR-520b,hsa-mil	1480.375	3.678E-38	157.1875	1.45222E-05	171.4375	0.001977699	130.75	0.005841452
hsa-miR-520c-3p,hsa-	1981.5	3.678E-38	1718.313	3.678E-38	132.375	0.05042097	126.0625	0.01607035
hsa-miR-520d:9.1	1773.5	3.678E-38	677.375	3.678E-38	83.0625	0.6448709	82.125	0.6094131
hsa-miR-520d-3p	83	0.5596443	79.5625	0.7127238	102.6875	0.1114893	78.375	0.756676
hsa-miR-520d-5p	93.625	0.3191054	91.4375	0.4092883	91.9375	0.4964635	84.5	0.5749305
hsa-miR-520e	5208.625	3.678E-38	2148.125	3.678E-38	1683.125	3.678E-38	2087.563	3.678E-38
hsa-miR-520f	81.5625	0.6479266	78.6875	0.7250595	79.4375	0.7403398	77.625	0.7703258
hsa-miR-520g	77.5	0.6995134	77.625	0.7595729	163.5	1.95786E-11	1929.313	3.678E-38
hsa-miR-520h,hsa-mil	1380.813	3.678E-38	2244.313	3.678E-38	78.4375	0.7327179	2790.938	3.678E-38
hsa-miR-521	320.625	5.21962E-17	79.5	0.7221233	79.75	0.7466341	270.875	3.678E-38
hsa-miR-522	88.625	0.4526124	88.3125	0.478813	104.6875	0.09541067	95.9375	0.3310367
hsa-miR-523	111.1875	0.07271922	99.4375	0.1870349	230.3125	1.72376E-26	122.5	0.02189885
hsa-miR-524-3p	174	0.000267187	2201	3.678E-38	88.625	0.5755632	588.875	3.678E-38
hsa-miR-524-5p	2169.125	3.678E-38	99.125	0.2222198	101.6875	0.3538136	89.875	0.4661484
hsa-miR-525-3p	2609.875	3.678E-38	281	2.87168E-33	343.875	1.92067E-22	232.875	6.77463E-17
hsa-miR-525-5p	401.375	3.678E-38	645.4375	3.678E-38	298.125	2.20101E-16	280.5	2.12033E-35
hsa-miR-526b	123.3125	0.001616142	102.5	0.1705631	123.125	0.162986	106.375	0.1806617
hsa-miR-526b*	74.6875	0.8026428	76	0.7983382	75.1875	0.8145533	73.875	0.8250326
hsa-miR-526b:9.1	2677.813	3.678E-38	163.25	1.14423E-10	89.5	0.5405343	372.8125	3.678E-38
hsa-miR-532-3p	99.875	0.08103309	2788.75	3.678E-38	1660.625	3.678E-38	2858.313	3.678E-38
hsa-miR-532-5p	2543.875	3.678E-38	2978.625	3.678E-38	930.6875	3.678E-38	2022.5	3.678E-38
hsa-miR-539	428.5	3.678E-38	472.5625	3.678E-38	293.625	3.678E-38	496.875	3.678E-38
hsa-miR-541	151.75	1.86541E-06	105.9375	0.1219868	209.625	4.86185E-11	99.0625	0.2477131
hsa-miR-541*	112.6875	0.08193096	97.625	0.2010074	93.875	0.4288346	90.4375	0.4004194
hsa-miR-542-3p	1150.625	3.678E-38	1356.125	3.678E-38	248.4375	3.678E-38	1743.438	3.678E-38
hsa-miR-542-5p	91.75	0.3013188	1848.375	3.678E-38	256.3125	3.678E-38	98.0625	0.2150298
hsa-miR-543	102.5	0.06532516	210.0625	6.21E-19	121.4375	0.003231258	133.5625	3.80452E-06
hsa-miR-544	26308.75	3.678E-38	26308.75	3.678E-38	24922.06	3.678E-38	26308.75	3.678E-38
hsa-miR-545	94.375	0.226467	92.375	0.3703691	101.5	0.3648285	96.875	0.3005119
hsa-miR-545*	93.8125	0.2934251	150.25	3.05105E-05	168.125	0.001466976	168.8125	1.68201E-06
hsa-miR-545:9.1	1068.375	3.678E-38	1251.25	3.678E-38	1900.813	3.678E-38	1589.188	3.678E-38
hsa-miR-548a-3p	2028.063	3.678E-38	285	3.678E-38	1096.563	3.678E-38	370	3.678E-38
hsa-miR-548a-5p	88.75	0.4148699	84.0625	0.6060499	93	0.3923289	80.0625	0.6958119
hsa-miR-548b-3p	766.3125	3.678E-38	321.625	3.678E-38	534.5625	3.678E-38	619.6875	3.678E-38
hsa-miR-548b-5p	811.9375	3.678E-38	417.1875	3.678E-38	633.875	3.678E-38	467.1875	3.678E-38
hsa-miR-548c-3p	1896.625	3.678E-38	104.9375	0.1134433	109.6875	0.207217	101.6875	0.1918303
hsa-miR-548c-5p	116.9375	0.04262082	219.875	7.02321E-25	407.875	3.678E-38	192.8125	3.25824E-12
hsa-miR-548d-3p	99.375	0.3195164	376.1875	3.678E-38	105.9375	0.09798893	240.625	3.74493E-31
hsa-miR-548d-5p	845.3125	3.678E-38	567	3.678E-38	3934.063	3.678E-38	552.1875	3.678E-38
hsa-miR-548e	91.1875	0.2630545	110.375	0.01586082	129.375	0.000136005	77.4375	0.7627382

hsa-miR-548f	119.625	0.03344002	103.4375	0.1143379	105.5625	0.19248	117.9375	0.03386349
hsa-miR-548g	200.125	1.78929E-24	148.25	7.1167E-05	173.375	0.00052887	204.9375	2.1273E-11
hsa-miR-548h	82.9375	0.610757	77.6875	0.751426	78.6875	0.7639491	461.625	3.678E-38
hsa-miR-548i	109.5	0.03692456	103.875	0.1561317	116.375	0.1908139	94.875	0.3337216
hsa-miR-548j	343.5	3.678E-38	194.125	1.79286E-11	340.375	4.04685E-19	231.625	8.2732E-16
hsa-miR-548k	84.5625	0.5162951	89.6875	0.4665939	93.0625	0.5054475	94.25	0.3957292
hsa-miR-548l	95.0625	0.2883005	89.4375	0.4422619	91.4375	0.5255814	1830.438	3.678E-38
hsa-miR-548m	200.1875	1.59684E-11	146.875	2.95369E-05	179.3125	1.82188E-05	156.75	1.37436E-05
hsa-miR-548n	79.9375	0.596999	78.0625	0.7491419	85.8125	0.5151281	82	0.6722988
hsa-miR-548o	87.8125	0.4871795	80.1875	0.7026543	84.125	0.6864396	81.375	0.6570338
hsa-miR-548p	86.8125	0.4846468	2138.25	3.678E-38	90.25	0.5280188	83.125	0.6027093
hsa-miR-549	161.875	3.32932E-06	93.9375	0.3519646	105.375	0.2753026	105.625	0.1501305
hsa-miR-550	85.1875	0.5913719	80.5	0.6943137	127.3125	0.000264138	92.75	0.2506453
hsa-miR-550*	17385.5	3.678E-38	9840.563	3.678E-38	12818.19	3.678E-38	12872.56	3.678E-38
hsa-miR-551a	8023.688	3.678E-38	5220.875	3.678E-38	2840.938	3.678E-38	4185.625	3.678E-38
hsa-miR-551b	77.125	0.7083987	254.1875	3.678E-38	78.0625	0.7236161	204.6875	1.32383E-29
hsa-miR-551b*	128.5625	3.17188E-05	106.3125	0.1389446	110	0.2696457	90.9375	0.4421744
hsa-miR-552	101.75	0.2334249	88.1875	0.5069084	85.5625	0.6262377	109.25	0.1346496
hsa-miR-553	78.5	0.7074677	90.0625	0.3538329	76.875	0.783019	80.8125	0.6763443
hsa-miR-554	186.9375	4.17653E-06	314.9375	3.678E-38	356.125	2.9647E-29	274.125	2.32076E-29
hsa-miR-555	76.4375	0.7454887	77.0625	0.7629171	75.5	0.7923764	77.9375	0.7470597
hsa-miR-556-3p	608.3125	3.678E-38	75.25	0.8028764	78.375	0.7761666	74.625	0.8126732
hsa-miR-556-5p	75.5	0.755978	148.5	1.45863E-08	78.1875	0.7720432	97.0625	0.1204682
hsa-miR-557	87.3125	0.5945915	610.9375	3.678E-38	81.25	0.6965818	984.25	3.678E-38
hsa-miR-558	819.3125	3.678E-38	227	1.37018E-35	139.25	6.51561E-06	80.9375	0.6947742
hsa-miR-559	92.6875	0.3462299	108.125	0.1054688	92.3125	0.4901832	160.125	2.22821E-11
hsa-miR-560:9.1	178.9375	9.26223E-07	95.75	0.2560909	155.125	0.003571857	157.6875	0.000048191
hsa-miR-561	169.8125	4.4809E-14	143.875	0.0003038	266.8125	1.91604E-09	185.875	8.32317E-08
hsa-miR-562	79.625	0.674445	79.0625	0.7251369	83.625	0.6899484	80.375	0.7212126
hsa-miR-563	336.625	3.678E-38	156.9375	4.21881E-06	222.1875	2.12325E-07	212.875	2.99923E-12
hsa-miR-564	219.0625	1.99296E-21	399.6875	3.678E-38	171.625	0.000188626	180.0625	5.77652E-10
hsa-miR-565:9.1	9642.25	3.678E-38	4040.75	3.678E-38	7357.563	3.678E-38	6109.688	3.678E-38
hsa-miR-566	1476.438	3.678E-38	96.875	0.1866096	86.3125	0.5310196	91.8125	0.4128883
hsa-miR-567	120.1875	0.006709538	111.9375	0.04170867	128.875	0.04355463	106.625	0.1076387
hsa-miR-568	251.6875	3.678E-38	2947.375	3.678E-38	269.3125	1.12677E-26	168.25	4.78053E-06
hsa-miR-569	96.9375	0.2181444	1387.188	3.678E-38	571.8125	3.678E-38	5405.313	3.678E-38
hsa-miR-570	99.875	0.26222	197	8.54997E-21	104.375	0.2910092	3430.938	3.678E-38
hsa-miR-571	334.125	3.678E-38	136.8125	0.000901864	178.75	9.65796E-09	189.25	2.67282E-09
hsa-miR-572	90.9375	0.3614403	109.5625	0.09269462	100.3125	0.1918999	85.75	0.5324861
hsa-miR-573	216.1875	8.74967E-20	140.125	0.000460518	193.0625	7.81906E-05	170.375	1.66983E-06
hsa-miR-574-3p	91.5625	0.3168445	5960.563	3.678E-38	6620.75	3.678E-38	4585.625	3.678E-38
hsa-miR-574-5p	461.8125	3.678E-38	810.625	3.678E-38	852.5625	3.678E-38	394.3125	3.678E-38
hsa-miR-575	74.4375	0.8073684	75.3125	0.8145174	73.0625	0.8457427	75.375	0.829552
hsa-miR-576-3p	219.5625	5.3456E-28	134.0625	0.001770056	222.3125	1.61804E-10	238.8125	5.02757E-26
hsa-miR-576-5p	5068.688	3.678E-38	1546.75	3.678E-38	4238.563	3.678E-38	2172.188	3.678E-38
hsa-miR-577	80.8125	0.6862528	110.6875	0.02575174	90.375	0.530336	89.625	0.4220005
hsa-miR-578	299.5625	3.678E-38	139.625	0.000225092	109.9375	0.09297778	118	0.01020614
hsa-miR-579	103.9375	0.04950823	98.3125	0.191874	84.3125	0.6255667	168.875	5.15917E-15
hsa-miR-580	100.3125	0.1572769	100.75	0.182794	99.25	0.3026329	96.6875	0.2259695
hsa-miR-581	129.0625	0.000723848	106.6875	0.09488299	117.75	0.1292985	120.125	0.01416359
hsa-miR-582-3p	81.6875	0.627552	138.3125	1.27151E-06	78.6875	0.7750558	78.625	0.7293884
hsa-miR-582-5p	97.5625	0.2117056	1580.125	3.678E-38	521.9375	3.678E-38	482.125	3.678E-38
hsa-miR-583	236.0625	1.6612E-29	176.0625	9.74273E-09	261.875	7.19049E-11	184.0625	1.55799E-08
hsa-miR-584	3257.813	3.678E-38	1271.75	3.678E-38	694.9375	3.678E-38	611.6875	3.678E-38
hsa-miR-585	723.1875	3.678E-38	433.4375	3.678E-38	592.3125	3.678E-38	460.875	3.678E-38
hsa-miR-586	2034.563	3.678E-38	84.8125	0.5832092	388.75	3.678E-38	83.25	0.6276259
hsa-miR-587	105.1875	0.1610012	139.4375	4.93399E-06	103.75	0.3156459	106.6875	0.1301701
hsa-miR-588	86.1875	0.5710942	99	0.2397026	178.375	0.001683096	93.25	0.3769953
hsa-miR-589	2699.438	3.678E-38	2593.438	3.678E-38	2673.125	3.678E-38	104.4375	0.1552863
hsa-miR-589*	90.1875	0.4472751	97.1875	0.2727742	96.6875	0.405437	107.4375	0.1253577
hsa-miR-590-3p	134.6875	3.46864E-05	1317.188	3.678E-38	189.875	1.64819E-15	139.25	1.73208E-06
hsa-miR-590-5p	243.9375	6.42276E-38	119.125	0.02001662	197.25	0.000100871	163.875	2.22459E-05
hsa-miR-591	233.25	1.00513E-25	144.0625	0.000155333	159	0.002085454	169.625	9.25309E-07
hsa-miR-592	311.25	3.678E-38	318.3125	3.678E-38	219	1.08328E-07	256.625	7.71628E-29
hsa-miR-593	99.8125	0.1872187	115.1875	0.03744379	109.25	0.2351769	110.3125	0.07856358
hsa-miR-593*	98.9375	0.1341359	302.75	7.60328E-37	343.9375	1.4614E-16	98.875	0.1675051
hsa-miR-594:9.1	10213.06	3.678E-38	1841.688	3.678E-38	3671.063	3.678E-38	3137.188	3.678E-38
hsa-miR-595	744.8125	3.678E-38	765.3125	3.678E-38	150.8125	2.12949E-08	113.5	0.008980711
hsa-miR-596	170.875	2.08711E-12	117.625	0.024479	139.125	0.03066945	447.4375	3.678E-38
hsa-miR-597	138.375	0.000390576	93.875	0.2992066	100	0.3125825	104.5	0.1452328
hsa-miR-598	95.6875	0.2238177	2555.688	3.678E-38	2677.75	3.678E-38	3051.688	3.678E-38
hsa-miR-599	75.875	0.7647967	88.25	0.4135766	77.9375	0.7920811	107.25	0.01533793
hsa-miR-600	130.5625	0.000887515	87.8125	0.506816	93.1875	0.5130692	110	0.1091111
hsa-miR-601	93.1875	0.3246364	83.875	0.6159673	86.5625	0.6186981	86.3125	0.5723742
hsa-miR-602	659.125	3.678E-38	99.75	0.20876	241.75	2.71883E-32	105.75	0.1248161
hsa-miR-603	108.125	0.05887929	168.3125	5.45148E-13	88.3125	0.5600454	399.4375	3.678E-38
hsa-miR-604	77.25	0.7307417	155.25	2.90921E-10	72.625	0.8444479	73.625	0.8402538
hsa-miR-605	156	5.4828E-10	93.75	0.3457421	108.0625	0.309752	90.8125	0.430091
hsa-miR-606	75	0.7729251	75.4375	0.8008419	79.6875	0.7451685	75.125	0.8113985
hsa-miR-607	257.25	3.678E-38	129.5	0.002408113	214.5625	2.95781E-06	184.125	2.22909E-08

hsa-miR-608	80.0625	0.6628097	2417.938	3.678E-38	86.0625	0.5645486	80.6875	0.6767689
hsa-miR-609	734.5	3.678E-38	267.0625	1.40706E-30	370.9375	5.85716E-29	321.875	3.678E-38
hsa-miR-610	1232	3.678E-38	1050.125	3.678E-38	601.125	3.678E-38	146.4375	7.82543E-09
hsa-miR-611	75.125	0.7827646	76.625	0.7702211	77.25	0.7518501	79.125	0.7099112
hsa-miR-612	6083.25	3.678E-38	2498.063	3.678E-38	3285.313	3.678E-38	2787.438	3.678E-38
hsa-miR-613	115.0625	0.01449966	96.3125	0.2787949	97.0625	0.3942228	99.4375	0.2127888
hsa-miR-614	226.8125	1.43665E-20	108.25	0.07411761	293.0625	3.678E-38	120.1875	0.02350637
hsa-miR-615-3p	935.625	3.678E-38	546.5625	3.678E-38	723.8125	3.678E-38	522.6875	3.678E-38
hsa-miR-615-5p	425.5	3.678E-38	312.8125	3.678E-38	462.5	3.678E-38	539.625	3.678E-38
hsa-miR-616	90.3125	0.6067721	76.3125	0.7904701	75.8125	0.7532536	74.0625	0.8279818
hsa-miR-616*	91.625	0.2884827	84.375	0.5823793	85.5	0.6506538	254.1875	3.678E-38
hsa-miR-617	132.9375	0.000077303	89	0.4472883	89.4375	0.5584463	98.5	0.2639418
hsa-miR-618	440.9375	3.678E-38	227.125	2.18772E-18	1484.688	3.678E-38	294.5625	5.67612E-30
hsa-miR-619	3881.563	3.678E-38	94.6875	0.3296767	93.25	0.4950189	92.5	0.4000652
hsa-miR-620	153.75	2.32656E-06	122.3125	0.01829042	137.9375	0.03501049	133.125	0.006711542
hsa-miR-621	91.1875	0.2980881	83.5625	0.6285899	87	0.6395752	204.0625	6.30458E-29
hsa-miR-622	7449.75	3.678E-38	5173.5	3.678E-38	6023.75	3.678E-38	2814.938	3.678E-38
hsa-miR-623	9057.313	3.678E-38	4836.188	3.678E-38	6551.938	3.678E-38	3897.313	3.678E-38
hsa-miR-624	214.3125	1.43685E-18	126.4375	0.004461914	199.375	3.65185E-06	131.4375	0.003716578
hsa-miR-624*	634.25	3.678E-38	214.375	5.30725E-22	1612.063	3.678E-38	3568.063	3.678E-38
hsa-miR-625	5330.313	3.678E-38	1725.25	3.678E-38	2949.125	3.678E-38	2308.688	3.678E-38
hsa-miR-625*	2912	3.678E-38	848.1875	3.678E-38	231.25	1.30199E-10	478.125	3.678E-38
hsa-miR-626	106.3125	0.06256094	98.125	0.2225887	106.375	0.2775544	102	0.1910247
hsa-miR-627	4816.25	3.678E-38	1230.5	3.678E-38	562.5	3.678E-38	1546.25	3.678E-38
hsa-miR-628-3p	171.8125	1.15787E-18	1005.125	3.678E-38	2143	3.678E-38	1791.125	3.678E-38
hsa-miR-628-5p	2410.25	3.678E-38	1074.75	3.678E-38	2686.063	3.678E-38	4964.875	3.678E-38
hsa-miR-629	5889.188	3.678E-38	628.8125	3.678E-38	2018.875	3.678E-38	1893.625	3.678E-38
hsa-miR-629*	2125.875	3.678E-38	319.3125	3.678E-38	459.125	3.678E-38	116.6875	0.001250405
hsa-miR-630	3140.563	3.678E-38	107.1875	0.04555371	86.375	0.5591592	429.25	3.678E-38
hsa-miR-631	2725.875	3.678E-38	95.1875	0.3224305	1952.063	3.678E-38	4061.25	3.678E-38
hsa-miR-632	8080	3.678E-38	3011.875	3.678E-38	4816.25	3.678E-38	3735.688	3.678E-38
hsa-miR-633	1607.813	3.678E-38	88.1875	0.5172694	92.9375	0.5414781	101.8125	0.2428736
hsa-miR-634	142.4375	4.57331E-07	125.75	0.004554185	144.5	0.02662721	145.0625	0.000722869
hsa-miR-635	73.5	0.7921492	74.125	0.8321269	75.75	0.8049837	74.1875	0.8222032
hsa-miR-636	76.3125	0.7415466	111.375	0.01111305	82.1875	0.7278876	76.5	0.7743396
hsa-miR-637	78.4375	0.7167379	77.125	0.7720007	78.5	0.7576309	80.9375	0.6910422
hsa-miR-638	75.0625	0.778495	73.125	0.8370491	75.875	0.8010709	194	1.36004E-25
hsa-miR-639	1511.125	3.678E-38	861.25	3.678E-38	79.1875	0.738715	76.1875	0.7696157
hsa-miR-640	80.8125	0.6027648	83.875	0.592375	81.25	0.7160212	81.875	0.6601712
hsa-miR-641	217.25	3.10795E-18	124.5625	0.003300422	161.3125	0.000277155	144.375	0.000177238
hsa-miR-642	2423.438	3.678E-38	94.5625	0.3176461	100.25	0.2821237	142.875	1.35391E-07
hsa-miR-643	177.4375	2.55742E-11	136	0.00119905	157.3125	0.005135571	366.1875	3.678E-38
hsa-miR-644	1121.75	3.678E-38	97.625	0.1892358	122.25	0.1966566	88.75	0.4820117
hsa-miR-645	89.0625	0.4399323	93.6875	0.3069453	297.625	2.50887E-12	87.5625	0.4130285
hsa-miR-646	5941.625	3.678E-38	426.25	3.678E-38	2412.188	3.678E-38	5361.563	3.678E-38
hsa-miR-647	452.5625	3.678E-38	276.3125	1.7067E-31	383	1.05546E-27	287.625	1.55056E-28
hsa-miR-648	103.0625	0.1549824	85.625	0.5725119	4061.313	3.678E-38	84.625	0.5982561
hsa-miR-649	100.9375	0.1341833	83.8125	0.5945202	88.5	0.5602156	88.5625	0.4890708
hsa-miR-650	91	0.38901	82.0625	0.653213	79.375	0.7268397	82.1875	0.6542047
hsa-miR-651	551.25	3.678E-38	301.9375	3.678E-38	572.25	3.678E-38	349.3125	3.678E-38
hsa-miR-652	1236.625	3.678E-38	1784.125	3.678E-38	755.125	3.678E-38	2758.938	3.678E-38
hsa-miR-653	153.125	2.29419E-06	138.875	0.00035603	147.4375	0.00647348	129.4375	0.00442179
hsa-miR-653:9.1	1723.563	3.678E-38	117.125	0.03334342	171.8125	0.002320054	144.6875	0.001158035
hsa-miR-654-3p	5810.25	3.678E-38	3940.938	3.678E-38	2491.063	3.678E-38	5890.813	3.678E-38
hsa-miR-654-5p	4487.5	3.678E-38	3422.813	3.678E-38	3130.875	3.678E-38	3206.313	3.678E-38
hsa-miR-655	77.625	0.789812	1886.25	3.678E-38	873.125	3.678E-38	1473.563	3.678E-38
hsa-miR-656	77.3125	0.7174882	398.6875	3.678E-38	99.125	0.1249791	357.5625	3.678E-38
hsa-miR-657	323.3125	3.678E-38	133.375	0.001494508	130	0.03688992	198.25	3.69752E-10
hsa-miR-658	564.0625	3.678E-38	80.75	0.6851985	82.375	0.6846163	84.4375	0.5979148
hsa-miR-659	92.6875	0.3072253	94.4375	0.3097214	99.6875	0.3390417	90	0.4310232
hsa-miR-660	351.1875	9.40424E-25	905.625	3.678E-38	4150.188	3.678E-38	810.3125	3.678E-38
hsa-miR-661	104.9375	0.09788763	104.25	0.1325994	125.0625	0.1095109	1132.688	3.678E-38
hsa-miR-662	81.125	0.6412725	76.1875	0.784523	495.3125	3.678E-38	76.875	0.7651948
hsa-miR-663	571.125	3.678E-38	193.75	7.45889E-18	80.625	0.6968527	660.375	3.678E-38
hsa-miR-663b	1123.375	3.678E-38	94.6875	0.2916903	122.1875	0.004245021	221.625	1.41409E-37
hsa-miR-664	9048.688	3.678E-38	8417	3.678E-38	7984.063	3.678E-38	7604.375	3.678E-38
hsa-miR-664*	295.8125	3.678E-38	1216.375	3.678E-38	1606.5	3.678E-38	1860.5	3.678E-38
hsa-miR-665	171.875	6.54327E-09	139.75	0.000282952	145.8125	0.006608014	755.25	3.678E-38
hsa-miR-668	846.8125	3.678E-38	453.875	3.678E-38	1721.375	3.678E-38	266.4375	1.93393E-32
hsa-miR-671:9.1	85.125	0.4901222	112.25	0.009792726	196.75	7.32281E-21	179.5625	1.55828E-19
hsa-miR-671-3p	2373.125	3.678E-38	889.25	3.678E-38	2164.75	3.678E-38	2297.125	3.678E-38
hsa-miR-671-5p	90.75	0.3484093	86.875	0.5265572	89.5	0.5824133	88	0.5039045
hsa-miR-675	98.125	0.1966493	91.9375	0.3639763	99	0.2759475	293.8125	3.678E-38
hsa-miR-7	6549.313	3.678E-38	5790.438	3.678E-38	6174.5	3.678E-38	3832.25	3.678E-38
hsa-miR-708	87.25	0.3998645	7509.875	3.678E-38	6639.25	3.678E-38	5242.688	3.678E-38
hsa-miR-708*	102.25	0.1105	92.125	0.3563806	97	0.3584571	254.1875	3.678E-38
hsa-miR-7-1*	20013.56	3.678E-38	15018.44	3.678E-38	18742.69	3.678E-38	11880	3.678E-38
hsa-miR-7-2*	79.625	0.7009506	140.25	1.26722E-05	332.125	1.5594E-31	106.125	0.01968374
hsa-miR-720	15088.06	3.678E-38	11251.25	3.678E-38	13791.75	3.678E-38	15381.06	3.678E-38
hsa-miR-744	5842.625	3.678E-38	5654.438	3.678E-38	4286.5	3.678E-38	5079.875	3.678E-38

hsa-miR-744*	86.5625	0.5478193	80.1875	0.6896753	81.5	0.6878284	82.75	0.6203502
hsa-miR-760	82.3125	0.5740911	610.1875	3.678E-38	207.8125	4.51071E-25	2282.688	3.678E-38
hsa-miR-765	103.75	0.1263966	128.1875	0.003249105	1339.313	3.678E-38	104.1875	0.1160654
hsa-miR-766	8261.688	3.678E-38	1352.938	3.678E-38	1190.063	3.678E-38	1105.375	3.678E-38
hsa-miR-767-3p	89.9375	0.4050616	190.0625	1.20227E-09	90.6875	0.5103324	86.75	0.5297809
hsa-miR-767-5p	749.125	3.678E-38	3790.313	3.678E-38	123.6875	0.05530444	162	2.43313E-07
hsa-miR-768-3p:11.0	9896.688	3.678E-38	10951.69	3.678E-38	12615.31	3.678E-38	10849.63	3.678E-38
hsa-miR-768-5p:11.0	11213.75	3.678E-38	8453.063	3.678E-38	10380.63	3.678E-38	9746.688	3.678E-38
hsa-miR-769-3p	90.25	0.3580555	345.4375	3.678E-38	464.5625	3.678E-38	392.1875	3.678E-38
hsa-miR-769-5p	1292.063	3.678E-38	2245.938	3.678E-38	950.5625	3.678E-38	587.375	3.678E-38
hsa-miR-770-5p	101.625	0.1229813	89	0.4804612	96.5625	0.4989448	88.3125	0.5122582
hsa-miR-801:9.1	80.5	0.6798015	222.5	1.42668E-20	80.4375	0.7327758	78.375	0.7414592
hsa-miR-802	111.75	0.07401315	1880.688	3.678E-38	109.25	0.09456629	108.6875	0.06047515
hsa-miR-873	91.625	0.3554924	87	0.5132774	115.0625	0.01829489	108.8125	0.02463531
hsa-miR-874	2253.563	3.678E-38	1436.5	3.678E-38	509.6875	3.678E-38	1342.063	3.678E-38
hsa-miR-875-3p	84.125	0.50087	85.4375	0.5600536	88.375	0.5579178	88.25	0.4680353
hsa-miR-875-5p	162.9375	0.000590516	228	7.53993E-25	180.375	5.17022E-06	152.6875	9.93069E-06
hsa-miR-876-3p	3363.438	3.678E-38	1851.188	3.678E-38	3126.063	3.678E-38	117.4375	0.02483275
hsa-miR-876-5p	225	7.67736E-25	159.5625	2.08773E-06	262.25	1.35393E-10	193	1.89933E-09
hsa-miR-877	5952.313	3.678E-38	3718.313	3.678E-38	2619.125	3.678E-38	3589.25	3.678E-38
hsa-miR-877*	463.625	3.678E-38	140.75	6.72336E-07	2213.875	3.678E-38	121.125	0.000201696
hsa-miR-885-3p	93.125	0.3874048	517.4375	3.678E-38	93.5625	0.4816193	202	1.45905E-28
hsa-miR-885-5p	98.625	0.1293802	3164.75	3.678E-38	1527.875	3.678E-38	672.5625	3.678E-38
hsa-miR-886-3p	121.3125	0.003473135	1793.813	3.678E-38	743.25	3.678E-38	1705.188	3.678E-38
hsa-miR-886-5p	84.6875	0.562435	560	3.678E-38	104.0625	0.1346319	198.875	1.33319E-22
hsa-miR-887	96.75	0.2697042	92.6875	0.3285581	88.375	0.5236555	93.625	0.269493
hsa-miR-888	102.5	0.1348683	3765.938	3.678E-38	1736.313	3.678E-38	5723.813	3.678E-38
hsa-miR-888*	79.875	0.7036638	91.875	0.2872871	76.75	0.7861735	119.125	0.000495195
hsa-miR-889	527.5	3.678E-38	3674.688	3.678E-38	3824.188	3.678E-38	2419.688	3.678E-38
hsa-miR-890	629.6875	3.678E-38	393.125	3.678E-38	441.3125	3.678E-38	2294.563	3.678E-38
hsa-miR-891a	72.75	0.8253832	1509.75	3.678E-38	2011.25	3.678E-38	5127.438	3.678E-38
hsa-miR-891b	74.4375	0.817452	74.75	0.8173878	73.3125	0.8364982	226.25	3.678E-38
hsa-miR-892a	79.3125	0.6480281	275.375	3.678E-38	290.125	3.678E-38	2059.563	3.678E-38
hsa-miR-892b	84.6875	0.5787769	496.6875	3.678E-38	397.4375	3.678E-38	2286.063	3.678E-38
hsa-miR-9	80.5625	0.6393523	556.875	3.678E-38	1073.313	3.678E-38	1457.25	3.678E-38
hsa-miR-9*	972.8125	3.678E-38	1379.688	3.678E-38	2529.063	3.678E-38	1203.813	3.678E-38
hsa-miR-920	192.8125	1.39441E-18	143.1875	0.000248431	258.1875	1.91687E-09	172.5625	1.30957E-06
hsa-miR-921	260.4375	1.94622E-21	219.9375	1.11135E-18	336.5625	2.82703E-22	306.5	2.92525E-36
hsa-miR-922	137.5625	0.000663564	1282.438	3.678E-38	136.6875	0.01621523	132.125	0.002092564
hsa-miR-923	5476.75	3.678E-38	5917.75	3.678E-38	4920	3.678E-38	7992.375	3.678E-38
hsa-miR-924	107.3125	0.08811255	92.625	0.357188	108.8125	0.2596907	100.1875	0.2369207
hsa-miR-92a	15965.75	3.678E-38	11066.75	3.678E-38	12544.38	3.678E-38	10485.13	3.678E-38
hsa-miR-92a-1*	4951.375	3.678E-38	541.75	3.678E-38	1143.625	3.678E-38	888.5	3.678E-38
hsa-miR-92a-2*	1178.625	3.678E-38	82	0.6005503	75.3125	0.8062299	76.75	0.7815403
hsa-miR-92b	711.1875	3.678E-38	2051.813	3.678E-38	3402.063	3.678E-38	1781	3.678E-38
hsa-miR-92b*	700.9375	3.678E-38	219.0625	9.68066E-32	1757.688	3.678E-38	384.0625	3.678E-38
hsa-miR-93	12422.31	3.678E-38	8851.875	3.678E-38	6607.563	3.678E-38	5456.375	3.678E-38
hsa-miR-93*	82.5	0.5877678	212.4375	3.27248E-27	85.125	0.6229156	112.9375	0.0326451
hsa-miR-933	98.5	0.1596502	97.25	0.1999018	96	0.3683974	92.125	0.3560152
hsa-miR-934	2085.375	3.678E-38	90	0.4226962	91.4375	0.5030716	91.25	0.4108248
hsa-miR-935	87.1875	0.4185079	233.0625	4.77489E-32	152.3125	0.006805713	337	3.678E-38
hsa-miR-936	1747.5	3.678E-38	326.9375	3.678E-38	488.125	3.678E-38	268.6875	1.15135E-22
hsa-miR-937	81.875	0.6123573	86.375	0.5492809	81.875	0.6997856	85.5625	0.5720314
hsa-miR-938	15186.81	3.678E-38	8831.375	3.678E-38	10060.63	3.678E-38	10460.13	3.678E-38
hsa-miR-939	1006.063	3.678E-38	508.6875	3.678E-38	871.4375	3.678E-38	606.5625	3.678E-38
hsa-miR-940	6210.75	3.678E-38	3295.5	3.678E-38	4458.875	3.678E-38	2845.875	3.678E-38
hsa-miR-941	3722.75	3.678E-38	748.125	3.678E-38	1033.875	3.678E-38	864.8125	3.678E-38
hsa-miR-942	4200.688	3.678E-38	80.75	0.6307373	357.25	3.678E-38	195.8125	1.49885E-26
hsa-miR-943	113.625	0.05809923	100.6875	0.181802	301	7.0405E-13	95.25	0.2984703
hsa-miR-944	3514.813	3.678E-38	4452.063	3.678E-38	4715.063	3.678E-38	2723.688	3.678E-38
hsa-miR-95	4893.313	3.678E-38	2783.938	3.678E-38	4294.5	3.678E-38	2612.313	3.678E-38
hsa-miR-96	98.0625	0.3256715	3382.813	3.678E-38	4404.813	3.678E-38	1198.25	3.678E-38
hsa-miR-96*	98.25	0.1618017	810.1875	3.678E-38	493.125	3.678E-38	111.6875	0.01137602
hsa-miR-98	8242.5	3.678E-38	9237.313	3.678E-38	9818.625	3.678E-38	8383.938	3.678E-38
hsa-miR-99a	907.8125	3.678E-38	9139.313	3.678E-38	7730.438	3.678E-38	6484.375	3.678E-38
hsa-miR-99a*	120.875	0.001608649	205	2.21445E-23	118.4375	0.02640995	131.4375	0.003167701
hsa-miR-99b	717.0625	3.678E-38	5771.375	3.678E-38	4169.5	3.678E-38	3800	3.678E-38
hsa-miR-99b*	227	7.06437E-20	174.25	3.63385E-13	142.75	0.001652861	200.375	2.64718E-11
solexa-1460-671	75.625	0.7824747	158.375	9.34412E-09	1099.938	3.678E-38	888.9375	3.678E-38
solexa-15-44487	496.875	3.678E-38	252.5625	2.72863E-25	388.3125	8.09448E-31	313.375	3.678E-38
solexa-2502-366	88.25	0.5175449	82	0.6380079	84.9375	0.6328038	116.75	0.002386087
solexa-2526-361	98	0.2447553	108.1875	0.0759518	94.625	0.3642892	107.875	0.08820122
solexa-2580-353	87.5625	0.2750279	152.5	8.48592E-10	96.3125	0.1641461	156.0625	9.69843E-10
solexa-2683-338	361.375	2.25285E-21	383	3.678E-38	139.1875	2.47694E-06	164.0625	2.8549E-14
solexa-2952-306	16008.19	3.678E-38	12397.5	3.678E-38	14240.94	3.678E-38	14293.69	3.678E-38
solexa-3022-299	1828.688	3.678E-38	167.875	6.4536E-08	214.6875	1.93926E-07	1395.75	3.678E-38
solexa-3044-295	80.9375	0.6483838	77.5625	0.7566637	81.0625	0.6728261	150.9375	1.32469E-10
solexa-3126-285	83.375	0.6000117	905.5625	3.678E-38	136.5625	1.77941E-05	2008.813	3.678E-38
solexa-3277-272	1026.813	3.678E-38	1279.375	3.678E-38	2866.375	3.678E-38	1257.125	3.678E-38
solexa-3464-254	131.5625	0.001073139	96.0625	0.262163	104.125	0.2802779	98.25	0.240956

solexa-3695-237	1784.063	3.678E-38	84.8125	0.5774063	220.0625	7.38799E-30	79.375	0.7154406
solexa-3793-229	404.6875	3.678E-38	124	0.01090884	188.5	8.53005E-13	275.875	3.678E-38
solexa-3927-221	21755.63	3.678E-38	17190	3.678E-38	20707.81	3.678E-38	22182.25	3.678E-38
solexa-4793-177	5726.813	3.678E-38	245.875	3.86355E-26	1344.125	3.678E-38	3118	3.678E-38
solexa-499-2217	7087.563	3.678E-38	3683.813	3.678E-38	6098.375	3.678E-38	2473.813	3.678E-38
solexa-51-13984	3723.125	3.678E-38	1774.063	3.678E-38	3125.5	3.678E-38	1338.813	3.678E-38
solexa-5169-164	88.1875	0.4096314	95.3125	0.2867463	88.0625	0.6187484	278.5	3.678E-38
solexa-539-2056	306.75	3.678E-38	328.9375	3.678E-38	852.875	3.678E-38	544.8125	3.678E-38
solexa-555-1991	5235.938	3.678E-38	3893.313	3.678E-38	6100.438	3.678E-38	7191.938	3.678E-38
solexa-5620-151	80.1875	0.6672685	113.5	0.007667564	171.875	6.07131E-13	94.1875	0.2195579
solexa-578-1915	2518.938	3.678E-38	1258.063	3.678E-38	620.8125	3.678E-38	2067.625	3.678E-38
solexa-5874-144	94.3125	0.3208532	87.5	0.5267109	392.1875	3.678E-38	97.3125	0.3215626
solexa-603-1846	90.5	0.5290384	160.375	1.89864E-05	1012.313	3.678E-38	77.8125	0.7761907
solexa-6676-127	83.375	0.5919514	79.3125	0.7145658	100.5	0.1947512	85.0625	0.5728447
solexa-7111-119	116	0.00848866	85.375	0.575362	92.8125	0.5418032	89.5625	0.4863586
solexa-7297-115	84.8125	0.6220586	91.6875	0.3203258	81.1875	0.7110738	81.875	0.6621147
solexa-7509-112	772.3125	3.678E-38	199.125	1.94804E-13	247.5	8.81455E-11	588.375	3.678E-38
solexa-7534-111	184.875	2.23888E-15	170.5	8.63357E-08	226.375	7.49412E-08	125.8125	0.008926177
solexa-7764-108	1183	3.678E-38	122.1875	0.01216241	163.375	3.24595E-07	757.625	3.678E-38
solexa-8000-104	182.5	3.53852E-09	148	8.51211E-07	154.1875	0.01782105	118.5	0.03961869
solexa-8048-104	170.375	6.51858E-11	623.125	3.678E-38	1451.063	3.678E-38	2721.75	3.678E-38
solexa-8211-102	12694.06	3.678E-38	10703.19	3.678E-38	11185.5	3.678E-38	9565.625	3.678E-38
solexa-826-1288	362.9375	3.678E-38	529.25	3.678E-38	96.5625	0.222644	147.8125	3.42218E-09
solexa-8926-93	478.125	3.678E-38	221.5	1.58483E-17	483.1875	3.678E-38	218.125	1.04087E-13
solexa-9029-92	1901.125	3.678E-38	1338.5	3.678E-38	2113.063	3.678E-38	1319.25	3.678E-38
solexa-9081-91	1684.063	3.678E-38	475.5	3.678E-38	351.625	3.678E-38	1078.5	3.678E-38
solexa-9124-90	2746	3.678E-38	3473.938	3.678E-38	3817.25	3.678E-38	1892	3.678E-38
solexa-9578-86	207.125	7.65475E-15	134.8125	0.001076779	190	3.14814E-05	153.1875	9.29338E-05
solexa-9655-85	236.1875	5.46491E-26	159.875	1.38012E-08	212.4375	4.5202E-14	213.0625	1.17437E-21
